# Search for supersymmetry in events containing a same-flavour opposite-sign dilepton pair, jets, and large missing transverse momentum in $\sqrt{s}=8$ TeV *pp* collisions with the ATLAS detector

**DOI:** 10.1140/epjc/s10052-015-3518-2

**Published:** 2015-07-08

**Authors:** G. Aad, B. Abbott, J. Abdallah, O. Abdinov, R. Aben, M. Abolins, O. S. AbouZeid, H. Abramowicz, H. Abreu, R. Abreu, Y. Abulaiti, B. S. Acharya, L. Adamczyk, D. L. Adams, J. Adelman, S. Adomeit, T. Adye, A. A. Affolder, T. Agatonovic-Jovin, J. A. Aguilar-Saavedra, M. Agustoni, S. P. Ahlen, F. Ahmadov, G. Aielli, H. Akerstedt, T. P. A. Åkesson, G. Akimoto, A. V. Akimov, G. L. Alberghi, J. Albert, S. Albrand, M. J. Alconada Verzini, M. Aleksa, I. N. Aleksandrov, C. Alexa, G. Alexander, T. Alexopoulos, M. Alhroob, G. Alimonti, L. Alio, J. Alison, S. P. Alkire, B. M. M. Allbrooke, P. P. Allport, A. Aloisio, A. Alonso, F. Alonso, C. Alpigiani, A. Altheimer, B. Alvarez Gonzalez, D. Álvarez Piqueras, M. G. Alviggi, K. Amako, Y. Amaral Coutinho, C. Amelung, D. Amidei, S. P. Amor Dos Santos, A. Amorim, S. Amoroso, N. Amram, G. Amundsen, C. Anastopoulos, L. S. Ancu, N. Andari, T. Andeen, C. F. Anders, G. Anders, K. J. Anderson, A. Andreazza, V. Andrei, S. Angelidakis, I. Angelozzi, P. Anger, A. Angerami, F. Anghinolfi, A. V. Anisenkov, N. Anjos, A. Annovi, M. Antonelli, A. Antonov, J. Antos, F. Anulli, M. Aoki, L. Aperio Bella, G. Arabidze, Y. Arai, J. P. Araque, A. T. H. Arce, F. A. Arduh, J-F. Arguin, S. Argyropoulos, M. Arik, A. J. Armbruster, O. Arnaez, V. Arnal, H. Arnold, M. Arratia, O. Arslan, A. Artamonov, G. Artoni, S. Asai, N. Asbah, A. Ashkenazi, B. Åsman, L. Asquith, K. Assamagan, R. Astalos, M. Atkinson, N. B. Atlay, B. Auerbach, K. Augsten, M. Aurousseau, G. Avolio, B. Axen, M. K. Ayoub, G. Azuelos, M. A. Baak, A. E. Baas, C. Bacci, H. Bachacou, K. Bachas, M. Backes, M. Backhaus, E. Badescu, P. Bagiacchi, P. Bagnaia, Y. Bai, T. Bain, J. T. Baines, O. K. Baker, P. Balek, T. Balestri, F. Balli, E. Banas, Sw. Banerjee, A. A. E. Bannoura, H. S. Bansil, L. Barak, S. P. Baranov, E. L. Barberio, D. Barberis, M. Barbero, T. Barillari, M. Barisonzi, T. Barklow, N. Barlow, S. L. Barnes, B. M. Barnett, R. M. Barnett, Z. Barnovska, A. Baroncelli, G. Barone, A. J. Barr, F. Barreiro, J. Barreiro Guimarães da Costa, R. Bartoldus, A. E. Barton, P. Bartos, A. Bassalat, A. Basye, R. L. Bates, S. J. Batista, J. R. Batley, M. Battaglia, M. Bauce, F. Bauer, H. S. Bawa, J. B. Beacham, M. D. Beattie, T. Beau, P. H. Beauchemin, R. Beccherle, P. Bechtle, H. P. Beck, K. Becker, M. Becker, S. Becker, M. Beckingham, C. Becot, A. J. Beddall, A. Beddall, V. A. Bednyakov, C. P. Bee, L. J. Beemster, T. A. Beermann, M. Begel, J. K. Behr, C. Belanger-Champagne, P. J. Bell, W. H. Bell, G. Bella, L. Bellagamba, A. Bellerive, M. Bellomo, K. Belotskiy, O. Beltramello, O. Benary, D. Benchekroun, M. Bender, K. Bendtz, N. Benekos, Y. Benhammou, E. Benhar Noccioli, J. A. Benitez Garcia, D. P. Benjamin, J. R. Bensinger, S. Bentvelsen, L. Beresford, M. Beretta, D. Berge, E. Bergeaas Kuutmann, N. Berger, F. Berghaus, J. Beringer, C. Bernard, N. R. Bernard, C. Bernius, F. U. Bernlochner, T. Berry, P. Berta, C. Bertella, G. Bertoli, F. Bertolucci, C. Bertsche, D. Bertsche, M. I. Besana, G. J. Besjes, O. Bessidskaia Bylund, M. Bessner, N. Besson, C. Betancourt, S. Bethke, A. J. Bevan, W. Bhimji, R. M. Bianchi, L. Bianchini, M. Bianco, O. Biebel, S. P. Bieniek, M. Biglietti, J. Bilbao De Mendizabal, H. Bilokon, M. Bindi, S. Binet, A. Bingul, C. Bini, C. W. Black, J. E. Black, K. M. Black, D. Blackburn, R. E. Blair, J.-B. Blanchard, J.E. Blanco, T. Blazek, I. Bloch, C. Blocker, W. Blum, U. Blumenschein, G. J. Bobbink, V. S. Bobrovnikov, S. S. Bocchetta, A. Bocci, C. Bock, M. Boehler, J. A. Bogaerts, A. G. Bogdanchikov, C. Bohm, V. Boisvert, T. Bold, V. Boldea, A. S. Boldyrev, M. Bomben, M. Bona, M. Boonekamp, A. Borisov, G. Borissov, S. Borroni, J. Bortfeldt, V. Bortolotto, K. Bos, D. Boscherini, M. Bosman, J. Boudreau, J. Bouffard, E. V. Bouhova-Thacker, D. Boumediene, C. Bourdarios, N. Bousson, A. Boveia, J. Boyd, I. R. Boyko, I. Bozic, J. Bracinik, A. Brandt, G. Brandt, O. Brandt, U. Bratzler, B. Brau, J. E. Brau, H. M. Braun, S. F. Brazzale, K. Brendlinger, A. J. Brennan, L. Brenner, R. Brenner, S. Bressler, K. Bristow, T. M. Bristow, D. Britton, D. Britzger, F. M. Brochu, I. Brock, R. Brock, J. Bronner, G. Brooijmans, T. Brooks, W. K. Brooks, J. Brosamer, E. Brost, J. Brown, P. A. Bruckman de Renstrom, D. Bruncko, R. Bruneliere, A. Bruni, G. Bruni, M. Bruschi, L. Bryngemark, T. Buanes, Q. Buat, P. Buchholz, A. G. Buckley, S. I. Buda, I. A. Budagov, F. Buehrer, L. Bugge, M. K. Bugge, O. Bulekov, H. Burckhart, S. Burdin, B. Burghgrave, S. Burke, I. Burmeister, E. Busato, D. Büscher, V. Büscher, P. Bussey, C. P. Buszello, J. M. Butler, A. I. Butt, C. M. Buttar, J. M. Butterworth, P. Butti, W. Buttinger, A. Buzatu, R. Buzykaev, S. Cabrera Urbán, D. Caforio, O. Cakir, P. Calafiura, A. Calandri, G. Calderini, P. Calfayan, L. P. Caloba, D. Calvet, S. Calvet, R. Camacho Toro, S. Camarda, D. Cameron, L. M. Caminada, R. Caminal Armadans, S. Campana, M. Campanelli, A. Campoverde, V. Canale, A. Canepa, M. Cano Bret, J. Cantero, R. Cantrill, T. Cao, M. D. M. Capeans Garrido, I. Caprini, M. Caprini, M. Capua, R. Caputo, R. Cardarelli, T. Carli, G. Carlino, L. Carminati, S. Caron, E. Carquin, G. D. Carrillo-Montoya, J. R. Carter, J. Carvalho, D. Casadei, M. P. Casado, M. Casolino, E. Castaneda-Miranda, A. Castelli, V. Castillo Gimenez, N. F. Castro, P. Catastini, A. Catinaccio, J. R. Catmore, A. Cattai, J. Caudron, V. Cavaliere, D. Cavalli, M. Cavalli-Sforza, V. Cavasinni, F. Ceradini, B. Cerio, K. Cerny, A. S. Cerqueira, A. Cerri, L. Cerrito, F. Cerutti, M. Cerv, A. Cervelli, S. A. Cetin, A. Chafaq, D. Chakraborty, I. Chalupkova, P. Chang, B. Chapleau, J. D. Chapman, D. G. Charlton, C. C. Chau, C. A. Chavez Barajas, S. Cheatham, A. Chegwidden, S. Chekanov, S. V. Chekulaev, G. A. Chelkov, M. A. Chelstowska, C. Chen, H. Chen, K. Chen, L. Chen, S. Chen, X. Chen, Y. Chen, H. C. Cheng, Y. Cheng, A. Cheplakov, E. Cheremushkina, R. Cherkaoui El Moursli, V. Chernyatin, E. Cheu, L. Chevalier, V. Chiarella, J. T. Childers, G. Chiodini, A. S. Chisholm, R. T. Chislett, A. Chitan, M. V. Chizhov, K. Choi, S. Chouridou, B. K. B. Chow, V. Christodoulou, D. Chromek-Burckhart, M. L. Chu, J. Chudoba, A. J. Chuinard, J. J. Chwastowski, L. Chytka, G. Ciapetti, A. K. Ciftci, D. Cinca, V. Cindro, I. A. Cioara, A. Ciocio, Z. H. Citron, M. Ciubancan, A. Clark, B. L. Clark, P. J. Clark, R. N. Clarke, W. Cleland, C. Clement, Y. Coadou, M. Cobal, A. Coccaro, J. Cochran, L. Coffey, J. G. Cogan, B. Cole, S. Cole, A. P. Colijn, J. Collot, T. Colombo, G. Compostella, P. Conde Muiño, E. Coniavitis, S. H. Connell, I. A. Connelly, S. M. Consonni, V. Consorti, S. Constantinescu, C. Conta, G. Conti, F. Conventi, M. Cooke, B. D. Cooper, A. M. Cooper-Sarkar, K. Copic, T. Cornelissen, M. Corradi, F. Corriveau, A. Corso-Radu, A. Cortes-Gonzalez, G. Cortiana, G. Costa, M. J. Costa, D. Costanzo, D. Côté, G. Cottin, G. Cowan, B. E. Cox, K. Cranmer, G. Cree, S. Crépé-Renaudin, F. Crescioli, W. A. Cribbs, M. Crispin Ortuzar, M. Cristinziani, V. Croft, G. Crosetti, T. Cuhadar Donszelmann, J. Cummings, M. Curatolo, C. Cuthbert, H. Czirr, P. Czodrowski, S. D’Auria, M. D’Onofrio, M. J. Da Cunha Sargedas De Sousa, C. Da Via, W. Dabrowski, A. Dafinca, T. Dai, O. Dale, F. Dallaire, C. Dallapiccola, M. Dam, J. R. Dandoy, A. C. Daniells, M. Danninger, M. Dano Hoffmann, V. Dao, G. Darbo, S. Darmora, J. Dassoulas, A. Dattagupta, W. Davey, C. David, T. Davidek, E. Davies, M. Davies, P. Davison, Y. Davygora, E. Dawe, I. Dawson, R. K. Daya-Ishmukhametova, K. De, R. de Asmundis, S. De Castro, S. De Cecco, N. De Groot, P. de Jong, H. De la Torre, F. De Lorenzi, L. De Nooij, D. De Pedis, A. De Salvo, U. De Sanctis, A. De Santo, J. B. De Vivie De Regie, W. J. Dearnaley, R. Debbe, C. Debenedetti, D. V. Dedovich, I. Deigaard, J. Del Peso, T. Del Prete, D. Delgove, F. Deliot, C. M. Delitzsch, M. Deliyergiyev, A. Dell’Acqua, L. Dell’Asta, M. Dell’Orso, M. Della Pietra, D. della Volpe, M. Delmastro, P. A. Delsart, C. Deluca, D. A. DeMarco, S. Demers, M. Demichev, A. Demilly, S. P. Denisov, D. Derendarz, J. E. Derkaoui, F. Derue, P. Dervan, K. Desch, C. Deterre, P. O. Deviveiros, A. Dewhurst, S. Dhaliwal, A. Di Ciaccio, L. Di Ciaccio, A. Di Domenico, C. Di Donato, A. Di Girolamo, B. Di Girolamo, A. Di Mattia, B. Di Micco, R. Di Nardo, A. Di Simone, R. Di Sipio, D. Di Valentino, C. Diaconu, M. Diamond, F. A. Dias, M. A. Diaz, E. B. Diehl, J. Dietrich, S. Diglio, A. Dimitrievska, J. Dingfelder, F. Dittus, F. Djama, T. Djobava, J. I. Djuvsland, M. A. B. do Vale, D. Dobos, M. Dobre, C. Doglioni, T. Dohmae, J. Dolejsi, Z. Dolezal, B. A. Dolgoshein, M. Donadelli, S. Donati, P. Dondero, J. Donini, J. Dopke, A. Doria, M. T. Dova, A. T. Doyle, E. Drechsler, M. Dris, E. Dubreuil, E. Duchovni, G. Duckeck, O. A. Ducu, D. Duda, A. Dudarev, L. Duflot, L. Duguid, M. Dührssen, M. Dunford, H. Duran Yildiz, M. Düren, A. Durglishvili, D. Duschinger, M. Dwuznik, M. Dyndal, C. Eckardt, K. M. Ecker, W. Edson, N. C. Edwards, W. Ehrenfeld, T. Eifert, G. Eigen, K. Einsweiler, T. Ekelof, M. El Kacimi, M. Ellert, S. Elles, F. Ellinghaus, A. A. Elliot, N. Ellis, J. Elmsheuser, M. Elsing, D. Emeliyanov, Y. Enari, O. C. Endner, M. Endo, R. Engelmann, J. Erdmann, A. Ereditato, G. Ernis, J. Ernst, M. Ernst, S. Errede, E. Ertel, M. Escalier, H. Esch, C. Escobar, B. Esposito, A. I. Etienvre, E. Etzion, H. Evans, A. Ezhilov, L. Fabbri, G. Facini, R. M. Fakhrutdinov, S. Falciano, R. J. Falla, J. Faltova, Y. Fang, M. Fanti, A. Farbin, A. Farilla, T. Farooque, S. Farrell, S. M. Farrington, P. Farthouat, F. Fassi, P. Fassnacht, D. Fassouliotis, A. Favareto, L. Fayard, P. Federic, O. L. Fedin, W. Fedorko, S. Feigl, L. Feligioni, C. Feng, E. J. Feng, H. Feng, A. B. Fenyuk, P. Fernandez Martinez, S. Fernandez Perez, S. Ferrag, J. Ferrando, A. Ferrari, P. Ferrari, R. Ferrari, D. E. Ferreira de Lima, A. Ferrer, D. Ferrere, C. Ferretti, A. Ferretto Parodi, M. Fiascaris, F. Fiedler, A. Filipčič, M. Filipuzzi, F. Filthaut, M. Fincke-Keeler, K. D. Finelli, M. C. N. Fiolhais, L. Fiorini, A. Firan, A. Fischer, C. Fischer, J. Fischer, W. C. Fisher, E. A. Fitzgerald, M. Flechl, I. Fleck, P. Fleischmann, S. Fleischmann, G. T. Fletcher, G. Fletcher, T. Flick, A. Floderus, L. R. Flores Castillo, M. J. Flowerdew, A. Formica, A. Forti, D. Fournier, H. Fox, S. Fracchia, P. Francavilla, M. Franchini, D. Francis, L. Franconi, M. Franklin, M. Fraternali, D. Freeborn, S. T. French, F. Friedrich, D. Froidevaux, J. A. Frost, C. Fukunaga, E. Fullana Torregrosa, B. G. Fulsom, J. Fuster, C. Gabaldon, O. Gabizon, A. Gabrielli, A. Gabrielli, S. Gadatsch, S. Gadomski, G. Gagliardi, P. Gagnon, C. Galea, B. Galhardo, E. J. Gallas, B. J. Gallop, P. Gallus, G. Galster, K. K. Gan, J. Gao, Y. Gao, Y. S. Gao, F. M. Garay Walls, F. Garberson, C. García, J. E. García Navarro, M. Garcia-Sciveres, R. W. Gardner, N. Garelli, V. Garonne, C. Gatti, A. Gaudiello, G. Gaudio, B. Gaur, L. Gauthier, P. Gauzzi, I. L. Gavrilenko, C. Gay, G. Gaycken, E. N. Gazis, P. Ge, Z. Gecse, C. N. P. Gee, D. A. A. Geerts, Ch. Geich-Gimbel, M. P. Geisler, C. Gemme, M. H. Genest, S. Gentile, M. George, S. George, D. Gerbaudo, A. Gershon, H. Ghazlane, N. Ghodbane, B. Giacobbe, S. Giagu, V. Giangiobbe, P. Giannetti, B. Gibbard, S. M. Gibson, M. Gilchriese, T. P. S. Gillam, D. Gillberg, G. Gilles, D. M. Gingrich, N. Giokaris, M. P. Giordani, F. M. Giorgi, F. M. Giorgi, P. F. Giraud, P. Giromini, D. Giugni, C. Giuliani, M. Giulini, B. K. Gjelsten, S. Gkaitatzis, I. Gkialas, E. L. Gkougkousis, L. K. Gladilin, C. Glasman, J. Glatzer, P. C. F. Glaysher, A. Glazov, M. Goblirsch-Kolb, J. R. Goddard, J. Godlewski, S. Goldfarb, T. Golling, D. Golubkov, A. Gomes, R. Gonçalo, J. Goncalves Pinto Firmino Da Costa, L. Gonella, S. González de la Hoz, G. Gonzalez Parra, S. Gonzalez-Sevilla, L. Goossens, P. A. Gorbounov, H. A. Gordon, I. Gorelov, B. Gorini, E. Gorini, A. Gorišek, E. Gornicki, A. T. Goshaw, C. Gössling, M. I. Gostkin, D. Goujdami, A. G. Goussiou, N. Govender, H. M. X. Grabas, L. Graber, I. Grabowska-Bold, P. Grafström, K-J. Grahn, J. Gramling, E. Gramstad, S. Grancagnolo, V. Grassi, V. Gratchev, H. M. Gray, E. Graziani, Z. D. Greenwood, K. Gregersen, I. M. Gregor, P. Grenier, J. Griffiths, A. A. Grillo, K. Grimm, S. Grinstein, Ph. Gris, J.-F. Grivaz, J. P. Grohs, A. Grohsjean, E. Gross, J. Grosse-Knetter, G. C. Grossi, Z. J. Grout, L. Guan, J. Guenther, F. Guescini, D. Guest, O. Gueta, E. Guido, T. Guillemin, S. Guindon, U. Gul, C. Gumpert, J. Guo, S. Gupta, P. Gutierrez, N. G. Gutierrez Ortiz, C. Gutschow, C. Guyot, C. Gwenlan, C. B. Gwilliam, A. Haas, C. Haber, H. K. Hadavand, N. Haddad, P. Haefner, S. Hageböck, Z. Hajduk, H. Hakobyan, M. Haleem, J. Haley, D. Hall, G. Halladjian, G. D. Hallewell, K. Hamacher, P. Hamal, K. Hamano, M. Hamer, A. Hamilton, S. Hamilton, G. N. Hamity, P. G. Hamnett, L. Han, K. Hanagaki, K. Hanawa, M. Hance, P. Hanke, R. Hanna, J. B. Hansen, J. D. Hansen, M. C. Hansen, P. H. Hansen, K. Hara, A. S. Hard, T. Harenberg, F. Hariri, S. Harkusha, R. D. Harrington, P. F. Harrison, F. Hartjes, M. Hasegawa, S. Hasegawa, Y. Hasegawa, A. Hasib, S. Hassani, S. Haug, R. Hauser, L. Hauswald, M. Havranek, C. M. Hawkes, R. J. Hawkings, A. D. Hawkins, T. Hayashi, D. Hayden, C. P. Hays, J. M. Hays, H. S. Hayward, S. J. Haywood, S. J. Head, T. Heck, V. Hedberg, L. Heelan, S. Heim, T. Heim, B. Heinemann, L. Heinrich, J. Hejbal, L. Helary, S. Hellman, D. Hellmich, C. Helsens, J. Henderson, R. C. W. Henderson, Y. Heng, C. Hengler, A. Henrichs, A. M. Henriques Correia, S. Henrot-Versille, G. H. Herbert, Y. Hernández Jiménez, R. Herrberg-Schubert, G. Herten, R. Hertenberger, L. Hervas, G. G. Hesketh, N. P. Hessey, J. W. Hetherly, R. Hickling, E. Higón-Rodriguez, E. Hill, J. C. Hill, K. H. Hiller, S. J. Hillier, I. Hinchliffe, E. Hines, R. R. Hinman, M. Hirose, D. Hirschbuehl, J. Hobbs, N. Hod, M. C. Hodgkinson, P. Hodgson, A. Hoecker, M. R. Hoeferkamp, F. Hoenig, M. Hohlfeld, D. Hohn, T. R. Holmes, T. M. Hong, B. H. Hooberman, L. Hooft van Huysduynen, W. H. Hopkins, Y. Horii, A. J. Horton, J-Y. Hostachy, S. Hou, A. Hoummada, J. Howard, J. Howarth, M. Hrabovsky, I. Hristova, J. Hrivnac, T. Hryn’ova, A. Hrynevich, C. Hsu, P. J. Hsu, S.-C. Hsu, D. Hu, Q. Hu, X. Hu, Y. Huang, Z. Hubacek, F. Hubaut, F. Huegging, T. B. Huffman, E. W. Hughes, G. Hughes, M. Huhtinen, T. A. Hülsing, N. Huseynov, J. Huston, J. Huth, G. Iacobucci, G. Iakovidis, I. Ibragimov, L. Iconomidou-Fayard, E. Ideal, Z. Idrissi, P. Iengo, O. Igonkina, T. Iizawa, Y. Ikegami, K. Ikematsu, M. Ikeno, Y. Ilchenko, D. Iliadis, N. Ilic, Y. Inamaru, T. Ince, P. Ioannou, M. Iodice, K. Iordanidou, V. Ippolito, A. Irles Quiles, C. Isaksson, M. Ishino, M. Ishitsuka, R. Ishmukhametov, C. Issever, S. Istin, J. M. Iturbe Ponce, R. Iuppa, J. Ivarsson, W. Iwanski, H. Iwasaki, J. M. Izen, V. Izzo, S. Jabbar, B. Jackson, M. Jackson, P. Jackson, M. R. Jaekel, V. Jain, K. Jakobs, S. Jakobsen, T. Jakoubek, J. Jakubek, D. O. Jamin, D. K. Jana, E. Jansen, R. W. Jansky, J. Janssen, M. Janus, G. Jarlskog, N. Javadov, T. Javůrek, L. Jeanty, J. Jejelava, G.-Y. Jeng, D. Jennens, P. Jenni, J. Jentzsch, C. Jeske, S. Jézéquel, H. Ji, J. Jia, Y. Jiang, S. Jiggins, J. Jimenez Pena, S. Jin, A. Jinaru, O. Jinnouchi, M. D. Joergensen, P. Johansson, K. A. Johns, K. Jon-And, G. Jones, R. W. L. Jones, T. J. Jones, J. Jongmanns, P. M. Jorge, K. D. Joshi, J. Jovicevic, X. Ju, C. A. Jung, P. Jussel, A. Juste Rozas, M. Kaci, A. Kaczmarska, M. Kado, H. Kagan, M. Kagan, S. J. Kahn, E. Kajomovitz, C. W. Kalderon, S. Kama, A. Kamenshchikov, N. Kanaya, M. Kaneda, S. Kaneti, V. A. Kantserov, J. Kanzaki, B. Kaplan, A. Kapliy, D. Kar, K. Karakostas, A. Karamaoun, N. Karastathis, M. J. Kareem, M. Karnevskiy, S. N. Karpov, Z. M. Karpova, K. Karthik, V. Kartvelishvili, A. N. Karyukhin, L. Kashif, R. D. Kass, A. Kastanas, Y. Kataoka, A. Katre, J. Katzy, K. Kawagoe, T. Kawamoto, G. Kawamura, S. Kazama, V. F. Kazanin, M. Y. Kazarinov, R. Keeler, R. Kehoe, J. S. Keller, J. J. Kempster, H. Keoshkerian, O. Kepka, B. P. Kerševan, S. Kersten, R. A. Keyes, F. Khalil-zada, H. Khandanyan, A. Khanov, A.G. Kharlamov, T. J. Khoo, V. Khovanskiy, E. Khramov, J. Khubua, H. Y. Kim, H. Kim, S. H. Kim, Y. Kim, N. Kimura, O. M. Kind, B. T. King, M. King, R. S. B. King, S. B. King, J. Kirk, A. E. Kiryunin, T. Kishimoto, D. Kisielewska, F. Kiss, K. Kiuchi, O. Kivernyk, E. Kladiva, M. H. Klein, M. Klein, U. Klein, K. Kleinknecht, P. Klimek, A. Klimentov, R. Klingenberg, J. A. Klinger, T. Klioutchnikova, P. F. Klok, E.-E. Kluge, P. Kluit, S. Kluth, E. Kneringer, E. B. F. G. Knoops, A. Knue, D. Kobayashi, T. Kobayashi, M. Kobel, M. Kocian, P. Kodys, T. Koffas, E. Koffeman, L. A. Kogan, S. Kohlmann, Z. Kohout, T. Kohriki, T. Koi, H. Kolanoski, I. Koletsou, A. A. Komar, Y. Komori, T. Kondo, N. Kondrashova, K. Köneke, A. C. König, S. König, T. Kono, R. Konoplich, N. Konstantinidis, R. Kopeliansky, S. Koperny, L. Köpke, A. K. Kopp, K. Korcyl, K. Kordas, A. Korn, A. A. Korol, I. Korolkov, E. V. Korolkova, O. Kortner, S. Kortner, T. Kosek, V. V. Kostyukhin, V. M. Kotov, A. Kotwal, A. Kourkoumeli-Charalampidi, C. Kourkoumelis, V. Kouskoura, A. Koutsman, R. Kowalewski, T. Z. Kowalski, W. Kozanecki, A. S. Kozhin, V. A. Kramarenko, G. Kramberger, D. Krasnopevtsev, M. W. Krasny, A. Krasznahorkay, J. K. Kraus, A. Kravchenko, S. Kreiss, M. Kretz, J. Kretzschmar, K. Kreutzfeldt, P. Krieger, K. Krizka, K. Kroeninger, H. Kroha, J. Kroll, J. Kroseberg, J. Krstic, U. Kruchonak, H. Krüger, N. Krumnack, Z. V. Krumshteyn, A. Kruse, M. C. Kruse, M. Kruskal, T. Kubota, H. Kucuk, S. Kuday, S. Kuehn, A. Kugel, F. Kuger, A. Kuhl, T. Kuhl, V. Kukhtin, Y. Kulchitsky, S. Kuleshov, M. Kuna, T. Kunigo, A. Kupco, H. Kurashige, Y. A. Kurochkin, R. Kurumida, V. Kus, E. S. Kuwertz, M. Kuze, J. Kvita, T. Kwan, D. Kyriazopoulos, A. La Rosa, J. L. La Rosa Navarro, L. La Rotonda, C. Lacasta, F. Lacava, J. Lacey, H. Lacker, D. Lacour, V. R. Lacuesta, E. Ladygin, R. Lafaye, B. Laforge, T. Lagouri, S. Lai, L. Lambourne, S. Lammers, C. L. Lampen, W. Lampl, E. Lançon, U. Landgraf, M. P. J. Landon, V. S. Lang, J. C. Lange, A. J. Lankford, F. Lanni, K. Lantzsch, S. Laplace, C. Lapoire, J. F. Laporte, T. Lari, F. Lasagni Manghi, M. Lassnig, P. Laurelli, W. Lavrijsen, A. T. Law, P. Laycock, O. Le Dortz, E. Le Guirriec, E. Le Menedeu, M. LeBlanc, T. LeCompte, F. Ledroit-Guillon, C. A. Lee, S. C. Lee, L. Lee, G. Lefebvre, M. Lefebvre, F. Legger, C. Leggett, A. Lehan, G. Lehmann Miotto, X. Lei, W. A. Leight, A. Leisos, A. G. Leister, M. A. L. Leite, R. Leitner, D. Lellouch, B. Lemmer, K. J. C. Leney, T. Lenz, B. Lenzi, R. Leone, S. Leone, C. Leonidopoulos, S. Leontsinis, C. Leroy, C. G. Lester, M. Levchenko, J. Levêque, D. Levin, L. J. Levinson, M. Levy, A. Lewis, A. M. Leyko, M. Leyton, B. Li, H. Li, H. L. Li, L. Li, L. Li, S. Li, Y. Li, Z. Liang, H. Liao, B. Liberti, A. Liblong, P. Lichard, K. Lie, J. Liebal, W. Liebig, C. Limbach, A. Limosani, S. C. Lin, T. H. Lin, F. Linde, B. E. Lindquist, J. T. Linnemann, E. Lipeles, A. Lipniacka, M. Lisovyi, T. M. Liss, D. Lissauer, A. Lister, A. M. Litke, B. Liu, D. Liu, J. Liu, J. B. Liu, K. Liu, L. Liu, M. Liu, M. Liu, Y. Liu, M. Livan, A. Lleres, J. Llorente Merino, S. L. Lloyd, F. Lo Sterzo, E. Lobodzinska, P. Loch, W. S. Lockman, F. K. Loebinger, A. E. Loevschall-Jensen, A. Loginov, T. Lohse, K. Lohwasser, M. Lokajicek, B. A. Long, J. D. Long, R. E. Long, K. A. Looper, L. Lopes, D. Lopez Mateos, B. Lopez Paredes, I. Lopez Paz, J. Lorenz, N. Lorenzo Martinez, M. Losada, P. Loscutoff, P. J. Lösel, X. Lou, A. Lounis, J. Love, P. A. Love, N. Lu, H. J. Lubatti, C. Luci, A. Lucotte, F. Luehring, W. Lukas, L. Luminari, O. Lundberg, B. Lund-Jensen, M. Lungwitz, D. Lynn, R. Lysak, E. Lytken, H. Ma, L. L. Ma, G. Maccarrone, A. Macchiolo, C. M. Macdonald, J. Machado Miguens, D. Macina, D. Madaffari, R. Madar, H. J. Maddocks, W. F. Mader, A. Madsen, S. Maeland, T. Maeno, A. Maevskiy, E. Magradze, K. Mahboubi, J. Mahlstedt, C. Maiani, C. Maidantchik, A. A. Maier, T. Maier, A. Maio, S. Majewski, Y. Makida, N. Makovec, B. Malaescu, Pa. Malecki, V. P. Maleev, F. Malek, U. Mallik, D. Malon, C. Malone, S. Maltezos, V. M. Malyshev, S. Malyukov, J. Mamuzic, G. Mancini, B. Mandelli, L. Mandelli, I. Mandić, R. Mandrysch, J. Maneira, A. Manfredini, L. Manhaes de Andrade Filho, J. Manjarres Ramos, A. Mann, P. M. Manning, A. Manousakis-Katsikakis, B. Mansoulie, R. Mantifel, M. Mantoani, L. Mapelli, L. March, G. Marchiori, M. Marcisovsky, C. P. Marino, M. Marjanovic, F. Marroquim, S. P. Marsden, Z. Marshall, L. F. Marti, S. Marti-Garcia, B. Martin, T. A. Martin, V. J. Martin, B. Martin dit Latour, M. Martinez, S. Martin-Haugh, V. S. Martoiu, A. C. Martyniuk, M. Marx, F. Marzano, A. Marzin, L. Masetti, T. Mashimo, R. Mashinistov, J. Masik, A. L. Maslennikov, I. Massa, L. Massa, N. Massol, P. Mastrandrea, A. Mastroberardino, T. Masubuchi, P. Mättig, J. Mattmann, J. Maurer, S. J. Maxfield, D. A. Maximov, R. Mazini, S. M. Mazza, L. Mazzaferro, G. Mc Goldrick, S. P. Mc Kee, A. McCarn, R. L. McCarthy, T. G. McCarthy, N. A. McCubbin, K. W. McFarlane, J. A. Mcfayden, G. Mchedlidze, S. J. McMahon, R. A. McPherson, M. Medinnis, S. Meehan, S. Mehlhase, A. Mehta, K. Meier, C. Meineck, B. Meirose, B. R. Mellado Garcia, F. Meloni, A. Mengarelli, S. Menke, E. Meoni, K. M. Mercurio, S. Mergelmeyer, P. Mermod, L. Merola, C. Meroni, F. S. Merritt, A. Messina, J. Metcalfe, A. S. Mete, C. Meyer, C. Meyer, J-P. Meyer, J. Meyer, R. P. Middleton, S. Miglioranzi, L. Mijović, G. Mikenberg, M. Mikestikova, M. Mikuž, M. Milesi, A. Milic, D. W. Miller, C. Mills, A. Milov, D. A. Milstead, A. A. Minaenko, Y. Minami, I. A. Minashvili, A. I. Mincer, B. Mindur, M. Mineev, Y. Ming, L. M. Mir, T. Mitani, J. Mitrevski, V. A. Mitsou, A. Miucci, P. S. Miyagawa, J. U. Mjörnmark, T. Moa, K. Mochizuki, S. Mohapatra, W. Mohr, S. Molander, R. Moles-Valls, K. Mönig, C. Monini, J. Monk, E. Monnier, J. Montejo Berlingen, F. Monticelli, S. Monzani, R. W. Moore, N. Morange, D. Moreno, M. Moreno Llácer, P. Morettini, M. Morgenstern, M. Morii, V. Morisbak, S. Moritz, A. K. Morley, G. Mornacchi, J. D. Morris, S. S. Mortensen, A. Morton, L. Morvaj, H. G. Moser, M. Mosidze, J. Moss, K. Motohashi, R. Mount, E. Mountricha, S. V. Mouraviev, E. J. W. Moyse, S. Muanza, R. D. Mudd, F. Mueller, J. Mueller, K. Mueller, R. S. P. Mueller, T. Mueller, D. Muenstermann, P. Mullen, Y. Munwes, J. A. Murillo Quijada, W. J. Murray, H. Musheghyan, E. Musto, A. G. Myagkov, M. Myska, O. Nackenhorst, J. Nadal, K. Nagai, R. Nagai, Y. Nagai, K. Nagano, A. Nagarkar, Y. Nagasaka, K. Nagata, M. Nagel, E. Nagy, A. M. Nairz, Y. Nakahama, K. Nakamura, T. Nakamura, I. Nakano, H. Namasivayam, R. F. Naranjo Garcia, R. Narayan, T. Naumann, G. Navarro, R. Nayyar, H. A. Neal, P. Yu. Nechaeva, T. J. Neep, P. D. Nef, A. Negri, M. Negrini, S. Nektarijevic, C. Nellist, A. Nelson, S. Nemecek, P. Nemethy, A. A. Nepomuceno, M. Nessi, M. S. Neubauer, M. Neumann, R. M. Neves, P. Nevski, P. R. Newman, D. H. Nguyen, R. B. Nickerson, R. Nicolaidou, B. Nicquevert, J. Nielsen, N. Nikiforou, A. Nikiforov, V. Nikolaenko, I. Nikolic-Audit, K. Nikolopoulos, J. K. Nilsen, P. Nilsson, Y. Ninomiya, A. Nisati, R. Nisius, T. Nobe, M. Nomachi, I. Nomidis, T. Nooney, S. Norberg, M. Nordberg, O. Novgorodova, S. Nowak, M. Nozaki, L. Nozka, K. Ntekas, G. Nunes Hanninger, T. Nunnemann, E. Nurse, F. Nuti, B. J. O’Brien, F. O’grady, D. C. O’Neil, V. O’Shea, F. G. Oakham, H. Oberlack, T. Obermann, J. Ocariz, A. Ochi, I. Ochoa, S. Oda, S. Odaka, H. Ogren, A. Oh, S. H. Oh, C. C. Ohm, H. Ohman, H. Oide, W. Okamura, H. Okawa, Y. Okumura, T. Okuyama, A. Olariu, S. A. Olivares Pino, D. Oliveira Damazio, E. Oliver Garcia, A. Olszewski, J. Olszowska, A. Onofre, P. U. E. Onyisi, C. J. Oram, M. J. Oreglia, Y. Oren, D. Orestano, N. Orlando, C. Oropeza Barrera, R. S. Orr, B. Osculati, R. Ospanov, G. Otero y Garzon, H. Otono, M. Ouchrif, E. A. Ouellette, F. Ould-Saada, A. Ouraou, K. P. Oussoren, Q. Ouyang, A. Ovcharova, M. Owen, R. E. Owen, V. E. Ozcan, N. Ozturk, K. Pachal, A. Pacheco Pages, C. Padilla Aranda, M. Pagáčová, S. Pagan Griso, E. Paganis, C. Pahl, F. Paige, P. Pais, K. Pajchel, G. Palacino, S. Palestini, M. Palka, D. Pallin, A. Palma, Y. B. Pan, E. Panagiotopoulou, C. E. Pandini, J. G. Panduro Vazquez, P. Pani, S. Panitkin, L. Paolozzi, Th. D. Papadopoulou, K. Papageorgiou, A. Paramonov, D. Paredes Hernandez, M. A. Parker, K. A. Parker, F. Parodi, J. A. Parsons, U. Parzefall, E. Pasqualucci, S. Passaggio, F. Pastore, Fr. Pastore, G. Pásztor, S. Pataraia, N. D. Patel, J. R. Pater, T. Pauly, J. Pearce, B. Pearson, L. E. Pedersen, M. Pedersen, S. Pedraza Lopez, R. Pedro, S. V. Peleganchuk, D. Pelikan, H. Peng, B. Penning, J. Penwell, D. V. Perepelitsa, E. Perez Codina, M. T. Pérez García-Estañ, L. Perini, H. Pernegger, S. Perrella, R. Peschke, V. D. Peshekhonov, K. Peters, R. F. Y. Peters, B. A. Petersen, T. C. Petersen, E. Petit, A. Petridis, C. Petridou, E. Petrolo, F. Petrucci, N. E. Pettersson, R. Pezoa, P. W. Phillips, G. Piacquadio, E. Pianori, A. Picazio, E. Piccaro, M. Piccinini, M. A. Pickering, R. Piegaia, D. T. Pignotti, J. E. Pilcher, A. D. Pilkington, J. Pina, M. Pinamonti, J. L. Pinfold, A. Pingel, B. Pinto, S. Pires, M. Pitt, C. Pizio, L. Plazak, M.-A. Pleier, V. Pleskot, E. Plotnikova, P. Plucinski, D. Pluth, R. Poettgen, L. Poggioli, D. Pohl, G. Polesello, A. Policicchio, R. Polifka, A. Polini, C. S. Pollard, V. Polychronakos, K. Pommès, L. Pontecorvo, B. G. Pope, G. A. Popeneciu, D. S. Popovic, A. Poppleton, S. Pospisil, K. Potamianos, I. N. Potrap, C. J. Potter, C. T. Potter, G. Poulard, J. Poveda, V. Pozdnyakov, P. Pralavorio, A. Pranko, S. Prasad, S. Prell, D. Price, J. Price, L. E. Price, M. Primavera, S. Prince, M. Proissl, K. Prokofiev, F. Prokoshin, E. Protopapadaki, S. Protopopescu, J. Proudfoot, M. Przybycien, E. Ptacek, D. Puddu, E. Pueschel, D. Puldon, M. Purohit, P. Puzo, J. Qian, G. Qin, Y. Qin, A. Quadt, D. R. Quarrie, W. B. Quayle, M. Queitsch-Maitland, D. Quilty, S. Raddum, V. Radeka, V. Radescu, S. K. Radhakrishnan, P. Radloff, P. Rados, F. Ragusa, G. Rahal, S. Rajagopalan, M. Rammensee, C. Rangel-Smith, F. Rauscher, S. Rave, T. Ravenscroft, M. Raymond, A. L. Read, N. P. Readioff, D. M. Rebuzzi, A. Redelbach, G. Redlinger, R. Reece, K. Reeves, L. Rehnisch, H. Reisin, M. Relich, C. Rembser, H. Ren, A. Renaud, M. Rescigno, S. Resconi, O. L. Rezanova, P. Reznicek, R. Rezvani, R. Richter, S. Richter, E. Richter-Was, O. Ricken, M. Ridel, P. Rieck, C. J. Riegel, J. Rieger, M. Rijssenbeek, A. Rimoldi, L. Rinaldi, B. Ristić, E. Ritsch, I. Riu, F. Rizatdinova, E. Rizvi, S. H. Robertson, A. Robichaud-Veronneau, D. Robinson, J. E. M. Robinson, A. Robson, C. Roda, S. Roe, O. Røhne, S. Rolli, A. Romaniouk, M. Romano, S. M. Romano Saez, E. Romero Adam, N. Rompotis, M. Ronzani, L. Roos, E. Ros, S. Rosati, K. Rosbach, P. Rose, P. L. Rosendahl, O. Rosenthal, V. Rossetti, E. Rossi, L. P. Rossi, R. Rosten, M. Rotaru, I. Roth, J. Rothberg, D. Rousseau, C. R. Royon, A. Rozanov, Y. Rozen, X. Ruan, F. Rubbo, I. Rubinskiy, V. I. Rud, C. Rudolph, M. S. Rudolph, F. Rühr, A. Ruiz-Martinez, Z. Rurikova, N. A. Rusakovich, A. Ruschke, H. L. Russell, J. P. Rutherfoord, N. Ruthmann, Y. F. Ryabov, M. Rybar, G. Rybkin, N. C. Ryder, A. F. Saavedra, G. Sabato, S. Sacerdoti, A. Saddique, H. F-W. Sadrozinski, R. Sadykov, F. Safai Tehrani, M. Saimpert, H. Sakamoto, Y. Sakurai, G. Salamanna, A. Salamon, M. Saleem, D. Salek, P. H. Sales De Bruin, D. Salihagic, A. Salnikov, J. Salt, D. Salvatore, F. Salvatore, A. Salvucci, A. Salzburger, D. Sampsonidis, A. Sanchez, J. Sánchez, V. Sanchez Martinez, H. Sandaker, R. L. Sandbach, H. G. Sander, M. P. Sanders, M. Sandhoff, C. Sandoval, R. Sandstroem, D. P. C. Sankey, M. Sannino, A. Sansoni, C. Santoni, R. Santonico, H. Santos, I. Santoyo Castillo, K. Sapp, A. Sapronov, J. G. Saraiva, B. Sarrazin, O. Sasaki, Y. Sasaki, K. Sato, G. Sauvage, E. Sauvan, G. Savage, P. Savard, C. Sawyer, L. Sawyer, J. Saxon, C. Sbarra, A. Sbrizzi, T. Scanlon, D. A. Scannicchio, M. Scarcella, V. Scarfone, J. Schaarschmidt, P. Schacht, D. Schaefer, R. Schaefer, J. Schaeffer, S. Schaepe, S. Schaetzel, U. Schäfer, A. C. Schaffer, D. Schaile, R. D. Schamberger, V. Scharf, V. A. Schegelsky, D. Scheirich, M. Schernau, C. Schiavi, C. Schillo, M. Schioppa, S. Schlenker, E. Schmidt, K. Schmieden, C. Schmitt, S. Schmitt, S. Schmitt, B. Schneider, Y. J. Schnellbach, U. Schnoor, L. Schoeffel, A. Schoening, B. D. Schoenrock, E. Schopf, A. L. S. Schorlemmer, M. Schott, D. Schouten, J. Schovancova, S. Schramm, M. Schreyer, C. Schroeder, N. Schuh, M. J. Schultens, H.-C. Schultz-Coulon, H. Schulz, M. Schumacher, B. A. Schumm, Ph. Schune, C. Schwanenberger, A. Schwartzman, T. A. Schwarz, Ph. Schwegler, Ph. Schwemling, R. Schwienhorst, J. Schwindling, T. Schwindt, M. Schwoerer, F. G. Sciacca, E. Scifo, G. Sciolla, F. Scuri, F. Scutti, J. Searcy, G. Sedov, E. Sedykh, P. Seema, S. C. Seidel, A. Seiden, F. Seifert, J. M. Seixas, G. Sekhniaidze, S. J. Sekula, K. E. Selbach, D. M. Seliverstov, N. Semprini-Cesari, C. Serfon, L. Serin, L. Serkin, T. Serre, R. Seuster, H. Severini, T. Sfiligoj, F. Sforza, A. Sfyrla, E. Shabalina, M. Shamim, L. Y. Shan, R. Shang, J. T. Shank, M. Shapiro, P. B. Shatalov, K. Shaw, S. M. Shaw, A. Shcherbakova, C. Y. Shehu, P. Sherwood, L. Shi, S. Shimizu, C. O. Shimmin, M. Shimojima, M. Shiyakova, A. Shmeleva, D. Shoaleh Saadi, M. J. Shochet, S. Shojaii, S. Shrestha, E. Shulga, M. A. Shupe, S. Shushkevich, P. Sicho, O. Sidiropoulou, D. Sidorov, A. Sidoti, F. Siegert, Dj. Sijacki, J. Silva, Y. Silver, S. B. Silverstein, V. Simak, O. Simard, Lj. Simic, S. Simion, E. Simioni, B. Simmons, D. Simon, R. Simoniello, P. Sinervo, N. B. Sinev, G. Siragusa, A. N. Sisakyan, S. Yu. Sivoklokov, J. Sjölin, T. B. Sjursen, M. B. Skinner, H. P. Skottowe, P. Skubic, M. Slater, T. Slavicek, M. Slawinska, K. Sliwa, V. Smakhtin, B. H. Smart, L. Smestad, S. Yu. Smirnov, Y. Smirnov, L. N. Smirnova, O. Smirnova, M. N. K. Smith, M. Smizanska, K. Smolek, A. A. Snesarev, G. Snidero, S. Snyder, R. Sobie, F. Socher, A. Soffer, D. A. Soh, C. A. Solans, M. Solar, J. Solc, E. Yu. Soldatov, U. Soldevila, A. A. Solodkov, A. Soloshenko, O. V. Solovyanov, V. Solovyev, P. Sommer, H. Y. Song, N. Soni, A. Sood, A. Sopczak, B. Sopko, V. Sopko, V. Sorin, D. Sosa, M. Sosebee, C. L. Sotiropoulou, R. Soualah, P. Soueid, A. M. Soukharev, D. South, S. Spagnolo, M. Spalla, F. Spanò, W. R. Spearman, F. Spettel, R. Spighi, G. Spigo, L. A. Spiller, M. Spousta, T. Spreitzer, R. D. St. Denis, S. Staerz, J. Stahlman, R. Stamen, S. Stamm, E. Stanecka, C. Stanescu, M. Stanescu-Bellu, M. M. Stanitzki, S. Stapnes, E. A. Starchenko, J. Stark, P. Staroba, P. Starovoitov, R. Staszewski, P. Stavina, P. Steinberg, B. Stelzer, H. J. Stelzer, O. Stelzer-Chilton, H. Stenzel, S. Stern, G. A. Stewart, J. A. Stillings, M. C. Stockton, M. Stoebe, G. Stoicea, P. Stolte, S. Stonjek, A. R. Stradling, A. Straessner, M. E. Stramaglia, J. Strandberg, S. Strandberg, A. Strandlie, E. Strauss, M. Strauss, P. Strizenec, R. Ströhmer, D. M. Strom, R. Stroynowski, A. Strubig, S. A. Stucci, B. Stugu, N. A. Styles, D. Su, J. Su, R. Subramaniam, A. Succurro, Y. Sugaya, C. Suhr, M. Suk, V. V. Sulin, S. Sultansoy, T. Sumida, S. Sun, X. Sun, J. E. Sundermann, K. Suruliz, G. Susinno, M. R. Sutton, S. Suzuki, Y. Suzuki, M. Svatos, S. Swedish, M. Swiatlowski, I. Sykora, T. Sykora, D. Ta, C. Taccini, K. Tackmann, J. Taenzer, A. Taffard, R. Tafirout, N. Taiblum, H. Takai, R. Takashima, H. Takeda, T. Takeshita, Y. Takubo, M. Talby, A. A. Talyshev, J. Y. C. Tam, K. G. Tan, J. Tanaka, R. Tanaka, S. Tanaka, S. Tanaka, B. B. Tannenwald, N. Tannoury, S. Tapprogge, S. Tarem, F. Tarrade, G. F. Tartarelli, P. Tas, M. Tasevsky, T. Tashiro, E. Tassi, A. Tavares Delgado, Y. Tayalati, F. E. Taylor, G. N. Taylor, W. Taylor, F. A. Teischinger, M. Teixeira Dias Castanheira, P. Teixeira-Dias, K. K. Temming, H. Ten Kate, P. K. Teng, J. J. Teoh, F. Tepel, S. Terada, K. Terashi, J. Terron, S. Terzo, M. Testa, R. J. Teuscher, J. Therhaag, T. Theveneaux-Pelzer, J. P. Thomas, J. Thomas-Wilsker, E. N. Thompson, P. D. Thompson, R. J. Thompson, A. S. Thompson, L. A. Thomsen, E. Thomson, M. Thomson, R. P. Thun, M. J. Tibbetts, R. E. Ticse Torres, V. O. Tikhomirov, Yu. A. Tikhonov, S. Timoshenko, E. Tiouchichine, P. Tipton, S. Tisserant, T. Todorov, S. Todorova-Nova, J. Tojo, S. Tokár, K. Tokushuku, K. Tollefson, E. Tolley, L. Tomlinson, M. Tomoto, L. Tompkins, K. Toms, E. Torrence, H. Torres, E. Torró Pastor, J. Toth, F. Touchard, D. R. Tovey, T. Trefzger, L. Tremblet, A. Tricoli, I. M. Trigger, S. Trincaz-Duvoid, M. F. Tripiana, W. Trischuk, B. Trocmé, C. Troncon, M. Trottier-McDonald, M. Trovatelli, P. True, M. Trzebinski, A. Trzupek, C. Tsarouchas, J. C-L. Tseng, P. V. Tsiareshka, D. Tsionou, G. Tsipolitis, N. Tsirintanis, S. Tsiskaridze, V. Tsiskaridze, E. G. Tskhadadze, I. I. Tsukerman, V. Tsulaia, S. Tsuno, D. Tsybychev, A. Tudorache, V. Tudorache, A. N. Tuna, S. A. Tupputi, S. Turchikhin, D. Turecek, R. Turra, A. J. Turvey, P. M. Tuts, A. Tykhonov, M. Tylmad, M. Tyndel, I. Ueda, R. Ueno, M. Ughetto, M. Ugland, M. Uhlenbrock, F. Ukegawa, G. Unal, A. Undrus, G. Unel, F. C. Ungaro, Y. Unno, C. Unverdorben, J. Urban, P. Urquijo, P. Urrejola, G. Usai, A. Usanova, L. Vacavant, V. Vacek, B. Vachon, C. Valderanis, N. Valencic, S. Valentinetti, A. Valero, L. Valery, S. Valkar, E. Valladolid Gallego, S. Vallecorsa, J. A. Valls Ferrer, W. Van Den Wollenberg, P. C. Van Der Deijl, R. van der Geer, H. van der Graaf, R. Van Der Leeuw, N. van Eldik, P. van Gemmeren, J. Van Nieuwkoop, I. van Vulpen, M. C. van Woerden, M. Vanadia, W. Vandelli, R. Vanguri, A. Vaniachine, F. Vannucci, G. Vardanyan, R. Vari, E. W. Varnes, T. Varol, D. Varouchas, A. Vartapetian, K. E. Varvell, F. Vazeille, T. Vazquez Schroeder, J. Veatch, F. Veloso, T. Velz, S. Veneziano, A. Ventura, D. Ventura, M. Venturi, N. Venturi, A. Venturini, V. Vercesi, M. Verducci, W. Verkerke, J. C. Vermeulen, A. Vest, M. C. Vetterli, O. Viazlo, I. Vichou, T. Vickey, O. E. Vickey Boeriu, G. H. A. Viehhauser, S. Viel, R. Vigne, M. Villa, M. Villaplana Perez, E. Vilucchi, M. G. Vincter, V. B. Vinogradov, I. Vivarelli, F. Vives Vaque, S. Vlachos, D. Vladoiu, M. Vlasak, M. Vogel, P. Vokac, G. Volpi, M. Volpi, H. von der Schmitt, H. von Radziewski, E. von Toerne, V. Vorobel, K. Vorobev, M. Vos, R. Voss, J. H. Vossebeld, N. Vranjes, M. Vranjes Milosavljevic, V. Vrba, M. Vreeswijk, R. Vuillermet, I. Vukotic, Z. Vykydal, P. Wagner, W. Wagner, H. Wahlberg, S. Wahrmund, J. Wakabayashi, J. Walder, R. Walker, W. Walkowiak, C. Wang, F. Wang, H. Wang, H. Wang, J. Wang, J. Wang, K. Wang, R. Wang, S. M. Wang, T. Wang, X. Wang, C. Wanotayaroj, A. Warburton, C. P. Ward, D. R. Wardrope, M. Warsinsky, A. Washbrook, C. Wasicki, P. M. Watkins, A. T. Watson, I. J. Watson, M. F. Watson, G. Watts, S. Watts, B. M. Waugh, S. Webb, M. S. Weber, S. W. Weber, J. S. Webster, A. R. Weidberg, B. Weinert, J. Weingarten, C. Weiser, H. Weits, P. S. Wells, T. Wenaus, T. Wengler, S. Wenig, N. Wermes, M. Werner, P. Werner, M. Wessels, J. Wetter, K. Whalen, A. M. Wharton, A. White, M. J. White, R. White, S. White, D. Whiteson, F. J. Wickens, W. Wiedenmann, M. Wielers, P. Wienemann, C. Wiglesworth, L. A. M. Wiik-Fuchs, A. Wildauer, H. G. Wilkens, H. H. Williams, S. Williams, C. Willis, S. Willocq, A. Wilson, J. A. Wilson, I. Wingerter-Seez, F. Winklmeier, B. T. Winter, M. Wittgen, J. Wittkowski, S. J. Wollstadt, M. W. Wolter, H. Wolters, B. K. Wosiek, J. Wotschack, M. J. Woudstra, K. W. Wozniak, M. Wu, M. Wu, S. L. Wu, X. Wu, Y. Wu, T. R. Wyatt, B. M. Wynne, S. Xella, D. Xu, L. Xu, B. Yabsley, S. Yacoob, R. Yakabe, M. Yamada, Y. Yamaguchi, A. Yamamoto, S. Yamamoto, T. Yamanaka, K. Yamauchi, Y. Yamazaki, Z. Yan, H. Yang, H. Yang, Y. Yang, L. Yao, W-M. Yao, Y. Yasu, E. Yatsenko, K. H. Yau Wong, J. Ye, S. Ye, I. Yeletskikh, A. L. Yen, E. Yildirim, K. Yorita, R. Yoshida, K. Yoshihara, C. Young, C. J. S. Young, S. Youssef, D. R. Yu, J. Yu, J. M. Yu, J. Yu, L. Yuan, A. Yurkewicz, I. Yusuff, B. Zabinski, R. Zaidan, A. M. Zaitsev, J. Zalieckas, A. Zaman, S. Zambito, L. Zanello, D. Zanzi, C. Zeitnitz, M. Zeman, A. Zemla, K. Zengel, O. Zenin, T. Ženiš, D. Zerwas, D. Zhang, F. Zhang, J. Zhang, L. Zhang, R. Zhang, X. Zhang, Z. Zhang, X. Zhao, Y. Zhao, Z. Zhao, A. Zhemchugov, J. Zhong, B. Zhou, C. Zhou, L. Zhou, L. Zhou, N. Zhou, C. G. Zhu, H. Zhu, J. Zhu, Y. Zhu, X. Zhuang, K. Zhukov, A. Zibell, D. Zieminska, N. I. Zimine, C. Zimmermann, R. Zimmermann, S. Zimmermann, Z. Zinonos, M. Zinser, M. Ziolkowski, L. Živković, G. Zobernig, A. Zoccoli, M. zur Nedden, G. Zurzolo, L. Zwalinski, ATLAS Publications

**Affiliations:** Department of Physics, University of Adelaide, Adelaide, Australia; Physics Department, SUNY Albany, Albany, NY USA; Department of Physics, University of Alberta, Edmonton, AB Canada; Department of Physics, Ankara University, Ankara, Turkey; LAPP, CNRS/IN2P3 and Université Savoie Mont Blanc, Annecy-le-Vieux, France; High Energy Physics Division, Argonne National Laboratory, Argonne, IL USA; Department of Physics, University of Arizona, Tucson, AZ USA; Department of Physics, The University of Texas at Arlington, Arlington, TX USA; Physics Department, University of Athens, Athens, Greece; Physics Department, National Technical University of Athens, Zografou, Greece; Institute of Physics, Azerbaijan Academy of Sciences, Baku, Azerbaijan; Institut de Física d’Altes Energies and Departament de Física de la Universitat Autònoma de Barcelona, Barcelona, Spain; Institute of Physics, University of Belgrade, Belgrade, Serbia; Department for Physics and Technology, University of Bergen, Bergen, Norway; Physics Division, Lawrence Berkeley National Laboratory and University of California, Berkeley, CA USA; Department of Physics, Humboldt University, Berlin, Germany; Albert Einstein Center for Fundamental Physics and Laboratory for High Energy Physics, University of Bern, Bern, Switzerland; School of Physics and Astronomy, University of Birmingham, Birmingham, UK; Department of Physics, Bogazici University, Istanbul, Turkey; INFN Sezione di Bologna, Bologna, Italy; Physikalisches Institut, University of Bonn, Bonn, Germany; Department of Physics, Boston University, Boston, MA USA; Department of Physics, Brandeis University, Waltham, MA USA; Universidade Federal do Rio De Janeiro COPPE/EE/IF, Rio de Janeiro, Brazil; Physics Department, Brookhaven National Laboratory, Upton, NY USA; National Institute of Physics and Nuclear Engineering, Bucharest, Romania; Departamento de Física, Universidad de Buenos Aires, Buenos Aires, Argentina; Cavendish Laboratory, University of Cambridge, Cambridge, UK; Department of Physics, Carleton University, Ottawa, ON Canada; CERN, Geneva, Switzerland; Enrico Fermi Institute, University of Chicago, Chicago, IL USA; Departamento de Física, Pontificia Universidad Católica de Chile, Santiago, Chile; Institute of High Energy Physics, Chinese Academy of Sciences, Beijing, China; Laboratoire de Physique Corpusculaire, Clermont Université and Université Blaise Pascal and CNRS/IN2P3, Clermont-Ferrand, France; Nevis Laboratory, Columbia University, Irvington, NY USA; Niels Bohr Institute, University of Copenhagen, Copenhagen, Denmark; INFN Gruppo Collegato di Cosenza, Laboratori Nazionali di Frascati, Frascati, Italy; Faculty of Physics and Applied Computer Science, AGH University of Science and Technology, Kraków, Poland; Institute of Nuclear Physics, Polish Academy of Sciences, Kraków, Poland; Physics Department, Southern Methodist University, Dallas, TX USA; Physics Department, University of Texas at Dallas, Richardson, TX USA; DESY, Hamburg and Zeuthen, Germany; Institut für Experimentelle Physik IV, Technische Universität Dortmund, Dortmund, Germany; Institut für Kern- und Teilchenphysik, Technische Universität Dresden, Dresden, Germany; Department of Physics, Duke University, Durham, NC USA; SUPA, School of Physics and Astronomy, University of Edinburgh, Edinburgh, UK; INFN Laboratori Nazionali di Frascati, Frascati, Italy; Fakultät für Mathematik und Physik, Albert-Ludwigs-Universität, Freiburg, Germany; Section de Physique, Université de Genève, Geneva, Switzerland; INFN Sezione di Genova, Genoa, Italy; E. Andronikashvili Institute of Physics, Iv. Javakhishvili, Tbilisi State University, Tbilisi, Georgia; II Physikalisches Institut, Justus-Liebig-Universität Giessen, Giessen, Germany; SUPA, School of Physics and Astronomy, University of Glasgow, Glasgow, UK; II Physikalisches Institut, Georg-August-Universität, Göttingen, Germany; Laboratoire de Physique Subatomique et de Cosmologie, Université Grenoble-Alpes, CNRS/IN2P3, Grenoble, France; Department of Physics, Hampton University, Hampton, VA USA; Laboratory for Particle Physics and Cosmology, Harvard University, Cambridge, MA USA; Kirchhoff-Institut für Physik, Ruprecht-Karls-Universität Heidelberg, Heidelberg, Germany; Faculty of Applied Information Science, Hiroshima Institute of Technology, Hiroshima, Japan; Department of Physics, The Chinese University of Hong Kong, Clear Water Bay, Kowloon, Shatin, NT Hong Kong; Department of Physics, Indiana University, Bloomington, IN USA; Institut für Astro- und Teilchenphysik, Leopold-Franzens-Universität, Innsbruck, Austria; University of Iowa, Iowa City, IA USA; Department of Physics and Astronomy, Iowa State University, Ames, IA USA; Joint Institute for Nuclear Research, JINR Dubna, Dubna, Russia; KEK, High Energy Accelerator Research Organization, Tsukuba, Japan; Graduate School of Science, Kobe University, Kobe, Japan; Faculty of Science, Kyoto University, Kyoto, Japan; Kyoto University of Education, Kyoto, Japan; Department of Physics, Kyushu University, Fukuoka, Japan; Instituto de Física La Plata, Universidad Nacional de La Plata and CONICET, La Plata, Argentina; Physics Department, Lancaster University, Lancaster, UK; INFN Sezione di Lecce, Lecce, Italy; Oliver Lodge Laboratory, University of Liverpool, Liverpool, UK; Department of Physics, Jožef Stefan Institute and University of Ljubljana, Ljubljana, Slovenia; School of Physics and Astronomy, Queen Mary University of London, London, UK; Department of Physics, Royal Holloway University of London, Surrey, UK; Department of Physics and Astronomy, University College London, London, UK; Louisiana Tech University, Ruston, LA USA; Laboratoire de Physique Nucléaire et de Hautes Energies, UPMC and Université Paris-Diderot and CNRS/IN2P3, Paris, France; Fysiska institutionen, Lunds universitet, Lund, Sweden; Departamento de Fisica Teorica C-15, Universidad Autonoma de Madrid, Madrid, Spain; Institut für Physik, Universität Mainz, Mainz, Germany; School of Physics and Astronomy, University of Manchester, Manchester, UK; CPPM, Aix-Marseille Université and CNRS/IN2P3, Marseille, France; Department of Physics, University of Massachusetts, Amherst, MA USA; Department of Physics, McGill University, Montreal, QC Canada; School of Physics, University of Melbourne, Melbourne, VIC Australia; Department of Physics, The University of Michigan, Ann Arbor, MI USA; Department of Physics and Astronomy, Michigan State University, East Lansing, MI USA; INFN Sezione di Milano, Milan, Italy; B.I. Stepanov Institute of Physics, National Academy of Sciences of Belarus, Minsk, Republic of Belarus; National Scientific and Educational Centre for Particle and High Energy Physics, Minsk, Republic of Belarus; Department of Physics, Massachusetts Institute of Technology, Cambridge, MA USA; Group of Particle Physics, University of Montreal, Montreal, QC Canada; P.N. Lebedev Institute of Physics, Academy of Sciences, Moscow, Russia; Institute for Theoretical and Experimental Physics (ITEP), Moscow, Russia; National Research Nuclear University MEPhI, Moscow, Russia; D.V. Skobeltsyn Institute of Nuclear Physics, M.V. Lomonosov Moscow State University, Moscow, Russia; Fakultät für Physik, Ludwig-Maximilians-Universität München, Munich, Germany; Max-Planck-Institut für Physik (Werner-Heisenberg-Institut), Munich, Germany; Nagasaki Institute of Applied Science, Nagasaki, Japan; Graduate School of Science and Kobayashi-Maskawa Institute, Nagoya University, Nagoya, Japan; INFN Sezione di Napoli, Naples, Italy; Department of Physics and Astronomy, University of New Mexico, Albuquerque, NM USA; Institute for Mathematics, Astrophysics and Particle Physics, Radboud University Nijmegen/Nikhef, Nijmegen, The Netherlands; Nikhef National Institute for Subatomic Physics and University of Amsterdam, Amsterdam, The Netherlands; Department of Physics, Northern Illinois University, De Kalb, IL USA; Budker Institute of Nuclear Physics, SB RAS, Novosibirsk, Russia; Department of Physics, New York University, New York, NY USA; Ohio State University, Columbus, OH USA; Faculty of Science, Okayama University, Okayama, Japan; Homer L. Dodge Department of Physics and Astronomy, University of Oklahoma, Norman, OK USA; Department of Physics, Oklahoma State University, Stillwater, OK USA; Palacký University, RCPTM, Olomouc, Czech Republic; Center for High Energy Physics, University of Oregon, Eugene, OR USA; LAL, Université Paris-Sud and CNRS/IN2P3, Orsay, France; Graduate School of Science, Osaka University, Osaka, Japan; Department of Physics, University of Oslo, Oslo, Norway; Department of Physics, Oxford University, Oxford, UK; INFN Sezione di Pavia, Pavia, Italy; Department of Physics, University of Pennsylvania, Philadelphia, PA USA; Petersburg Nuclear Physics Institute, Gatchina, Russia; INFN Sezione di Pisa, Pisa, Italy; Department of Physics and Astronomy, University of Pittsburgh, Pittsburgh, PA USA; Laboratorio de Instrumentacao e Fisica Experimental de Particulas, LIP, Lisbon, Portugal; Institute of Physics, Academy of Sciences of the Czech Republic, Prague, Czech Republic; Czech Technical University in Prague, Prague, Czech Republic; Faculty of Mathematics and Physics, Charles University in Prague, Prague, Czech Republic; State Research Center Institute for High Energy Physics, Protvino, Russia; Particle Physics Department, Rutherford Appleton Laboratory, Didcot, UK; Ritsumeikan University, Kusatsu, Shiga Japan; INFN Sezione di Roma, Rome, Italy; INFN Sezione di Roma Tor Vergata, Rome, Italy; INFN Sezione di Roma Tre, Rome, Italy; Faculté des Sciences Ain Chock, Réseau Universitaire de Physique des Hautes Energies-Université Hassan II, Casablanca, Morocco; DSM/IRFU (Institut de Recherches sur les Lois Fondamentales de l’Univers), CEA Saclay (Commissariat à l’Energie Atomique et aux Energies Alternatives), Gif-sur-Yvette, France; Santa Cruz Institute for Particle Physics, University of California Santa Cruz, Santa Cruz, CA USA; Department of Physics, University of Washington, Seattle, WA USA; Department of Physics and Astronomy, University of Sheffield, Sheffield, UK; Department of Physics, Shinshu University, Nagano, Japan; Fachbereich Physik, Universität Siegen, Siegen, Germany; Department of Physics, Simon Fraser University, Burnaby, BC Canada; SLAC National Accelerator Laboratory, Stanford, CA USA; Faculty of Mathematics, Physics and Informatics, Comenius University, Bratislava, Slovak Republic; Department of Physics, University of Cape Town, Cape Town, South Africa; Department of Physics, Stockholm University, Stockholm, Sweden; Physics Department, Royal Institute of Technology, Stockholm, Sweden; Departments of Physics and Astronomy and Chemistry, Stony Brook University, Stony Brook, NY USA; Department of Physics and Astronomy, University of Sussex, Brighton, UK; School of Physics, University of Sydney, Sydney, Australia; Institute of Physics, Academia Sinica, Taipei, Taiwan; Department of Physics, Technion: Israel Institute of Technology, Haifa, Israel; Raymond and Beverly Sackler School of Physics and Astronomy, Tel Aviv University, Tel Aviv, Israel; Department of Physics, Aristotle University of Thessaloniki, Thessaloníki, Greece; International Center for Elementary Particle Physics and Department of Physics, The University of Tokyo, Tokyo, Japan; Graduate School of Science and Technology, Tokyo Metropolitan University, Tokyo, Japan; Department of Physics, Tokyo Institute of Technology, Tokyo, Japan; Department of Physics, University of Toronto, Toronto, ON Canada; TRIUMF, Vancouver, BC Canada; Faculty of Pure and Applied Sciences, University of Tsukuba, Tsukuba, Japan; Department of Physics and Astronomy, Tufts University, Medford, MA USA; Centro de Investigaciones, Universidad Antonio Narino, Bogotá, Colombia; Department of Physics and Astronomy, University of California Irvine, Irvine, CA USA; INFN Gruppo Collegato di Udine, Sezione di Trieste, Udine, Italy; Department of Physics, University of Illinois, Urbana, IL USA; Department of Physics and Astronomy, University of Uppsala, Uppsala, Sweden; Instituto de Física Corpuscular (IFIC) and Departamento de Física Atómica, Molecular y Nuclear and Departamento de Ingeniería Electrónica and Instituto de Microelectrónica de Barcelona (IMB-CNM), University of Valencia and CSIC, Valencia, Spain; Department of Physics, University of British Columbia, Vancouver, BC Canada; Department of Physics and Astronomy, University of Victoria, Victoria, BC Canada; Department of Physics, University of Warwick, Coventry, UK; Waseda University, Tokyo, Japan; Department of Particle Physics, The Weizmann Institute of Science, Rehovot, Israel; Department of Physics, University of Wisconsin, Madison, WI USA; Fakultät für Physik und Astronomie, Julius-Maximilians-Universität, Würzburg, Germany; Fachbereich C Physik, Bergische Universität Wuppertal, Wuppertal, Germany; Department of Physics, Yale University, New Haven, CT USA; Yerevan Physics Institute, Yerevan, Armenia; Centre de Calcul de l’Institut National de Physique Nucléaire et de Physique des Particules (IN2P3), Villeurbanne, France; CERN, Geneva, Switzerland; Division of Physics, TOBB University of Economics and Technology, Ankara, Turkey; Department of Physics, Dogus University, Istanbul, Turkey; Department of Physics Engineering, Gaziantep University, Gaziantep, Turkey; Dipartimento di Fisica e Astronomia, Università di Bologna, Bologna, Italy; Electrical Circuits Department, Federal University of Juiz de Fora (UFJF), Juiz de Fora, Brazil; Federal University of Sao Joao del Rei (UFSJ), Sao Joao del Rei, Brazil; Instituto de Fisica, Universidade de Sao Paulo, São Paulo, Brazil; Physics Department, National Institute for Research and Development of Isotopic and Molecular Technologies, Cluj Napoca, Romania; University Politehnica Bucharest, Bucharest, Romania; University Politehnica Bucharest, Bucharest, Romania; West University in Timisoara, Timisoara, Romania; Departamento de Física, Universidad Técnica Federico Santa María, Valparaiso, Chile; Department of Modern Physics, University of Science and Technology of China, Anhui, China; Department of Physics, Nanjing University, Jiangsu, China; School of Physics, Shandong University, Shandong, China; Department of Physics and Astronomy, Shanghai Key Laboratory for Particle Physics and Cosmology, Shanghai Jiao Tong University, Shanghai, China; Physics Department, Tsinghua University, Beijing, 100084 China; Dipartimento di Fisica, Università della Calabria, Rende, Italy; Marian Smoluchowski Institute of Physics, Jagiellonian University, Kraków, Poland; Dipartimento di Fisica, Università di Genova, Genoa, Italy; High Energy Physics Institute, Tbilisi State University, Tbilisi, Georgia; Physikalisches Institut, Ruprecht-Karls-Universität Heidelberg, Heidelberg, Germany; ZITI Institut für technische Informatik, Ruprecht-Karls-Universität Heidelberg, Mannheim, Germany; Department of Physics, The University of Hong Kong, Pok Fu Lam, Hong Kong; Department of Physics, The Hong Kong University of Science and Technology, Clear Water Bay, Kowloon, Hong Kong, China; Dipartimento di Matematica e Fisica, Università del Salento, Lecce, Italy; Dipartimento di Fisica, Università di Milano, Milan, Italy; Dipartimento di Fisica, Università di Napoli, Naples, Italy; Dipartimento di Fisica, Università di Pavia, Pavia, Italy; Dipartimento di Fisica E. Fermi, Università di Pisa, Pisa, Italy; Faculdade de Ciências, Universidade de Lisboa, Lisbon, Portugal; Department of Physics, University of Coimbra, Coimbra, Portugal; Centro de Física Nuclear da Universidade de Lisboa, Lisbon, Portugal; Departamento de Fisica, Universidade do Minho, Braga, Portugal; Departamento de Fisica Teorica y del Cosmos and CAFPE, Universidad de Granada, Granada, Spain; Dep Fisica and CEFITEC of Faculdade de Ciencias e Tecnologia, Universidade Nova de Lisboa, Caparica, Portugal; Dipartimento di Fisica, Sapienza Università di Roma, Rome, Italy; Dipartimento di Fisica, Università di Roma Tor Vergata, Rome, Italy; Dipartimento di Matematica e Fisica, Università Roma Tre, Rome, Italy; Centre National de l’Energie des Sciences Techniques Nucleaires, Rabat, Morocco; Faculté des Sciences Semlalia, Université Cadi Ayyad, LPHEA-Marrakech, Marrakech, Morocco; Faculté des Sciences, Université Mohamed Premier and LPTPM,, Oujda, Morocco; Faculté des Sciences, Université Mohammed V-Agdal, Rabat, Morocco; Department of Subnuclear Physics, Institute of Experimental Physics of the Slovak Academy of Sciences, Kosice, Slovak Republic; Department of Physics, University of Johannesburg, Johannesburg, South Africa; School of Physics, University of the Witwatersrand, Johannesburg, South Africa; The Oskar Klein Centre, Stockholm, Sweden; Department of Physics and Astronomy, York University, Toronto, ON Canada; ICTP, Trieste, Italy; Dipartimento di Chimica, Fisica e Ambiente, Università di Udine, Udine, Italy

## Abstract

Two searches for supersymmetric particles in final states containing a same-flavour opposite-sign lepton pair, jets and large missing transverse momentum are presented. The proton–proton collision data used in these searches were collected at a centre-of-mass energy $\sqrt{s}=8$ TeV by the ATLAS detector at the Large Hadron Collider and corresponds to an integrated luminosity of 20.3 fb$^{-1}$. Two leptonic production mechanisms are considered: decays of squarks and gluinos with *Z* bosons in the final state, resulting in a peak in the dilepton invariant mass distribution around the *Z*-boson mass; and decays of neutralinos (e.g. $\tilde{\chi }^{0}_{2} \rightarrow \ell ^{+}\ell ^{-}\tilde{\chi }^{0}_{1}$), resulting in a kinematic endpoint in the dilepton invariant mass distribution. For the former, an excess of events above the expected Standard Model background is observed, with a significance of three standard deviations. In the latter case, the data are well-described by the expected Standard Model background. The results from each channel are interpreted in the context of several supersymmetric models involving the production of squarks and gluinos.

## Introduction

Supersymmetry (SUSY) [1–9] is an extension to the Standard Model (SM) that introduces supersymmetric particles (sparticles), which differ by half a unit of spin from their SM partners. The squarks ($\tilde{q}$) and sleptons ($\tilde{\ell }$) are the scalar partners of the quarks and leptons, and the gluinos ($\tilde{g}$) are the fermionic partners of the gluons. The charginos (${\tilde{\chi }}_{i}^{\pm }$ with $i=1,2$) and neutralinos (${\tilde{\chi }}_{i}^{0}$ with $i=1,2,3,4$) are the mass eigenstates (ordered from the lightest to the heaviest) formed from the linear superpositions of the SUSY partners of the Higgs and electroweak gauge bosons. SUSY models in which the gluino, higgsino and top squark masses are not much higher than the ${\mathrm {TeV}}$ scale can provide a solution to the SM hierarchy problem [10–15].

If strongly interacting sparticles have masses not higher than the ${\mathrm {TeV}}$ scale, they should be produced with observable rates at the Large Hadron Collider (LHC). In the minimal supersymmetric extension of the SM, such particles decay into jets, possibly leptons, and the lightest sparticle (LSP). If the LSP is stable due to R-parity conservation [15–19] and only weakly interacting, it escapes detection, leading to missing transverse momentum ($\mathbf {p}_{\mathrm {T}}^\mathrm {miss}$ and its magnitude ${E_{\mathrm{T}}^{\mathrm{miss}}}$) in the final state. In this scenario, the LSP is a dark-matter candidate [20, 21].

Leptons may be produced in the cascade decays of squarks and gluinos via several mechanisms. Here two scenarios that always produce leptons (electrons or muons) in same-flavour opposite-sign (SFOS) pairs are considered: the leptonic decay of a *Z* boson, $Z\rightarrow \ell ^+\ell ^-$, and the decay $\tilde{\chi }_{2}^{0}\rightarrow \ell ^+\ell ^-\tilde{\chi }_{1}^{0}$, which includes contributions from $\tilde{\chi }_{2}^{0} \rightarrow \tilde{\ell }^{\pm (*)}\ell ^\mp \rightarrow \ell ^+\ell ^-\tilde{\chi }_{1}^{0}$ and $\tilde{\chi }_{2}^{0} \rightarrow Z^*\tilde{\chi }_{1}^{0} \rightarrow \ell ^+\ell ^-\tilde{\chi }_{1}^{0}$. In models with generalised gauge-mediated (GGM) supersymmetry breaking with a gravitino LSP ($\tilde{G}$), *Z* bosons may be produced via the decay $\tilde{\chi }_{1}^{0} \rightarrow Z \tilde{G}$. *Z* bosons may also result from the decay $\tilde{\chi }_{2}^{0} \rightarrow Z \tilde{\chi }_{1}^{0}$, although the GGM interpretation with the decay $\tilde{\chi }_{1}^{0} \rightarrow Z \tilde{G}$ is the focus of the *Z* boson final-state channels studied here. The $\tilde{\chi }_{2}^{0}$ particle may itself be produced in the decays of the squarks or gluinos, e.g. $\tilde{q} \rightarrow q \tilde{\chi }_{2}^{0}$ and $\tilde{g} \rightarrow q \bar{q} \tilde{\chi }_{2}^{0}$.

These two SFOS lepton production modes are distinguished by their distributions of dilepton invariant mass ($m_{\ell \ell }$). The decay $Z\rightarrow \ell ^+\ell ^-$ leads to a peak in the $m_{\ell \ell }$ distribution around the *Z* boson mass, while the decay $\tilde{\chi }_{2}^{0}\rightarrow \ell ^+\ell ^-\tilde{\chi }_{1}^{0}$ leads to a rising distribution in $m_{\ell \ell }$ that terminates at a kinematic endpoint (“edge”) [22], because events with larger ${m_{\ell \ell }}$ values would violate energy conservation in the decay of the $\tilde{\chi }_{2}^{0}$ particle. In this paper, two searches are performed that separately target these two signatures. A search for events with a SFOS lepton pair consistent with originating from the decay of a *Z* boson (on-*Z* search) targets SUSY models with *Z* boson production. A search for events with a SFOS lepton pair inconsistent with *Z* boson decay (off-*Z* search) targets the decay $\tilde{\chi }_{2}^{0}\rightarrow \ell ^+\ell ^-\tilde{\chi }_{1}^{0}$.

Previous searches for physics beyond the Standard Model (BSM) in the $Z\,+\,\mathrm {jets}\,+\,{E_{\mathrm{T}}^{\mathrm{miss}}}$ final state have been performed by the CMS Collaboration [23, 24]. Searches for a dilepton mass edge have also been performed by the CMS Collaboration [24, 25]. In the CMS analysis performed with $\sqrt{s}=8$ ${\mathrm {TeV}}$ data reported in Ref. [24], an excess of events above the SM background with a significance of 2.6 standard deviations was observed.

In this paper, the analysis is performed on the full 2012 ATLAS [26] dataset at a centre-of-mass energy of 8 ${\mathrm {TeV}}$, corresponding to an integrated luminosity of 20.3  fb$^{-1}$.

## The ATLAS detector

ATLAS is a multi-purpose detector consisting of a tracking system, electromagnetic and hadronic calorimeters and a muon system. The tracking system comprises an inner detector (ID) immersed in a 2 T axial field supplied by the central solenoid magnet surrounding it. This sub-detector provides position and momentum measurements of charged particles over the pseudorapidity^1^ range $|\eta |<2.5$. The electromagnetic calorimetry is provided by liquid argon (LAr) sampling calorimeters using lead absorbers, covering the central region ($|\eta |<3.2$). Hadronic calorimeters in the barrel region ($|\eta |<1.7$) use scintillator tiles with steel absorbers, while the pseudorapidity range $1.5<|\eta |<4.9$ is covered using LAr technology with copper or tungsten absorbers. The muon spectrometer (MS) has coverage up to $|\eta |<2.7$ and is built around the three superconducting toroid magnet systems. The MS uses various technologies to provide muon tracking and identification as well as dedicated muon triggering for the range $|\eta |<2.4$.

The trigger system [27] comprises three levels. The first of these (L1) is a hardware-based trigger that uses only a subset of calorimeter and muon system information. Following this, both the second level (L2) and event filter (EF) triggers, constituting the software-based high-level trigger, include fully reconstructed event information to identify objects. At L2, only the regions of interest in $\eta$–$\phi$ identified at L1 are scrutinised, whereas complete event information from all detector sub-systems is available at the EF.

## Data and Monte Carlo samples

The data used in this analysis were collected by ATLAS during 2012. Following requirements based on beam and detector conditions and data quality, the complete dataset corresponds to an integrated luminosity of 20.3 fb$^{-1}$, with an associated uncertainty of 2.8 %. The uncertainty is derived following the same methodology as that detailed in Ref. [28].

Dedicated high-transverse-momentum (${p_{\mathrm{T}}}$) single-lepton triggers are used in conjunction with the lower-${p_{\mathrm{T}}}$ dilepton triggers to increase the trigger efficiency at high lepton ${p_{\mathrm{T}}}$. The required leading-lepton ${p_{\mathrm{T}}}$ threshold is 25 ${\mathrm {GeV}}$, whereas the sub-leading lepton threshold can be as low as 10 ${\mathrm {GeV}}$, depending on the lepton ${p_{\mathrm{T}}}$ threshold of the trigger responsible for accepting the event. To provide an estimate of the efficiency for the lepton selections used in these analyses, trigger efficiencies are calculated using $t\bar{t}$ Monte Carlo (MC) simulated event samples for leptons with ${p_{\mathrm{T}}} >14 {\mathrm {GeV}}$. For events where both leptons are in the barrel (endcaps), the total efficiency of the trigger configuration for a two-lepton selection is approximately 96, 88 and 80 % (91, 92 and 82 %) for *ee*, $e\mu$ and $\mu \mu$ events, respectively. Although the searches in this paper probe only same-flavour final states for evidence of SUSY, the $e\mu$ channel is used to select control samples in data for background estimation purposes.

Simulated event samples are used to validate the analysis techniques and aid in the estimation of SM backgrounds, as well as to provide predictions for BSM signal processes. The SM background samples [29–40] used are listed in Table 1, as are the parton distribution function (PDF) set, underlying-event tune and cross-section calculation order in $\alpha _{\mathrm{s}}$ used to normalise the event yields for these samples. Samples generated with MadGraph5 1.3.28 [41] are interfaced with Pythia 6.426 [42] to simulate the parton shower. All samples generated using Powheg [43–45] use Pythia to simulate the parton shower, with the exception of the diboson samples, which use Pythia8 [46]. Sherpa [47] simulated samples use Sherpa’s own internal parton shower and fragmentation methods, as well as the Sherpa default underlying-event tune [47]. The standard ATLAS underlying-event tune, AUET2 [48], is used for all other samples with the exception of the Powheg+Pythia samples, which use the Perugia2011C [49] tune.

**Fig. 1:**
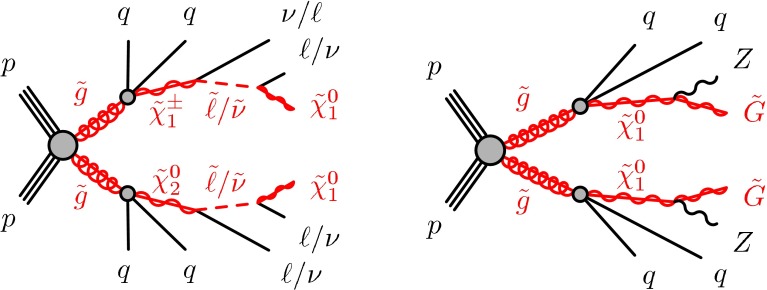
Decay topologies for example signal processes. A simplified model involving gluino pair production, with the gluinos following two-step decays via sleptons to neutralino LSPs is shown on the *left*. The diagram on the *right* shows a GGM decay mode, where gluinos decay via neutralinos to gravitino LSPs

The signal models considered include simplified models and a GGM supersymmetry-breaking model. In the simplified models, squarks and gluinos are directly pair-produced, and these subsequently decay to the LSP via two sets of intermediate particles. The squarks and gluinos decay with equal probability to the next-to-lightest neutralino or the lightest chargino, where the neutralino and chargino are mass-degenerate and have masses taken to be the average of the squark or gluino mass and the LSP mass. The intermediate chargino or neutralino then decays via sleptons (or sneutrinos) to two leptons of the same flavour and the lightest neutralino, which is assumed to be the LSP in these models. Here, the sleptons and sneutrinos are mass-degenerate and have masses taken to be the average of the chargino or neutralino and LSP masses. An example of one such process, $$pp\rightarrow \tilde{g}\tilde{g}\rightarrow (q\bar{q}\tilde{\chi }_{2}^{0})(q\bar{q}\tilde{\chi }_{1}^{\pm }), \tilde{\chi }_{2}^{0}\rightarrow \ell ^{+}\ell ^{-}\tilde{\chi }_{1}^{0},$$$$\tilde{\chi }_{1}^{\pm }\rightarrow \ell ^{\pm }\nu \tilde{\chi }_{1}^{0}$$ is illustrated on the left in Fig. 1, where $$\ell =e,\mu ,\tau $$ with equal branching fractions for each lepton flavour. The dilepton mass distribution for leptons produced from the $$\tilde{\chi }_{2}^{0}$$ in these models is a rising distribution that terminates at a kinematic endpoint, whose value is given by $$m_{\mathrm {max}}$$$$\approx m(\tilde{\chi }_{2}^{0})-m(\tilde{\chi }_{1}^{0}) = 1/2(m(\tilde{g}/\tilde{q})-m(\tilde{\chi }_{1}^{0}))$$. Therefore, signal models with small values of $$\Delta m = m(\tilde{g}/\tilde{q})-m(\tilde{\chi }_{1}^{0})$$ produce events with small dilepton masses; those with large $$\Delta m$$ produce events with large dilepton mass.

For the model involving squark pair production, the left-handed partners of the *u*, *d*, *c* and *s* quarks have the same mass. The right-handed squarks and the partners of the *b* and *t* quarks are decoupled. For the gluino-pair model, an effective three-body decay for $\tilde{g}\rightarrow q \bar{q} \tilde{\chi }_{1}^{0}$ is used, with equal branching fractions for $q=u,d,c,s$. Exclusion limits on these models are set based on the squark or gluino mass and the LSP mass, with all sparticles not directly involved in the considered decay chains effectively being decoupled.

In the general gauge mediation models, the gravitino is the LSP and the next-to-lightest SUSY particle (NLSP) is a higgsino-like neutralino. The higgsino mass parameter, $\mu$, and the gluino mass are free parameters. The U(1) and SU(2) gaugino mass parameters, $M_{1}$ and $M_{2}$, are fixed to be 1 TeV, and the masses of all other sparticles are set at $\sim$1.5 TeV. In addition, $\mu$ is set to be positive to make $\tilde{\chi }^{0}_{1} \rightarrow Z \tilde{G}$ the dominant NLSP decay. The branching fraction for $\tilde{\chi }^{0}_{1} \rightarrow Z \tilde{G}$ varies with $\tan \beta$, the ratio of the vacuum expectation value for the two Higgs doublets, and so two different values of $\tan \beta$ are used. At $\tan \beta =1.5$, the branching fraction for $\tilde{\chi }^{0}_{1} \rightarrow Z \tilde{G}$ is large (about 97 %) [50], whereas setting $\tan \beta =30$ results in a considerable contribution (up to 40 %) from $\tilde{\chi }^{0}_{1} \rightarrow h \tilde{G}$. In these models, *h* is the lightest CP-even SUSY Higgs boson, with $m_{h}=126$ GeV and SM-like branching fractions. The dominant SUSY-particle production mode in these scenarios is the strong production of gluino pairs, which subsequently decay to the LSP via several intermediate particles. An example decay mode is shown in the diagram on the right in Fig. 1. The gravitino mass is set to be sufficiently small such that the NLSP decays are prompt. The decay length $c\tau _{\mathrm{NLSP}}$ (where $\tau _{\mathrm{NLSP}}$ is the lifetime of the NLSP) can vary depending on $\mu$, and is longest at $\mu =120$ GeV, where it is 2 mm, decreasing to $c\tau _{\mathrm{NLSP}}<0.1$ mm for $\mu \ge 150\,{\mathrm {GeV}}$. The finite NLSP lifetime is taken into account in the MC signal acceptance and efficiency determination.

All simplified models are produced using MadGraph5 1.3.33 with the CTEQ6L1 PDF set, interfaced with Pythia 6.426. The scale parameter for MLM matching [51] is set at a quarter of the mass of the lightest strongly produced sparticle in the matrix element. The SUSY mass spectra, gluino branching fractions and the gluino decay width for the GGM scenarios are calculated using Suspect 2.41 [52] and Sdecay 1.3 [53]. The GGM signal samples are generated using Pythia 6.423 with the MRST2007 LO$^{*}$ [54] PDF set. The underlying event is modelled using the AUET2 tune for all signal samples. Signals are normalised to cross sections calculated at next-to-leading order (NLO) in $\alpha _{s}$, including the resummation of soft gluon emission at next-to-leading-logarithmic accuracy (NLO + NLL) [55–59].

A full ATLAS detector simulation [60] using GEANT4 [61] is performed for most of the SM background MC samples. The signal and remaining SM MC samples use a fast simulation [62], which employs a combination of a parameterisation of the response of the ATLAS electromagnetic and hadronic calorimeters and GEANT4. To simulate the effect of multiple *pp* interactions occurring during the same (in-time) or a nearby (out-of-time) bunch-crossing, called pile-up, minimum-bias interactions are generated and overlaid on top of the hard-scattering process. These are produced using Pythia8 with the A2 tune [63]. MC-to-data corrections are made to simulated samples to account for small differences in lepton identification and reconstruction efficiencies, and the efficiency and misidentification rate associated with the algorithm used to distinguish jets containing *b*-hadrons.

**Table 1:** Simulated background event samples used in this analysis with the corresponding generator, cross-section order in $\alpha _{\mathrm{s}}$ used to normalise the event yield, underlying-event tune and PDF set

Physics process	Generator	Parton shower	Cross section	Tune	PDF set
$Z/\gamma ^{*}(\rightarrow \ell \ell )$ + jets	Sherpa 1.4.1	Sherpa 1.4.1	NNLO [29, 30]	Sherpa default	NLO CT10 [31]
$t\bar{t}$	Powheg-Box r2129	Pythia 6.426	NNLO + NNLL [32, 33]	Perugia2011C	NLO CT10
Single-top (*Wt*)	Powheg-Box r1556	Pythia 6.426	Approx. NNLO [34, 35]	Perugia2011C	NLO CT10
$t+Z$	MadGraph5 1.3.28	Pythia 6.426	LO	AUET2	CTEQ6L1 [36]
$t\bar{t}+W$ and $t\bar{t}+Z$	MadGraph5 1.3.28	Pythia 6.426	NLO [37, 38]	AUET2	CTEQ6L1
$t\bar{t}+WW$	MadGraph5 1.3.28	Pythia 8.165	LO	AUET2	CTEQ6L1
*WW*, *WZ* and *ZZ*	powheg-box r1508	Pythia 8.163	NLO [39, 40]	AUET2	NLO CT10

## Physics object identification and selection

Electron candidates are reconstructed using energy clusters in the electromagnetic calorimeter matched to ID tracks. Electrons used in this analysis are assigned either “baseline” or “signal” status. Baseline electrons are required to have transverse energy $E_{\mathrm{T}}>10$ GeV, satisfy the “medium” criteria described in Ref. [64] and reside within $|\eta |<2.47$ and not in the range $1.37<|\eta |<1.52$. Signal electrons are further required to be consistent with the primary vertex and isolated with respect to other objects in the event, with a ${p_{\mathrm{T}}}$-dependent isolation requirement. The primary vertex is defined as the reconstructed vertex with the highest $\sum p_{\mathrm{T}}^2$, where the summation includes all particle tracks with ${p_{\mathrm{T}}} >400$ $\mathrm {MeV}$ associated with a given reconstructed vertex. Signal electrons with $E_{\mathrm{T}}<25$ GeV must additionally satisfy the more stringent shower shape, track quality and matching requirements of the “tight” selection criteria in Ref. [64]. For electrons with $E_{\mathrm{T}}<25$ GeV ($\ge$25 GeV), the sum of the transverse momenta of all charged-particle tracks with ${p_{\mathrm{T}}} >400$ $\mathrm {MeV}$ associated with the primary vertex, excluding the electron track, within $\Delta R = 0.3$ (0.2) surrounding the electron must be less than 16 % (10 %) of the electron ${p_{\mathrm{T}}}$. Electrons with $E_{\mathrm{T}}<25$ GeV must reside within a distance $|z_{0}\sin \theta | < 0.4$ mm of the primary vertex along the direction of the beamline^2^. The significance of the transverse-plane distance of closest approach of the electron to the primary vertex must be $|d_{0}/\sigma _{d_{0}}|<5$. For electrons with $E_{\mathrm{T}} \ge 25$ GeV, $|z_{0}|$ is required to be $<2$ mm and $|d_{0}|<1$ mm.

Baseline muons are reconstructed from either ID tracks matched to a muon segment in the muon spectrometer or combined tracks formed both from the ID and muon spectrometer [65]. They are required to be of good quality, as described in Ref. [66], and to satisfy $p_{\mathrm{T}}>10$ GeV and $|\eta |<2.4$. Signal muons are further required to be isolated, with the scalar sum of the ${p_{\mathrm{T}}}$ of charged particle tracks associated with the primary vertex, excluding the muon track, within a cone of size $\Delta R<0.3$ surrounding the muon being less than 12 % of the muon ${p_{\mathrm{T}}}$ for muons with $p_{\mathrm{T}}<25$ GeV. For muons with $p_{\mathrm{T}} \ge 25$ GeV, the scalar sum of the ${p_{\mathrm{T}}}$ of charged-particle tracks associated with the primary vertex, excluding the muon track, within $\Delta R<0.2$ surrounding the muon must be less than 1.8 GeV. Signal muons with $p_{\mathrm{T}}<25$ GeV must also have $|z_{0}\sin \theta | \le 1$ mm and $|d_{0}/\sigma _{d_{0}}|<3$. For the leptons selected by this analysis, the $d_{0}$ requirement is typically several times less restrictive than the $|d_{0}/\sigma _{d_{0}}|$ requirement.

Jets are reconstructed from topological clusters in the calorimeter using the anti-$k_{t}$ algorithm [67] with a distance parameter of 0.4. Each cluster is categorised as being electromagnetic or hadronic in origin according to its shape [68], so as to account for the differing calorimeter response for electrons/photons and hadrons. A cluster-level correction is then applied to electromagnetic and hadronic energy deposits using correction factors derived from both MC simulation and data. Jets are corrected for expected pile-up contributions [69] and further calibrated to account for the calorimeter response with respect to the true jet energy [70, 71]. A small residual correction is applied to the jets in data to account for differences between response in data and MC simulation. Baseline jets are selected with $p_{\mathrm{T}}>20$ GeV. Events in which these jets do not pass specific jet quality requirements are rejected so as to remove events affected by detector noise and non-collision backgrounds [72, 73]. Signal jets are required to satisfy $p_{\mathrm{T}}>35$ GeV and $|\eta |<2.5$. To reduce the impact of jets from pileup to a negligible level, jets with $p_{\mathrm{T}}<50$ GeV within $|\eta |<2.4$ are further required to have a jet vertex fraction $|\mathrm {JVF}|> 0.25$. Here the JVF is the ${p_{\mathrm{T}}}$-weighted fraction of tracks matched to the jet that are associated with the primary vertex [74], with jets without any associated tracks being assigned $\text {JVF}=-1$.

The MV1 neural network algorithm [75] identifies jets containing *b*-hadrons using the impact parameters of associated tracks and any reconstructed secondary vertices. For this analysis, the working point corresponding to a 60 % efficiency for tagging *b*-jets in simulated $t\bar{t}$ events is used, resulting in a charm quark rejection factor of approximately 8 and a light quark/gluon jet rejection factor of about 600. To ensure that each physics object is counted only once, an overlap removal procedure is applied. If any two baseline electrons reside within $\Delta R=0.05$ of one another, the electron with lower $E_{\mathrm{T}}$ is discarded. Following this, any baseline jets within $\Delta R=0.2$ of a baseline electron are removed. After this, any baseline electron or muon residing within $\Delta R=0.4$ of a remaining baseline jet is discarded. Finally, to remove electrons originating from muon bremsstrahlung, any baseline electron within $\Delta R=0.01$ of any remaining baseline muon is removed from the event.

The ${E_{\mathrm{T}}^{\mathrm{miss}}}$ is defined as the magnitude of the vector sum of the transverse momenta of all photons, electrons, muons, baseline jets and an additional “soft term” [76]. The soft term includes clusters of energy in the calorimeter not associated with any calibrated object, which are corrected for material effects and the non-compensating nature of the calorimeter. Reconstructed photons used in the ${E_{\mathrm{T}}^{\mathrm{miss}}}$ calculation are required to satisfy the “tight” requirements of Ref. [77].

## Event selection

Events selected for this analysis must have at least five tracks with $p_{\mathrm{T}}>400$ MeV associated with the primary vertex. Any event containing a baseline muon with $|z_{0} \sin \theta |>0.2$ mm or $|d_{0}|>1$ mm is rejected, to remove cosmic-ray events. To reject events with fake ${E_{\mathrm{T}}^{\mathrm{miss}}}$, those containing poorly measured muon candidates, characterised by large uncertainties on the measured momentum, are also removed. If the invariant mass of the two leading leptons in the event is less than 15 GeV the event is vetoed to suppress low-mass particle decays and Drell–Yan production.

Events are required to contain at least two signal leptons (electrons or muons). If more than two signal leptons are present, the two with the largest values of ${p_{\mathrm{T}}}$ are selected. These leptons must pass one of the leptonic triggers, with the two leading leptons being matched, within $\Delta R<0.15$, to the online trigger objects that triggered the event in the case of the dilepton triggers. For events selected by a single-lepton trigger, one of the two leading leptons must be matched to the online trigger object in the same way. The leading lepton in the event must have $p_{\mathrm{T}}>25$ GeV and the sub-leading lepton is required to have $p_{\mathrm{T}}>10$–14 ${\mathrm {GeV}}$, depending on the ${p_{\mathrm{T}}}$ theshold of the trigger selecting the event. For the off-*Z* analysis, the sub-leading lepton $p_{\mathrm{T}}$ threshold is increased to 20 GeV. This is done to improve the accuracy of the method for estimating flavour-symmetric backgrounds, discussed in Sect. 6.2, in events with small dilepton invariant mass. For the same reason, the $m_{\ell \ell }$ threshold is also raised to 20 GeV in this search channel. The two leading leptons must be oppositely charged, with the signal selection requiring that these be same-flavour (SF) lepton pairs. The different-flavour (DF) channel is also exploited to estimate certain backgrounds, such as that due to $t\bar{t}$ production. All events are further required to contain at least two signal jets, since this is the minimum expected jet multiplicity for the signal models considered in this analysis.

Three types of region are used in the analysis. Control regions (CRs) are used to constrain the SM backgrounds. These backgrounds, estimated in the CRs, are first extrapolated to the validation regions (VRs) as a cross check and then to the signal regions (SRs), where an excess over the expected background is searched for.

GGM scenarios are the target of the on-*Z* search, where the $\tilde{G}$ from $\tilde{\chi }^{0}_{1} \rightarrow (Z/h)+ \tilde{G}$ decays is expected to result in ${E_{\mathrm{T}}^{\mathrm{miss}}}$. The *Z* boson mass window used for this search is $81<m_{\ell \ell }<101$ GeV. To isolate GGM signals with high gluino mass and high jet activity the on-*Z* SR, SR-Z, is defined using requirements on ${E_{\mathrm{T}}^{\mathrm{miss}}}$ and $H_{\mathrm{T}} = \sum _i p_{\mathrm{T}}^{\mathrm{jet},i} + p_{\mathrm{T}}^{\mathrm{lepton},1} + p_{\mathrm{T}}^{\mathrm{lepton},2}$, where ${H_{\mathrm{T}}}$ includes all signal jets and the two leading leptons. Since *b*-jets are often, but not always, expected in GGM decay chains, no requirement is placed on *b*-tagged jet multiplicity. Dedicated CRs are defined in order to estimate the contribution of various SM backgrounds to the SR. These regions are constructed with selection criteria similar to those of the SR, differing either in mll or MET ranges, or in lepton flavour requirements. A comprehensive discussion of the various methods used to perform these estimates follows in Sect. 6. For the SR and CRs, detailed in Table 2, a further requirement on the azimuthal opening angle between each of the leading two jets and the ${E_{\mathrm{T}}^{\mathrm{miss}}}$ ($\Delta \phi (\text {jet}_{1,2},{E_{\mathrm{T}}^{\mathrm{miss}}})$) is introduced to reject events with jet mismeasurements contributing to large fake ${E_{\mathrm{T}}^{\mathrm{miss}}}$. This requirement is applied in the SR and two CRs used in the on-*Z* search, all of which have high ${E_{\mathrm{T}}^{\mathrm{miss}}}$ and $H_{\mathrm{T}}$ thresholds, at 225 and 600 GeV, respectively. Additional VRs are defined at lower ${E_{\mathrm{T}}^{\mathrm{miss}}}$ and $H_{\mathrm{T}}$ to cross-check the SM background estimation methods. These are also sumarised in Table 2. The SR selection results in an acceptance times efficiency of 2–4 %, including leptonic *Z* branching fractions, for GGM signal models with $\mu >400\,{\mathrm {GeV}}$.

In the off-*Z* analysis, a search is performed in the *Z* boson sidebands. The *Z* boson mass window vetoed here is larger than that selected in the on-*Z* analysis ($m_{\ell \ell } \notin [80,110]$ GeV) to maximise *Z* boson rejection. An asymmetric window is chosen to improve the suppression of boosted $Z\rightarrow \mu \mu$ events with muons whose momenta are overestimated, leading to large ${E_{\mathrm{T}}^{\mathrm{miss}}}$. In this search, four SRs are defined by requirements on jet multiplicity, *b*-tagged jet multiplicity, and ${E_{\mathrm{T}}^{\mathrm{miss}}}$. The SR requirements are optimised for the simplified models of pair production of squarks (requiring at least two jets) and gluinos (requiring at least four jets) discussed in Sect. 3. Two SRs with a *b*-veto provide the best sensitivity in the simplified models considered here, since the signal *b*-jet content is lower than that of the dominant $t\bar{t}$ background. Orthogonal SRs with a requirement of at least one *b*-tagged jet target other signal models not explicitly considered here, such as those with bottom squarks that are lighter than the other squark flavours. For these four SRs, the requirement ${E_{\mathrm{T}}^{\mathrm{miss}}} >200\,{\mathrm {GeV}}$ is imposed. In addition, one signal region with requirements similar to those used in the CMS search [24] is defined (SR-loose). These SRs and their respective CRs, which have the same jet and ${E_{\mathrm{T}}^{\mathrm{miss}}}$ requirements, but select different $m_{\ell \ell }$ ranges or lepton flavour combinations, are defined in Table 3.

The most sensitive off-*Z* SR for the squark-pair (gluino-pair) model is SR-2j-bveto (SR-4j-bveto). Because the value of the ${m_{\ell \ell }}$ kinematic endpoint depends on unknown model parameters, the analysis is performed over multiple ${m_{\ell \ell }}$ ranges for these two SRs. The dilepton mass windows considered for the SR-2j-bveto and SR-4j-bveto regions are presented in Sect. 9. For the combined $ee+\mu \mu$ channels, the typical signal acceptance times efficiency values for the squark-pair (gluino-pair) model in the SR-2j-bveto (SR-4j-bveto) region are 0.1–10 % (0.1–8 %) over the full dilepton mass range.

The on-*Z* and off-*Z* searches are optimised for different signal models and as such are defined with orthogonal SRs. Given the different signatures probed, there are cases where the CR of one search may overlap with the SR of the other. Data events that fall in the off-*Z* SRs can comprise up to 60 % of the top CR for the on-*Z* analysis (CRT, defined in Table 2). Data events in SR-Z comprise up to 36 % of the events in the CRs with $80<{m_{\ell \ell }}<110\,{\mathrm {GeV}}$ that are used to normalise the ${Z+\text {jets}}$ background in the off-*Z* analysis, but the potential impact on the background prediction is small because the ${Z+\text {jets}}$ contribution is a small fraction of the total background. For the following analysis, each search assumes only signal contamination from the specific signal model they are probing.

**Table 2:** Overview of all signal, control and validation regions used in the on-*Z* search. More details are given in the text. The ${E_{\mathrm{T}}^{\mathrm{miss}}}$ significance and the soft-term fraction $f_{\mathrm{ST}}$ needed in the seed regions for the jet smearing method are defined in Sect. 6.1. The flavour combination of the dilepton pair is denoted as either “SF” for same-flavour or “DF” for different flavour

On-*Z* region	${E_{\mathrm{T}}^{\mathrm{miss}}}$ (${\mathrm {GeV}}$)	${H_{\mathrm{T}}}$ (${\mathrm {GeV}}$)	$n_{\mathrm{jets}}$	$m_{\ell \ell }$ (${\mathrm {GeV}}$)	SF/DF	${E_{\mathrm{T}}^{\mathrm{miss}}}$ sig. $(\sqrt{{\mathrm {GeV}}})$	$f_{\mathrm{ST}}$	$\Delta \phi ({\mathrm{jet}}_{12},{E_{\mathrm{T}}^{\mathrm{miss}}})$
Signal regions								
SR-Z	$>$225	$>$600	$\ge$2	$81 < m_{\ell \ell } < 101$	SF	–	–	$>$0.4
Control regions								
Seed region	–	$> 600$	$\ge$2	$81 < m_{\ell \ell } < 101$	SF	$<$0.9	$<$0.6	–
CRe$\mu$	$>$225	$>$600	$\ge$2	$81 < m_{\ell \ell } < 101$	DF	–	–	$>$0.4
CRT	$>$225	$>$600	$\ge$2	$m_{\ell \ell } \notin [81,\,101]$	SF	–	–	$>$0.4
Validation regions								
VRZ	$<$150	$>$600	$\ge$2	$81 < m_{\ell \ell } < 101$	SF	–	–	–
VRT	150–225	$>$500	$\ge$2	$m_{\ell \ell } \notin [81,\,101]$	SF	–	–	$>$0.4
VRTZ	150–225	$>$500	$\ge$2	$81 < m_{\ell \ell } < 101$	SF	–	–	$>$0.4

**Table 3:** Overview of all signal, control and validation regions used in the off-*Z* analysis. For SR-loose, events with two jets (at least three jets) are required to satisfy ${E_{\mathrm{T}}^{\mathrm{miss}}}$ $>$ 150 (100) ${\mathrm {GeV}}$. Further details are the same as in Table 2

Off-*Z* region	${E_{\mathrm{T}}^{\mathrm{miss}}}$ (${\mathrm {GeV}}$)	$n_{\mathrm{jets}}$	$n_{\mathrm{b-jets}}$	$m_{\ell \ell }$ (${\mathrm {GeV}}$)	SF/DF
Signal regions					
SR-2j-bveto	$>$200	$\ge$2	= 0	$m_{\ell \ell } \notin [80,\,110]$	SF
SR-2j-btag	$>$200	$\ge$2	$\ge$1	$m_{\ell \ell } \notin [80,\,110]$	SF
SR-4j-bveto	$>$200	$\ge$4	= 0	$m_{\ell \ell } \notin [80,\,110]$	SF
SR-4j-btag	$>$200	$\ge$4	$\ge$1	$m_{\ell \ell } \notin [80,\,110]$	SF
SR-loose	$>$(150, 100)	$(2, \ge 3)$	–	$m_{\ell \ell } \notin [80,\,110]$	SF
Control regions					
CRZ-2j-bveto	$>$200	$\ge$2	= 0	$80 < m_{\ell \ell } < 110$	SF
CRZ-2j-btag	$>$200	$\ge$2	$\ge$1	$80 < m_{\ell \ell } < 110$	SF
CRZ-4j-bveto	$>$200	$\ge$4	= 0	$80 < m_{\ell \ell } < 110$	SF
CRZ-4j-btag	$>$200	$\ge$4	$\ge$1	$80 < m_{\ell \ell } < 110$	SF
CRZ-loose	$>$(150, 100)	$(2,\ge 3)$	–	$80 < m_{\ell \ell } < 110$	SF
CRT-2j-bveto	$>$200	$\ge$2	= 0	$m_{\ell \ell } \notin [80,\,110]$	DF
CRT-2j-btag	$>$200	$\ge$2	$\ge$1	$m_{\ell \ell } \notin [80,\,110]$	DF
CRT-4j-bveto	$>$200	$\ge$4	= 0	$m_{\ell \ell } \notin [80,\,110]$	DF
CRT-4j-btag	$>$200	$\ge$4	$\ge$1	$m_{\ell \ell } \notin [80,\,110]$	DF
CRT-loose	$>$(150, 100)	$(2,\ge 3)$	–	$m_{\ell \ell } \notin [80,\,110]$	DF
Validation regions					
VR-offZ	100–150	= 2	–	$m_{\ell \ell } \notin [80,\,110]$	SF

## Background estimation

The dominant background processes in the signal regions, and those that are expected to be most difficult to model using MC simulation, are estimated using data-driven techniques. With SRs defined at large ${E_{\mathrm{T}}^{\mathrm{miss}}}$, any contribution from ${Z/\gamma ^{*}+\text {jets}}$ will be a consequence of artificially high ${E_{\mathrm{T}}^{\mathrm{miss}}}$ in the event due to, for example, jet mismeasurements. This background must be carefully estimated, particularly in the on-*Z* search, since the peaking ${Z/\gamma ^{*}+\text {jets}}$ background can mimic the signal. This background is expected to constitute, in general, less than 10 % of the total background in the off-*Z* SRs and have a negligible contribution to SR-Z.

In both the off-*Z* and on-*Z* signal regions, the dominant backgrounds come from so-called “flavour-symmetric” processes, where the dileptonic branching fractions to *ee*, $\mu \mu$ and $e\mu$ have a 1:1:2 ratio such that the same-flavour contributions can be estimated using information from the different-flavour contribution. This group of backgrounds is dominated by $t\bar{t}$ and also includes *WW*, single top (*Wt*) and $Z\rightarrow \tau \tau$ production, and makes up $\sim$60 % ($\sim$ 90 %) of the predicted background in the on-*Z* (off-*Z*) SRs.

Diboson backgrounds with real *Z* boson production, while small in the off-*Z* regions, contribute up to 25 % of the total background in the on-*Z* regions. These backgrounds are estimated using MC simulation, as are “rare top” backgrounds, including $t\bar{t}+W(W)/Z$ (i.e. $t\bar{t}+W$, $t\bar{t}+Z$ and $t\bar{t}+WW$) and $t+Z$ processes. All backgrounds that are estimated from MC simulation are subject to carefully assessed theoretical and experimental uncertainties.

Other processes, including those that might be present due to mis-reconstructed jets entering as leptons, can contribute up to 10 % (6 %) in the on-*Z* (off-*Z*) SRs. The background estimation techniques followed in the on-*Z* and off-*Z* searches are similar, with a few well-motivated exceptions.

### Estimation of the $Z/\gamma ^{*}+$ jets background

#### $Z/\gamma ^{*}+$ jets background in the off-*Z* search

In the off-*Z* signal regions, the background from ${Z/\gamma ^{*}+\text {jets}}$ is due to off-shell *Z* bosons and photons, or to on-shell *Z* bosons with lepton momenta that are mismeasured. The region with dilepton mass in the range $80<m_{\ell \ell }<110$ GeV is not considered as a search region. To estimate the contribution from ${Z/\gamma ^{*}+\text {jets}}$ outside of this range, dilepton mass shape templates are derived from ${Z/\gamma ^{*}+\text {jets}}$ MC events. These shape templates are normalised to data in control regions with the same selection as the corresponding signal regions, but with the requirement on $m_{\ell \ell }$ inverted to $80<m_{\ell \ell }<110$ GeV, to select a sample enriched in ${Z/\gamma ^{*}+\text {jets}}$ events. These CRs are defined in Table 3.

#### $Z/\gamma ^{*}+$ jets background in the on-*Z* search

The assessment of the peaking background due to ${Z/\gamma ^{*}+\text {jets}}$ in the on-*Z* signal regions requires careful consideration. The events that populate the signal regions result from mismeasurements of physics objects where, for example, one of the final-state jets has its energy underestimated, resulting in an overestimate of the total ${E_{\mathrm{T}}^{\mathrm{miss}}}$ in the event. Due to the difficulties of modelling instrumental ${E_{\mathrm{T}}^{\mathrm{miss}}}$ in simulation, MC events are not relied upon alone for the estimation of the ${Z/\gamma ^{*}+\text {jets}}$ background. A data-driven technique is used as the nominal method for estimating this background. This technique confirms the expectation from MC simulation that the $Z+\mathrm {jets}$ background is negligible in the SR.

The primary method used to model the ${Z/\gamma ^{*}+\text {jets}}$ background in SR-Z is the so-called “jet smearing” method, which is described in detail in Ref. [78]. This involves defining a region with ${Z/\gamma ^{*}+\text {jets}}$ events containing well-measured jets (at low ${E_{\mathrm{T}}^{\mathrm{miss}}}$), known as the “seed” region. The jets in these events are then smeared using functions that describe the detector’s jet ${p_{\mathrm{T}}}$ response and $\phi$ resolution as a function of jet ${p_{\mathrm{T}}}$, creating a set of pseudo-data events. The jet-smearing method provides an estimate for the contribution from events containing both fake ${E_{\mathrm{T}}^{\mathrm{miss}}}$, from object mismeasurements, and real ${E_{\mathrm{T}}^{\mathrm{miss}}}$, from neutrinos in heavy-flavour quark decays, by using different response functions for light-flavour and *b*-tagged jets. The response function is measured by comparing generator-level jet ${p_{\mathrm{T}}}$ to reconstructed jet ${p_{\mathrm{T}}}$ in Pythia8 dijet MC events, generated using the CT10 NLO PDF set. This function is then tuned to data, based on a dijet balance analysis in which the ${p_{\mathrm{T}}}$ asymmetry is used to constrain the width of the Gaussian core. The non-Gaussian tails of the response function are corrected based on $\ge$3-jet events in data, selected such that the ${E_{\mathrm{T}}^{\mathrm{miss}}}$ in each event points either towards, or in the opposite direction to one of the jets. This ensures that one of the jets is clearly associated with the ${E_{\mathrm{T}}^{\mathrm{miss}}}$, and the jet response can then be described in terms of the ${E_{\mathrm{T}}^{\mathrm{miss}}}$ and reconstructed jet ${p_{\mathrm{T}}}$. This procedure results in a good estimate of the overall jet response.

In order to calculate the $${E_{\mathrm{T}}^{\mathrm{miss}}} $$ distribution of the pseudo-data, the $${E_{\mathrm{T}}^{\mathrm{miss}}} $$ is recalculated using the new (smeared) jet $${p_{\mathrm{T}}} $$ and $$\phi $$. The distribution of pseudo-data events is then normalised to data in the low-$${E_{\mathrm{T}}^{\mathrm{miss}}} $$ region (10 $$<$$$${E_{\mathrm{T}}^{\mathrm{miss}}} $$$$<$$ 50 GeV) of a validation region, denoted VRZ, after the requirement of $$\Delta \phi (\text {jet}_{1,2}, {E_{\mathrm{T}}^{\mathrm{miss}}})>0.4$$. This is defined in Table 2 and is designed to be representative of the signal region but at lower $${E_{\mathrm{T}}^{\mathrm{miss}}} $$, where the contamination for relevant GGM signal models is expected to be less than 1 %.

The seed region must contain events with topologies similar to those expected in the signal region. To ensure that this is the case, the ${H_{\mathrm{T}}}$ and jet multiplicity requirements applied to the seed region remain the same as in the signal region, while the ${E_{\mathrm{T}}^{\mathrm{miss}}}$ threshold of 225 GeV is removed, as shown in Table 2. Although the seed events should have little to no ${E_{\mathrm{T}}^{\mathrm{miss}}}$, enforcing a direct upper limit on ${E_{\mathrm{T}}^{\mathrm{miss}}}$ can introduce a bias in the jet ${p_{\mathrm{T}}}$ distribution in the seed region compared with the signal region. To avoid this, a requirement on the ${E_{\mathrm{T}}^{\mathrm{miss}}}$  significance, defined as:

1 $$\begin{aligned} {E_{\mathrm{T}}^{\mathrm{miss}}} \, \text {sig.} = \frac{{E_{\mathrm{T}}^{\mathrm{miss}}}}{\sqrt{\sum E_{\mathrm{T}}^{\mathrm{jet}} + \sum E_{\mathrm{T}}^{\mathrm{soft}}}} , \end{aligned}$$

is used in the seed region. Here $\sum E_{\mathrm{T}}^{\mathrm{jet}}$ and $\sum E_{\mathrm{T}}^{\mathrm{soft}}$ are the summed $E_{\mathrm{T}}$ from the baseline jets and the low-energy calorimeter deposits not associated with final-state physics objects, respectively. Placing a requirement on this variable does not produce a shape difference between jet ${p_{\mathrm{T}}}$ distributions in the seed and signal regions, while effectively selecting well-balanced ${Z/\gamma ^{*}+\text {jets}}$ events in the seed region. This requirement is also found to result in no event overlap between the seed region and SR-Z.

In the seed region an additional requirement is placed on the soft-term fraction, $f_{\mathrm{ST}}$, defined as the fraction of the total ${E_{\mathrm{T}}^{\mathrm{miss}}}$ in an event originating from calorimeter energy deposits not associated with a calibrated lepton or jet ($f_{\mathrm{ST}} = \sum E_{\mathrm{T}}^{\mathrm{miss, Soft}} / {E_{\mathrm{T}}^{\mathrm{miss}}}$), to select events with small $f_{\mathrm{ST}}$. This is useful because events with large values of fake ${E_{\mathrm{T}}^{\mathrm{miss}}}$ tend to have low soft-term fractions ($f_{\mathrm{ST}}<0.6$).

The requirements on the ${E_{\mathrm{T}}^{\mathrm{miss}}}$ significance and $f_{\mathrm{ST}}$ are initially optimised by applying the jet smearing method to ${Z/\gamma ^{*}+\text {jets}}$ MC events and testing the agreement in the ${E_{\mathrm{T}}^{\mathrm{miss}}}$ spectrum between direct and smeared MC events in the VRZ. This closure test is performed using the response function derived from MC simulation.

The ${Z/\gamma ^{*}+\text {jets}}$ background predominantly comes from events where a single jet is grossly mismeasured, since the mismeasurement of additional jets is unlikely, and can lead to smearing that reduces the total ${E_{\mathrm{T}}^{\mathrm{miss}}}$. The requirement on the opening angle in $\phi$ between either of the leading two jets and the ${E_{\mathrm{T}}^{\mathrm{miss}}}$, $\Delta \phi (\text {jet}_{1,2},{E_{\mathrm{T}}^{\mathrm{miss}}})>0.4$, strongly suppresses this background. The estimate of the ${Z/\gamma ^{*}+\text {jets}}$ background is performed both with and without this requirement, in order to aid in the interpretation of the results in the SR, as described in Sect. 8. The optimisation of the ${E_{\mathrm{T}}^{\mathrm{miss}}}$ significance and $f_{\mathrm{ST}}$ requirements are performed separately with and without the requirement, although the optimal values are not found to differ significantly.

**Fig. 2:**
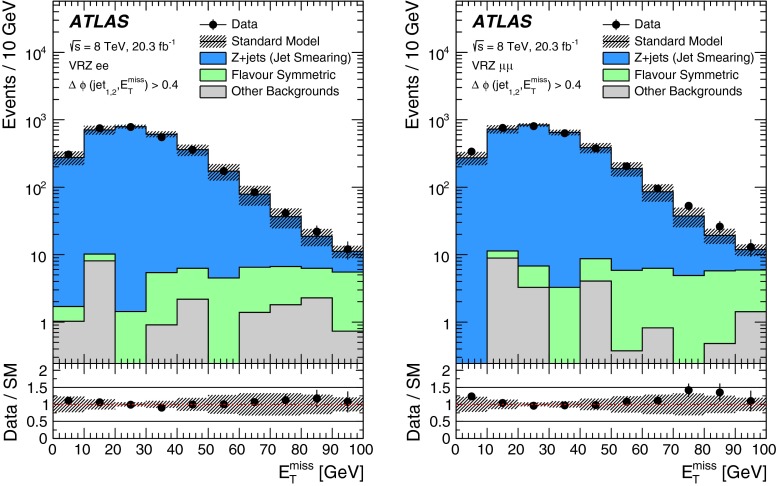
Distribution of ${E_{\mathrm{T}}^{\mathrm{miss}}}$ in the electron (*left*) and muon (*right*) channel in VRZ of the on-*Z* analysis following the requirement of $\Delta \phi (\text {jet}_{1,2},{E_{\mathrm{T}}^{\mathrm{miss}}})>0.4$. Here the ${Z/\gamma ^{*}+\text {jets}}$ background (*solid blue*) is modelled using ${p_{\mathrm{T}}}$- and $\phi$-smeared pseudo-data events. The *hatched uncertainty band* includes the statistical uncertainty on the simulated event samples and the systematic uncertainty on the jet-smearing estimate due to the jet response function and the seed selection. The backgrounds due to *WZ*, *ZZ* or rare top processes, as well as from lepton fakes, are included under “Other Backgrounds”

The jet smearing method using the data-corrected jet response function is validated in VRZ, comparing smeared pseudo-data to data. The resulting $${E_{\mathrm{T}}^{\mathrm{miss}}} $$ distributions show agreement within uncertainties assessed based on varying the response function and the $${E_{\mathrm{T}}^{\mathrm{miss}}} $$ significance requirement in the seed region. The $${E_{\mathrm{T}}^{\mathrm{miss}}} $$ distribution in VRZ, with the additional requirement $$\Delta \phi (\text {jet}_{1,2},$$$${E_{\mathrm{T}}^{\mathrm{miss}}})>0.4$$, is shown in Fig. 2. Here the $${E_{\mathrm{T}}^{\mathrm{miss}}} $$ range extends only up to 100 $${\mathrm {GeV}}$$, since $$t\bar{t}$$ events begin to dominate at higher $${E_{\mathrm{T}}^{\mathrm{miss}}} $$ values. The pseudo-data to data agreement in VRZ motivates the final determination of the $${E_{\mathrm{T}}^{\mathrm{miss}}} $$ significance requirement used for the seed region ($${E_{\mathrm{T}}^{\mathrm{miss}}}\ \text {sig.}<0.9$$). Backgrounds containing real $${E_{\mathrm{T}}^{\mathrm{miss}}} $$, including $$t\bar{t}$$ and diboson production, are taken from MC simulation for this check. The chosen values are detailed in Table 2 with a summary of the kinematic requirements imposed on the seed and *Z* validation region. Extrapolating the jet smearing estimate to the signal regions yields the results detailed in Table 4. The data-driven estimate is compatible with the MC expectation that the $$Z+\mathrm {jets}$$ background contributes significantly less than one event in SR-Z.

**Table 4:** Number of ${Z/\gamma ^{*}+\text {jets}}$ background events estimated in the on-*Z* signal region (SR-Z) using the jet smearing method. This is compared with the prediction from the Sherpa MC simulation. The quoted uncertainties include those due to statistical and systematic effects (see Sect. 7)

Signal region	Jet-smearing	*Z*+jets MC
SR-Z *ee*	$0.05 \pm 0.04$	$0.05 \pm 0.03$
SR-Z $\mu \mu$	$0.02^{+0.03}_{-0.02}$	$0.09 \pm 0.05$

### Estimation of the flavour-symmetric backgrounds

The dominant background in the signal regions is $t\bar{t}$ production, resulting in two leptons in the final state, with lesser contributors including the production of dibosons (*WW*), single top quarks (*Wt*) and *Z* bosons that decay to $\tau$ leptons. For these the so-called “flavour-symmetry” method can be used to estimate, in a data-driven way, the contribution from these processes in the same-flavour channels using their measured contribution to the different-flavour channels.

#### Flavour-symmetric background in the on-*Z* search

The flavour-symmetry method uses a control region, CR$e\mu$ in the case of the on-*Z* search, which is defined to be identical to the signal region, but in the different-flavour $e\mu$ channel. In CR$e\mu$, the expected contamination due to GGM signal processes of interest is $<$3 %.

The number of data events observed ($N^{\text {data}}_{e\mu }$) in this control region is corrected by subtracting the expected contribution from backgrounds that are not flavour symmetric. The background with the largest impact on this correction is that due to fake leptons, with the estimate provided by the matrix method, described in Sect. 6.3, being used in the subtraction. All other contributions, which include *WZ*, *ZZ*, *tZ* and $t\bar{t}+W(W)/Z$ processes, are taken directly from MC simulation. This corrected number, $N^{\text {data,corr}}_{e\mu }$, is related to the expected number in the same-flavour channels, $N^{\text {est}}_{ee/\mu \mu }$, by the following relations:

2 $$\begin{aligned} N_{ee}^{\mathrm {est}}= & {} \frac{1}{2} N_{e\mu }^{\mathrm {data,corr}} k_{ee} \alpha , \nonumber \\ N_{\mu \mu }^{\mathrm {est}}= & {} \frac{1}{2} N_{e\mu }^{\mathrm {data,corr}} k_{\mu \mu } \alpha , \end{aligned}$$

where $k_{ee}$ and $k_{\mu \mu }$ are electron and muon selection efficiency factors and $\alpha$ accounts for the different trigger efficiencies for same-flavour and different-flavour dilepton combinations. The selection efficiency factors are calculated using the ratio of dielectron and dimuon events in VRZ according to:

3 $$\begin{aligned} k_{ee}= & {} \sqrt{\frac{N_{ee}^{\mathrm {data}}\mathrm{(VRZ)}}{N_{\mu \mu }^{\mathrm {data}}\mathrm{(VRZ)}}}, \nonumber \\ k_{\mu \mu }= & {} \sqrt{\frac{N_{\mu \mu }^{\mathrm {data}}\mathrm{(VRZ)}}{N_{ee}^{\mathrm {data}}\mathrm{(VRZ)}}}, \nonumber \\ \alpha= & {} \frac{\sqrt{\epsilon _{\mathrm {trig}}^{ee} \epsilon _{\mathrm {trig}}^{\mu \mu }}}{\epsilon _{\mathrm {trig}}^{e\mu }}, \end{aligned}$$

where $\epsilon _{\mathrm {trig}}^{ee}$, $\epsilon _{\mathrm {trig}}^{\mu \mu }$ and $\epsilon _{\mathrm {trig}}^{e\mu }$ are the efficiencies of the dielectron, dimuon and electron–muon trigger configurations, respectively, and $N_{ee(\mu \mu )}^{\mathrm {data}}\mathrm{(VRZ)}$ is the number of *ee* ($\mu \mu$) data events in VRZ. These selection efficiency factors are calculated separately for the cases where both leptons fall within the barrel, both fall within the endcap regions, and for barrel–endcap combinations. This is motivated by the fact that the trigger efficiencies differ in the central and more forward regions of the detector. This estimate is found to be consistent with that resulting from the use of single global *k* factors, which provides a simpler but less precise estimate. In each case the *k* factors are close to 1.0, and the $N_{ee}^{\mathrm {est}}$ or $N_{\mu \mu }^{\mathrm {est}}$ estimates obtained using *k* factors from each configuration are consistent with one another to within $0.2\sigma$.

**Fig. 3:**
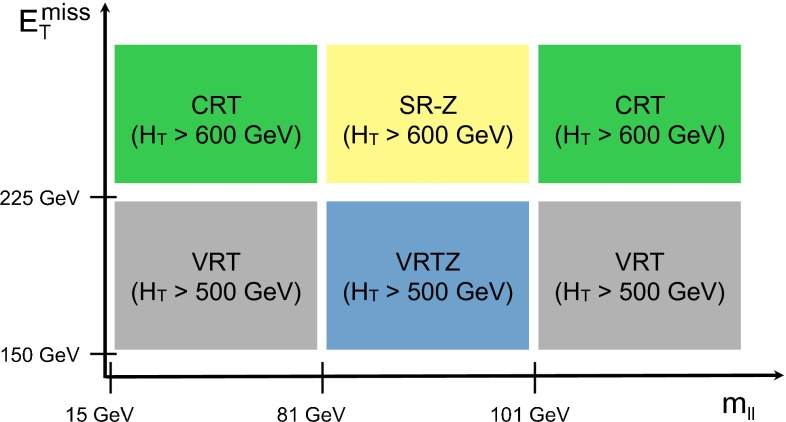
Diagram indicating the position in the ${E_{\mathrm{T}}^{\mathrm{miss}}}$ versus dilepton invariant mass plane of SR-Z, the control region CRT, and the two validation regions (VRT and VRTZ) used to validate the sideband fit for the on-*Z* search. VRT and VRTZ have lower ${H_{\mathrm{T}}}$ thresholds than CRT and SR-Z

The flavour-symmetric background estimate was chosen as the nominal method prior to examining the data yields in the signal region, since it relies less heavily on simulation and provides the most precise estimate. This data-driven method is cross-checked using the *Z* boson mass sidebands ($$m_{\ell \ell } \notin [81, 101]$$ GeV) to fit the $$t\bar{t}$$ MC events to data in a top control region, CRT. The results are then extrapolated to the signal region in the *Z* boson mass window, as illustrated in Fig. 3. All other backgrounds estimated using the flavour-symmetry method are taken directly from MC simulation for this cross-check. Here, $${Z/\gamma ^{*}+\text {jets}}$$ MC events are used to model the small residual $${Z/\gamma ^{*}+\text {jets}}$$ background in the control region, while the jet smearing method provides the estimate in the signal region. The normalisation of the $$t\bar{t}$$ sample obtained from the fit is $$0.52 \pm 0.12$$ times the nominal MC normalisation, where the uncertainty includes all experimental and theoretical sources of uncertainty as discussed in Sect. 7. This result is compatible with observations from other ATLAS analyses, which indicate that MC simulation tends to overestimate data in regions dominated by $$t\bar{t}$$ events accompanied by much jet activity [79, 80]. MC simulation has also been seen to overestimate contributions from $$t\bar{t}$$ processes in regions with high $${E_{\mathrm{T}}^{\mathrm{miss}}} $$ [81]. In selections with high $${E_{\mathrm{T}}^{\mathrm{miss}}} $$  but including lower $$H_{\mathrm{T}}$$, such as those used in the off-*Z* analysis, this downwards scaling is less dramatic. The results of the cross-check using the *Z* boson mass sidebands are shown in Table 5, with the sideband fit yielding a prediction slightly higher than, but consistent with, the flavour-symmetry estimate. This test is repeated varying the MC simulation sample used to model the $$t\bar{t}$$ background. The nominal Powheg+Pythia$$t\bar{t}$$ MC sample is replaced with a sample using Alpgen, and the fit is performed again. The same test is performed using a Powheg$$t\bar{t}$$ MC sample that uses Herwig, rather than Pythia, for the parton shower. In all cases the estimates are found to be consistent within $$1 \sigma $$. This cross-check using $$t\bar{t}$$ MC events is further validated in identical regions with intermediate $${E_{\mathrm{T}}^{\mathrm{miss}}} $$ (150 $$<$$$${E_{\mathrm{T}}^{\mathrm{miss}}} $$$$<$$ 225 GeV) and slightly looser $${H_{\mathrm{T}}} $$ requirements ($${H_{\mathrm{T}}} >500$$ GeV), as illustrated in Fig. 3. Here the extrapolation in $$m_{\ell \ell }$$ between the sideband region (VRT) and the on-*Z* region (VRTZ) shows consistent results within approximately $$1\sigma $$ between data and the fitted prediction.

**Table 5:** The number of events for the flavour-symmetric background estimate in the on-*Z* signal region (SR-Z) using the data-driven method based on data in CR$e\mu$. This is compared with the prediction for the sum of the flavour-symmetric backgrounds (*WW*, *tW*, $t\bar{t}$ and $Z \rightarrow \tau \tau$) from a sideband fit to data in CRT. In each case the combined statistical and systematic uncertainties are indicated

Signal region	Flavour-symmetry	Sideband fit
SR-Z *ee*	$2.8 \pm 1.4$	$4.9 \pm 1.5$
SR-Z $\mu \mu$	$3.3 \pm 1.6$	$5.3 \pm 1.9$

**Fig. 4:**
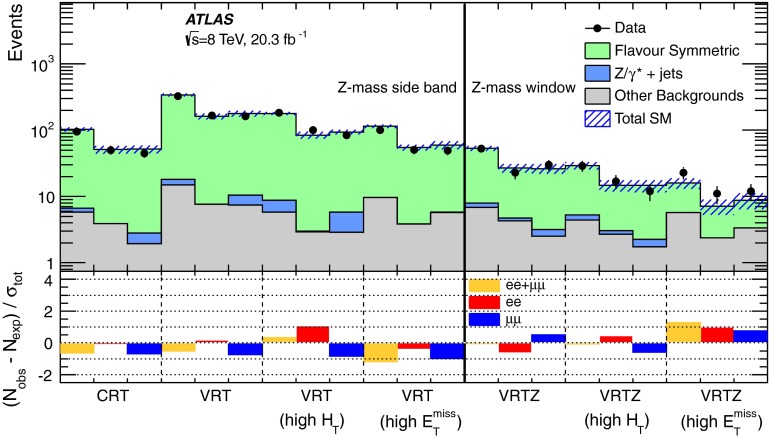
The observed and expected yields in CRT and the VRs in the *Z* boson mass sidebands (*left*) and the *Z* boson mass window (*right*) regions. The *bottom plot* shows the difference in standard deviations between the observed and expected yields. The backgrounds due to *WZ*, *ZZ* or rare top processes, as well as from lepton fakes, are included under “Other Backgrounds”

The flavour-symmetry method is also tested in these VRs. An overview of the nominal background predictions, using the flavour-symmetry method, in CRT and these VRs is shown in Fig. 4. This summary includes CRT, VRT, VRTZ and two variations of VRT and VRTZ. The first variation, denoted VRT/VRTZ (high $H_{\mathrm{T}}$), shows VRT/ VRTZ with an increased $H_{\mathrm{T}}$ threshold ($H_{\mathrm{T}}>600$ GeV), which provides a sample of events very close to the SR. The second variation, denoted VRT/VRTZ (high ${E_{\mathrm{T}}^{\mathrm{miss}}}$), shows VRT/ VRTZ with the same ${E_{\mathrm{T}}^{\mathrm{miss}}}$ cut as SR-Z, but the requirement $400<H_{\mathrm{T}}<600$ GeV is added to provide a sample of events very close to the SR. In all cases the data are consistent with the prediction. GGM signal processes near the boundary of the expected excluded region are expected to contribute little to the normalisation regions, with contamination at the level of up to 4 % in CRT and 3 % in VRT. The corresponding contamination in VRTZ is expected to be $\sim$10 % across most of the relevant parameter space, increasing to a maximum value of $\sim$50 % in the region near $m(\tilde{g})=700$ ${\mathrm {GeV}}$, $\mu =200$ ${\mathrm {GeV}}$.

#### Flavour-symmetric background in the off-*Z* search

The background estimation method of Eq. (2) is extended to allow a prediction of the background dilepton mass shape, which is used explicitly to discriminate signal from background in the off-*Z* search. In addition to the *k* and $\alpha$ correction factors, a third correction factor *S*(*i*) is introduced (where *i* indicates the dilepton mass bin):

4 $$\begin{aligned} N_{ee}^{\mathrm {est}}(i)= & {} \frac{1}{2} N_{e\mu }^{\mathrm {data,corr}}(i) k_{ee} \alpha S_{ee}(i), \nonumber \\ N_{\mu \mu }^{\mathrm {est}}(i)= & {} \frac{1}{2} N_{e\mu }^{\mathrm {data,corr}}(i) k_{\mu \mu } \alpha S_{\mu \mu }(i). \end{aligned}$$

These shape correction factors account for different reconstructed dilepton mass shapes in the *ee*, $\mu \mu$, and $e\mu$ channels, which result from two effects. First, the offline selection efficiencies for electrons and muons depend differently on the lepton ${p_{\mathrm{T}}}$ and $\eta$. For electrons, the offline selection efficiency increases slowly with ${p_{\mathrm{T}}}$, while it has very little dependence on ${p_{\mathrm{T}}}$ for muons. Second, the combinations of single-lepton and dilepton triggers used for the *ee*, $\mu \mu$, and $e\mu$ channels have different efficiencies with respect to the offline selection. In particular, for $e\mu$ events the trigger efficiency with respect to the offline selection at low ${m_{\ell \ell }}$ is 80 %, which is 10–15 % lower than the trigger efficiencies in the *ee* and $\mu \mu$ channels. To correct for these two effects, $t\bar{t}$ MC simulation is used. The dilepton mass shape in the *ee* or $\mu \mu$ channel is compared to that in the $e\mu$ channel, after scaling the latter by the $\alpha$- and *k*-factor trigger and lepton selection efficiency corrections. The ratio of the dilepton mass distributions, $N_{ee}({m_{\ell \ell }})/N_{e\mu }({m_{\ell \ell }})$ or $N_{\mu \mu }({m_{\ell \ell }})/N_{e\mu }({m_{\ell \ell }})$, is fitted with a second-order polynomial, which is then applied as a correction factor, along with $\alpha$ and *k*, to the $e\mu$ distribution in data. These correction factors have an impact on the predicted background yields of approximately a few percent in the *ee* channel and up to $\sim$10–15 % in the $\mu \mu$ channel, depending on the signal region.

**Fig. 5:**
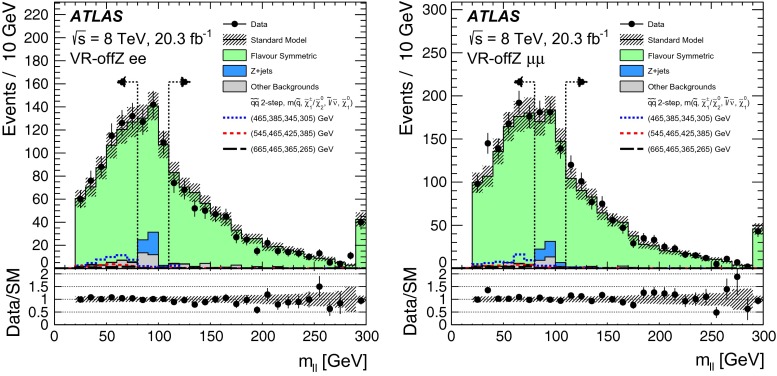
The observed and expected dilepton mass distributions in the electron (*left*) and muon (*right*) channel of the validation region (VR-offZ) of the off-*Z* search. Data (*black points*) are compared to the sum of expected backgrounds (*solid histograms*). The *vertical dashed lines* indicate the $80<{m_{\ell \ell }}<110$ ${\mathrm {GeV}}$ region, which is used to normalise the ${Z+\text {jets}}$ background. Example signal models (*dashed lines*) are overlaid, with $m(\tilde{q})$, $m(\tilde{\chi }^{0}_{2})/m(\tilde{\chi }^{\pm }_{1})$, $m(\tilde{\ell })/m(\tilde{\nu })$, and $m(\tilde{\chi }^{0}_{1})$ of each benchmark point being indicated in the figure legend. The *bottom plots* show the ratio of the data to expected background. The *error bars* indicate the statistical uncertainty in data, while the *shaded band* indicates the total background uncertainty. The last bin contains the overflow

The background estimation methodology is validated in a region with exactly two jets and $100<{E_{\mathrm{T}}^{\mathrm{miss}}} <150$ ${\mathrm {GeV}}$ (VR-offZ). The flavour-symmetric category contributes more than 95 % of the total background in this region. The dominant systematic uncertainty on the background prediction is the 6 % uncertainty on the trigger efficiency $\alpha$-factor. The observed dilepton mass shapes are compared to the SM expectations in Fig. 5, indicating consistency between the data and the expected background yields. The observed yields and expected backgrounds in the below-*Z* and above-*Z* regions are presented in Sect. 1. For signal models near the edge of the sensitivity of this analysis, the contamination from signal events in VR-offZ is less than 3 %.

### Fake-lepton contribution

Events from $W \rightarrow \ell \nu +$jets, semileptonic $t\bar{t}$ and single top (*s*- and *t*-channel) contribute to the background in the dilepton channels due to “fake” leptons. These include leptons from *b*-hadron decays, misidentified hadrons or converted photons, and are estimated from data using a matrix method, which is described in detail in Ref. [82]. This method involves creating a control sample using baseline leptons, thereby loosening the lepton isolation and identification requirements and increasing the probability of selecting a fake lepton. For each control or signal region, the relevant requirements are applied to this control sample, and the number of events with leptons that pass or fail the subsequent signal-lepton requirements are counted. Denoting the number of events passing signal lepton requirements by $N_{\mathrm{pass}}$ and the number failing by $N_{\mathrm{fail}}$, the number of events containing a fake lepton for a single-lepton selection is given by

5 $$\begin{aligned} N_{\mathrm{fake}} = \frac{N_{\mathrm{fail}} - (1/\epsilon ^{\mathrm{real}}-1) N_{\mathrm{pass}}}{(1/\epsilon ^{\mathrm{fake}} - 1/\epsilon ^{\mathrm{real}})}, \end{aligned}$$

where $\epsilon ^{\mathrm{fake}}$ is the efficiency with which fake leptons passing the baseline lepton selection also pass signal lepton requirements and $\epsilon ^{\mathrm{real}}$ is the relative identification efficiency (from baseline to signal lepton selection) for real leptons. This principle is expanded to a dilepton sample using a four-by-four matrix to account for the various possible real–fake combinations for the two leading leptons in the event.

The efficiency for fake leptons is estimated in control regions enriched with multi-jet events. Events are selected if they contain at least one baseline lepton, one signal jet with ${p_{\mathrm{T}}}$ $>60$ GeV and low ${E_{\mathrm{T}}^{\mathrm{miss}}}$ ($<$30 GeV). The background due to processes containing prompt leptons, estimated from MC samples, is subtracted from the total data contribution in this region. From the resulting data sample the fraction of events in which the baseline leptons pass signal lepton requirements gives the fake efficiency. This calculation is performed separately for events with *b*-tagged jets and those without to take into account the various sources from which fake leptons originate. The real-lepton efficiency is estimated using $Z \rightarrow \ell ^+ \ell ^-$ events in a data sample enriched with leptonically decaying *Z* bosons. Both the real-lepton and fake-lepton efficiencies are further binned as a function of ${p_{\mathrm{T}}}$ and $\eta$.

### Estimation of other backgrounds

The remaining background processes, including diboson events with a *Z* boson decaying to leptons and the $t\bar{t}+W(W)/Z$ and $t+Z$ backgrounds, are estimated from MC simulation. In these cases the most accurate theoretical cross sections available are used, as summarised in Table 1. Care is taken to ensure that the flavour-symmetric component of these backgrounds (for events where the two leptons do not originate from the same *Z* decay) is not double-counted.

## Systematic uncertainties

Systematic uncertainties have an impact on the predicted signal region yields from the dominant backgrounds, the fake-lepton estimation, and the yields from backgrounds predicted using simulation alone. The expected signal yields are also affected by systematic uncertainties. All sources of systematic uncertainty considered are discussed in the following subsections.

### Experimental uncertainties

The experimental uncertainties arise from the modelling of both the signal processes and backgrounds estimated using MC simulation. Uncertainties associated with the jet energy scale (JES) are assessed using both simulation and in-situ measurements [70, 71]. The JES uncertainty is influenced by the event topology, flavour composition, jet ${p_{\mathrm{T}}}$ and $\eta$, as well as by the pile-up. The jet energy resolution (JER) is also affected by pile-up, and is estimated using in-situ measurements [83]. An uncertainty associated with the JVF requirement for selected jets is also applied by varying the JVF threshold up (0.28) and down (0.21) with respect to the nominal value of 0.25. This range of variation is chosen based on a comparison of the efficiency of a JVF requirement in dijet events in data and MC simulation.

To distinguish between heavy-flavour-enriched and light-flavour-enriched event samples, *b*-jet tagging is used. The uncertainties associated with the *b*-tagging efficiency and the light/charm quark mis-tag rates are measured in $t\bar{t}$-enriched samples [84, 85] and dijet samples [86], respectively.

Small uncertainties on the lepton energy scales and momentum resolutions are measured in $Z \rightarrow \ell ^+\ell ^-$, $J/\psi \rightarrow \ell ^+\ell ^-$ and $W \rightarrow \ell ^{\pm }\nu$ event samples [64]. These are propagated to the ${E_{\mathrm{T}}^{\mathrm{miss}}}$ uncertainty, along with the uncertainties due to the JES and JER. An additional uncertainty on the energy scale of topological clusters in the calorimeters not associated with reconstructed objects (the ${E_{\mathrm{T}}^{\mathrm{miss}}}$ soft term) is also applied to the ${E_{\mathrm{T}}^{\mathrm{miss}}}$ calculation.

The trigger efficiency is assigned a 5 % uncertainty following studies comparing the efficiency in simulation to that measured in $Z \rightarrow \ell ^+ \ell ^-$ events in data.

The data-driven background estimates are subject to uncertainties associated with the methods employed and the limited number of events used in their estimation. The ${Z/\gamma ^{*}+\text {jets}}$ background estimate has an uncertainty to account for differences between pseudo-data and MC events, the choice of seed region definition, the statistical precision of the seed region, and the jet response functions used to create the pseudo-data. Uncertainties in the flavour-symmetric background estimate include those related to the electron and muon selection efficiency factors $k_{ee}$ and $k_{\mu \mu }$, the trigger efficiency factor $\alpha$, and, for the off-*Z* search only, the dilepton mass shape *S*(*i*) reweighting factors. Uncertainties attributed to the subtraction of the non-flavour-symmetric backgrounds, and those due to limited statistical precision in the $e\mu$ control regions, are also included. Finally, an uncertainty derived from the difference in real-lepton efficiency observed in $t\bar{t}$ and $Z \rightarrow \ell ^+ \ell ^-$ events is assigned to the fake-background prediction. An additional uncertainty due to the number of events in the control samples used to derive the real efficiencies and fake rates is assigned to this background, as well as a 20 % uncertainty on the MC background subtraction in the control samples.

### Theoretical uncertainties on background processes

For all backgrounds estimated from MC simulation, the following theoretical uncertainties are considered. The uncertainties due to the choice of factorisation and renormalisation scales are calculated by varying the nominal values by a factor of two. Uncertainties on the PDFs are evaluated following the prescription recommended by PDF4LHC [87]. Total cross-section uncertainties of 22 % [37] and 50 % are applied to $t\bar{t}$ $+W$/*Z* and $t\bar{t}$ $+WW$ sub-processes, respectively. For the $t\bar{t}$ $+W$ and $t\bar{t}$ $+Z$ sub-processes, an additional uncertainty is evaluated by comparing samples generated with different numbers of partons, to account for the impact of the finite number of partons generated in the nominal samples. For the *WZ* and *ZZ* diboson samples, a parton shower uncertainty is estimated by comparing samples showered with Pythia and Herwig+Jimmy [88, 89] and cross-section uncertainties of 5 and 7 % are applied, respectively. These cross-section uncertainties are estimated from variations of the value of the strong coupling constant, the PDF and the generator scales. For the small contribution from $t+Z$, a 50 % uncertainty is assigned. Finally, a statistical uncertainty derived from the finite size of the MC samples used in the background estimation process is included.

### Dominant uncertainties on the background estimates

The dominant uncertainties in each signal region, along with their values relative to the total background expectation, are summarised in Table 6. In all signal regions the largest uncertainty is that associated with the flavour-symmetric background. The statistical uncertainty on the flavour-symmetric background due to the finite data yields in the $e\mu$ CRs is 24 % in the on-*Z* SR. This statistical uncertainty is also the dominant uncertainty for all SRs of the off-*Z* analysis except for SR-loose, for which the systematic uncertainty on the flavour-symmetric background prediction dominates. In SR-Z the combined MC generator and parton shower modelling uncertainty on the *WZ* background (7 %), as well as the uncertainty due to the fake-lepton background (14 %), are also important.

**Table 6:** Overview of the dominant sources of systematic uncertainty on the background estimate in the signal regions. Their relative values with respect to the total background expectation are shown (in %). For the off-*Z* region, the full dilepton mass range is used, and in all cases the $ee+\mu \mu$ contributions are considered together

Source	Relative systematic uncertainty (%)					
	SR-Z	SR-loose	SR-2j-bveto	SR-2j-btag	SR-4j-bveto	SR-4j-btag
Total systematic uncertainty	29	7.1	13	9.3	30	15
Flavour-symmetry statistical	24	1.7	9.3	6.2	23	12
Flavour-symmetry systematic	4	5.7	6.7	5.9	11	6.6
${Z/\gamma ^{*}+\text {jets}}$	–	2.1	6.3	3.5	14	7.0
Fake lepton	14	3.2	1.4	1.2	1.8	2.2
*WZ* MC $+$ parton shower	7	–	–	–	–	–

### Theoretical uncertainties on signal processes

Signal cross sections are calculated to next-to-leading order in the strong coupling constant, adding the resummation of soft gluon emission at NLO+NLL accuracy [55–59]. The nominal cross section and the uncertainty are taken from an envelope of cross-section predictions using different PDF sets and factorisation and renormalisation scales, as described in Ref. [90]. For the simplified models the uncertainty on the initial-state radiation modelling is important in the case of small mass differences during the cascade decays. MadGraph+Pythia samples are used to assess this uncertainty, with the factorisation and normalisation scale, the MadGraph parameter used for jet matching, the MadGraph parameter used to set the QCD radiation scale and the Pythia parameter responsible for the value of the QCD scale for final-state radiation, each being varied up and down by a factor of two. The resulting uncertainty on the signal acceptance is up to $\sim$25 % in regions with small mass differences within the decay chains.

## Results

For the on-*Z* search, the resulting background estimates in the signal regions, along with the observed event yields, are displayed in Table 7. The dominant backgrounds are those due to flavour-symmetric and *WZ* and *ZZ* diboson processes. In the electron and muon channel combined, $10.6\pm 3.2$ events are expected and 29 are observed. For each of these regions, a local probability for the background estimate to produce a fluctuation greater than or equal to the excess observed in the data is calculated using pseudo-experiments. When expressed in terms of the number of standard deviations, this value is referred to as the local significance, or simply the significance. These significances are quantified in the last column of Table 11 and correspond to a $1.7\sigma$ deviation in the muon channel and a $3.0\sigma$ deviation in the electron channel, with the combined significance, calculated from the sum of the background predictions and observed yields in the muon and electron channels, being $3.0\sigma$. The uncertainties on the background predictions in the *ee* and $\mu \mu$ channels are correlated as they are dominated by the statistical uncertainty of the $e\mu$ data sample that is used to derive the flavour-symmetric background in both channels. Since this sample is common to both channels, the relative statistical error on the flavour-symmetric background estimation does not decrease when combining the *ee* and $\mu \mu$ channels. No excess was reported in the CMS analysis of the $Z+\mathrm {jets}+{E_{\mathrm{T}}^{\mathrm{miss}}}$ final state based on $\sqrt{s}=8\,{\mathrm {TeV}}$ data [24]; however, the kinematic requirements used in that search differ from those used in this paper.

**Table 7:** Results in the on-*Z* SRs (SR-Z). The flavour symmetric, ${Z/\gamma ^{*}+\text {jets}}$ and fake-lepton background components are all derived using data-driven estimates described in the text. All other backgrounds are taken from MC simulation. The displayed uncertainties include the statistical and systematic uncertainty components combined

Channel	SR-Z *ee*	SR-Z $\mu \mu$	SR-Z same-flavour combined
Observed events	16	13	29
Expected background events	$4.2 \pm 1.6$	$6.4 \pm 2.2$	$10.6 \pm 3.2$
Flavour-symmetric backgrounds	$2.8 \pm 1.4$	$3.3 \pm 1.6$	$6.0 \pm 2.6$
${Z/\gamma ^{*}+\text {jets}}$ (jet-smearing)	$0.05 \pm 0.04$	$0.02_{-0.02}^{+0.03}$	$0.07 \pm 0.05$
Rare top	$0.18 \pm 0.06$	$0.17 \pm 0.06$	$0.35 \pm 0.12$
*WZ*/*ZZ* diboson	$1.2 \pm 0.5$	$1.7 \pm 0.6$	$2.9 \pm 1.0$
Fake leptons	$0.1_{-0.1}^{+0.7}$	$1.2_{-1.2}^{+1.3}$	$1.3_{-1.3}^{+1.7}$

**Fig. 6:**
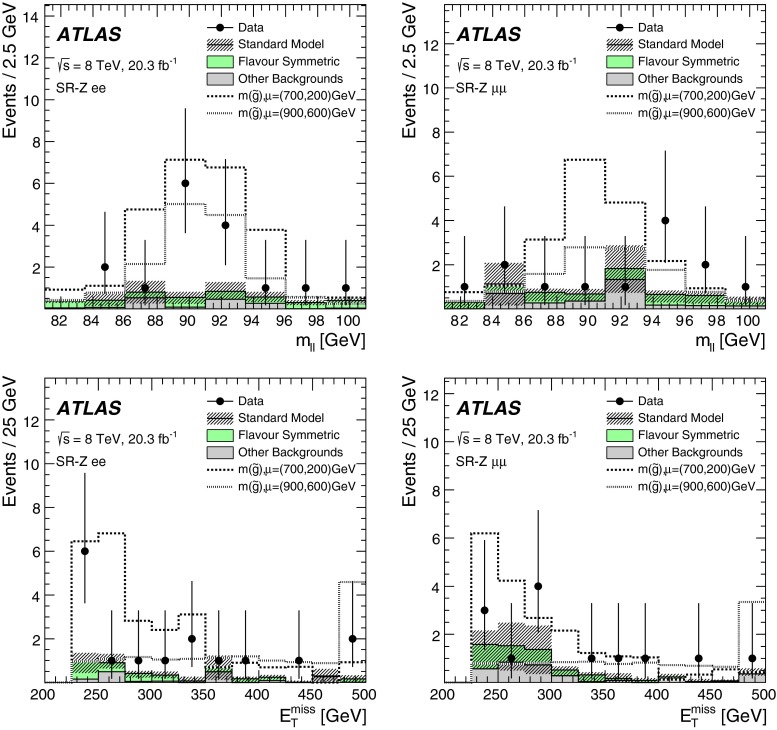
The dilepton mass (*top*) and ${E_{\mathrm{T}}^{\mathrm{miss}}}$ (*bottom*) distributions for the electron (*left*) and muon (*right*) channel in the on-*Z* SRs after having applied the requirement $\Delta \phi (\text {jet}_{1,2},{E_{\mathrm{T}}^{\mathrm{miss}}})>0.4$. All uncertainties are included in the *hatched uncertainty band*. Two example GGM ($\tan \beta =1.5$) signal models are overlaid. For the ${E_{\mathrm{T}}^{\mathrm{miss}}}$ distributions, the last bin contains the overflow. The backgrounds due to *WZ*, *ZZ* or rare top processes, as well as from fake leptons, are included under “Other Backgrounds”. The negligible contribution from *Z*+jets is omitted from these distributions

**Fig. 7:**
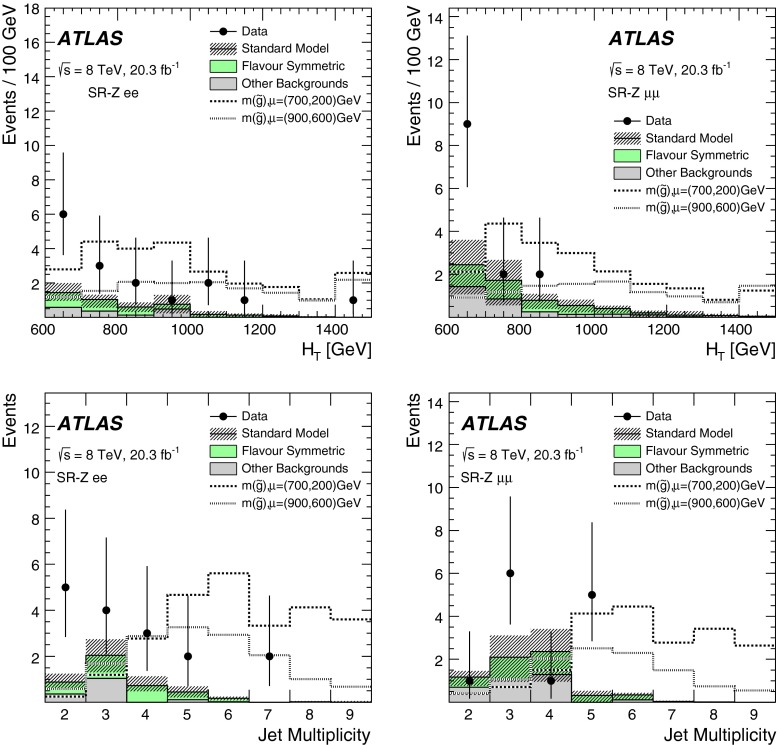
The $H_{\mathrm{T}}$ (*top*) and jet multiplicity (*bottom*) distributions for the electron (*left*) and muon (*right*) channel in the on-*Z* SRs after having applied the requirement $\Delta \phi (\text {jet}_{1,2},{E_{\mathrm{T}}^{\mathrm{miss}}})>0.4$. All uncertainties are included in the *hatched uncertainty band*. Two example GGM ($\tan \beta =1.5$) signal models are overlaid. For the $H_{\mathrm{T}}$ distributions, the last bin contains the overflow. The backgrounds due to *WZ*, *ZZ* or rare top processes, as well as from fake leptons, are included under “Other Backgrounds”. The negligible contribution from *Z*+jets is omitted from these distributions

Dilepton invariant mass and ${E_{\mathrm{T}}^{\mathrm{miss}}}$ distributions in the electron and muon on-*Z* SR are shown in Fig. 6, with $H_{\mathrm{T}}$ and jet multiplicity being shown in Fig. 7. For the SR selection a requirement is imposed to reject events with $\Delta \phi (\text {jet}_{1,2},{E_{\mathrm{T}}^{\mathrm{miss}}})<0.4$ to further suppress the background from ${Z/\gamma ^{*}+\text {jets}}$ processes with mismeasured jets.

**Fig. 8:**
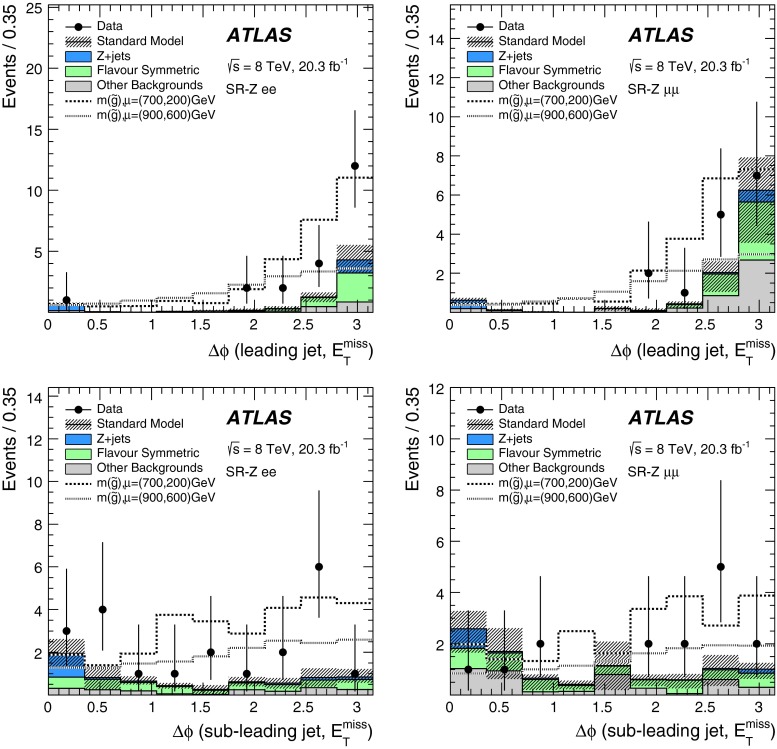
The distribution of the $\Delta \phi$ between the leading jet and ${E_{\mathrm{T}}^{\mathrm{miss}}}$ (*top*) and the sub-leading jet and ${E_{\mathrm{T}}^{\mathrm{miss}}}$ (*bottom*) for the electron (*left*) and muon (*right*) channel in the on-*Z* SRs before having applied the requirement $\Delta \phi (\text {jet}_{1,2},{E_{\mathrm{T}}^{\mathrm{miss}}})>0.4$. All uncertainties are included in the *hatched uncertainty band*. Two example GGM ($\tan \beta =1.5$) signal models are overlaid. The backgrounds due to *WZ*, *ZZ* or rare top processes, as well as from fake leptons, are included under “Other Backgrounds”

In Fig. 8, the distribution of events in the on-*Z* SR as a function of $$\Delta \phi (\text {jet}_{1,2},$$$${E_{\mathrm{T}}^{\mathrm{miss}}})$$ (before this requirement is applied) is shown. In these figures the shapes of the flavour-symmetric and $${Z/\gamma ^{*}+\text {jets}}$$ backgrounds are derived using MC simulation and the normalisation is taken according to the data driven estimate.

**Fig. 9:**
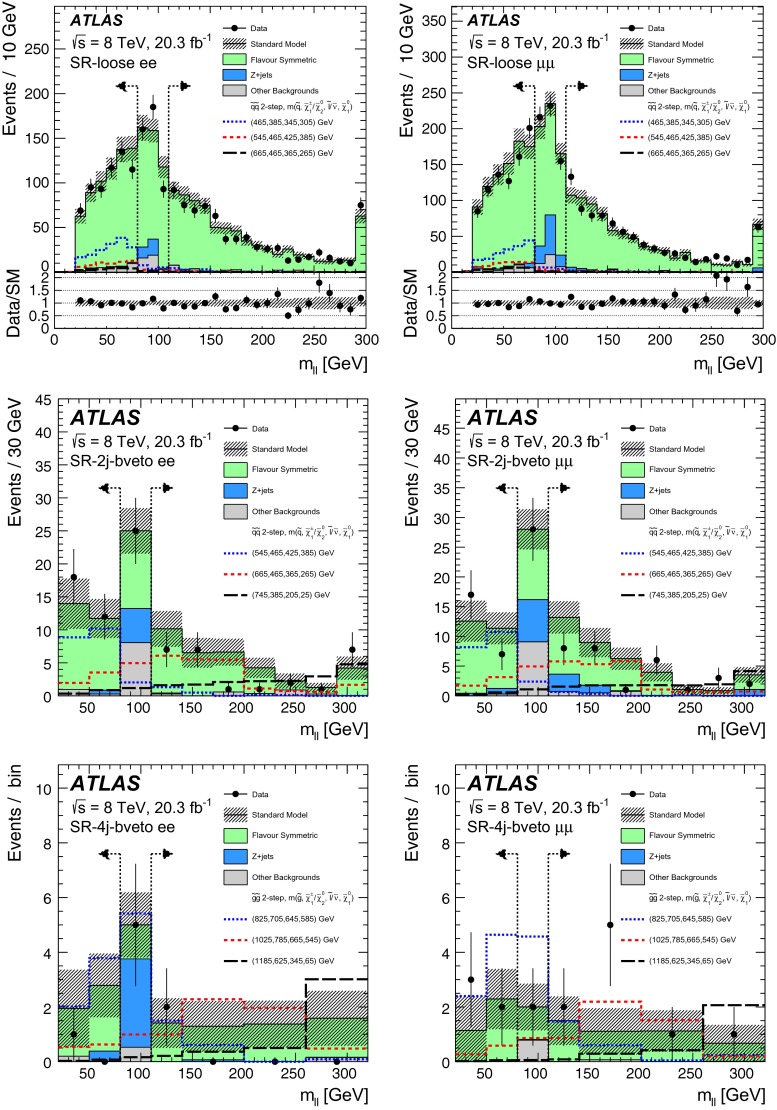
The observed and expected dilepton mass distributions in the off-*Z* SR-loose (*top*), SR-2j-bveto (*middle*), and SR-4j-bveto (*bottom*). The *vertical dashed lines* indicate the $80<{m_{\ell \ell }}<110$ ${\mathrm {GeV}}$ region, which is used to normalise the ${Z+\text {jets}}$ background and is thus not treated as a search region. Example signal models (*dashed lines*) are overlaid, with $m(\tilde{q})/m(\tilde{g})$, $m(\tilde{\chi }^{0}_{2})/m(\tilde{\chi }^{\pm }_{1})$, $m(\tilde{\ell })/m(\tilde{\nu })$, and $m(\tilde{\chi }^{0}_{1})$ of each benchmark point being indicated in the figure legend. The last bin contains the overflow. All uncertainties are included in the *hatched uncertainty band*

**Fig. 10:**
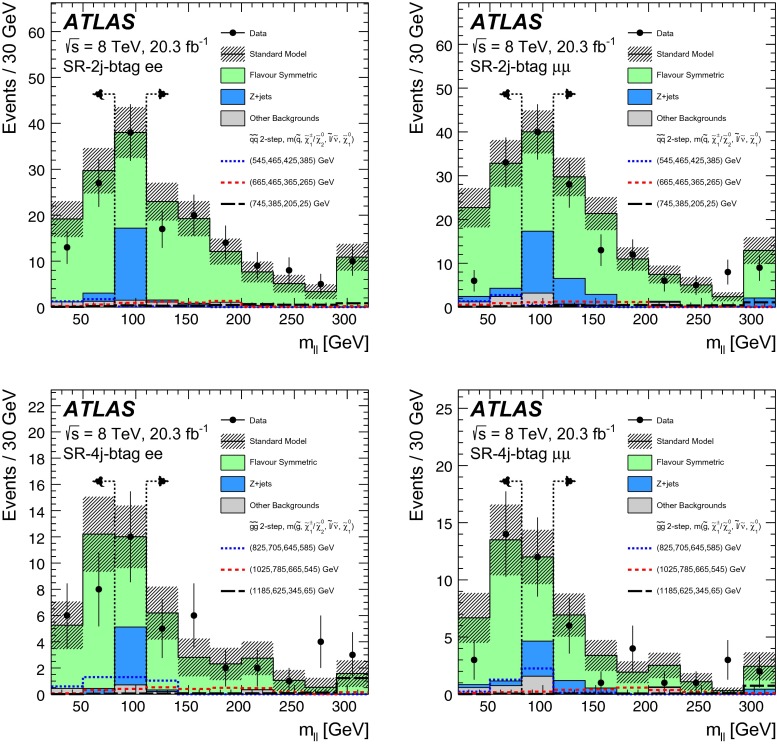
The observed and expected dilepton mass distributions in the SR-2j-btag (*top*) and SR-4j-btag (*bottom*) signal regions of the off-*Z* search. The *vertical dashed lines* indicate the $80<{m_{\ell \ell }}<110$ ${\mathrm {GeV}}$ region, which is used to normalise the ${Z+\text {jets}}$ background and is thus not treated as a search region. Example signal models of squark- or gluino-pair production (*dashed lines*) are overlaid, with $m(\tilde{g})$, $m(\tilde{\chi }^{0}_{2})/m(\tilde{\chi }^{\pm }_{1})$, $m(\tilde{\ell })/m(\tilde{\nu })$, and $m(\tilde{\chi }^{0}_{1})$ of each benchmark point being indicated in the figure legend. The last bin contains the overflow. All uncertainties are included in the *hatched uncertainty band*

**Fig. 11:**
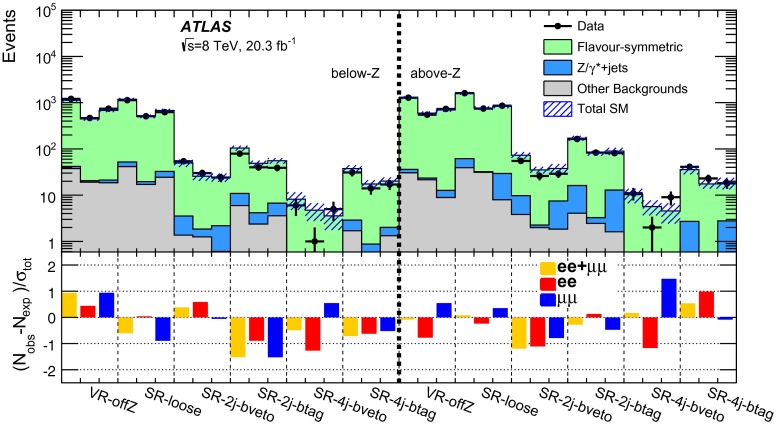
The observed and expected yields in the below-*Z* (*left*) and above-*Z* (*right*) dilepton mass regions, for the VR and five SRs of the off-*Z* search. Here below-*Z* is $20<{m_{\ell \ell }}<70$ ${\mathrm {GeV}}$ for VR-offZ and SR-loose and otherwise $20<{m_{\ell \ell }}<80$ ${\mathrm {GeV}}$; above-*Z* is ${m_{\ell \ell }}>110$ ${\mathrm {GeV}}$. The *bottom plot* shows the difference in standard deviations between the observed and expected yields. Results are shown for the *ee* and $\mu \mu$ channels as well as for the sum

For the off-*Z* search, the dilepton mass distributions in the five SRs are presented in Figs. 9 and 10, and summarised in Fig. 11. The expected backgrounds and observed yields in the below-*Z* and above-*Z* regions for SR-2j-bveto, SR-4j-bveto, and SR-loose are presented in Tables 8, 9, and 10, respectively. Corresponding results for SR-2j-btag and SR-4j-btag are presented in Sect. 1. The data are consistent with the expected SM backgrounds in all regions. In the SR-loose region with $$20<{m_{\ell \ell }}<70$$$${\mathrm {GeV}}$$, similar to the region in which the CMS Collaboration observed a 2.6$$\sigma $$ excess, 1133 events are observed, compared to an expectation of $$1190\pm 40\pm 70$$ events.

**Table 8:** Results in the off-*Z* search region SR-2j-bveto, in the below-*Z* range ($20<{m_{\ell \ell }}<80$ ${\mathrm {GeV}}$, top) and above-*Z* range (${m_{\ell \ell }}>110$ ${\mathrm {GeV}}$, bottom). The flavour symmetric, ${Z/\gamma ^{*}+\text {jets}}$ and fake lepton background components are all derived using data-driven estimates described in the text. All other backgrounds are taken from MC simulation. The first uncertainty is statistical and the second is systematic

	SR-2j-bveto *ee*	SR-2j-bveto $\mu \mu$	SR-2j-bveto same-flavour combined
Below-*Z* ($20<{m_{\ell \ell }}<80$ ${\mathrm {GeV}}$)			
Observed events	30	24	54
Expected background events	$26 \pm 4 \pm 3$	$24 \pm 4 \pm 3$	$50 \pm 8 \pm 5$
Flavour-symmetric backgrounds	$24 \pm 4 \pm 3$	$22 \pm 4 \pm 3$	$46 \pm 8 \pm 4$
${Z/\gamma ^{*}+\text {jets}}$	$0.6\pm 0.3\pm 0.7$	$1.6\pm 0.6\pm 1.4$	$2.2\pm 0.7\pm 1.7$
Rare top	$<$0.1	$<$0.1	$<$0.1
*WZ* / *ZZ* diboson	$0.6\pm 0.2\pm 0.1$	$0.6\pm 0.2\pm 0.2$	$1.2\pm 0.3\pm 0.2$
Fake leptons	$0.6\pm 0.9\pm 0.1$	$<$0.1	$0.2\pm 0.9\pm 0.1$
Above-*Z* (${m_{\ell \ell }}>110$ ${\mathrm {GeV}}$)			
Observed events	26	29	55
Expected background events	$35 \pm 5 \pm 4$	$38 \pm 4 \pm 8$	$73 \pm 9 \pm 9$
Flavour-symmetric backgrounds	$33 \pm 4 \pm 4$	$30 \pm 4 \pm 3$	$63 \pm 8 \pm 5$
${Z/\gamma ^{*}+\text {jets}}$	$0.3\pm 0.2\pm 0.3$	$5.6\pm 0.6\pm 7.5$	$5.9\pm 0.7\pm 7.5$
Rare top	$<$0.1	$<$0.1	$<$0.1
*WZ* / *ZZ* diboson	$0.3\pm 0.1\pm 0.1$	$0.6\pm 0.2\pm 0.1$	$0.8\pm 0.2\pm 0.1$
Fake leptons	$1.7\pm 1.1\pm 0.2$	$1.3\pm 1.1\pm 0.5$	$3.0\pm 1.5\pm 0.4$

**Table 9:** Results in the off-*Z* search region SR-4j-bveto, in the below-*Z* range ($20<{m_{\ell \ell }}<80$ ${\mathrm {GeV}}$, top) and above-*Z* range (${m_{\ell \ell }}>110$ ${\mathrm {GeV}}$, bottom). Details are the same as in Table 8

	SR-4j-bveto *ee*	SR-4j-bveto $\mu \mu$	SR-4j-bveto same-flavour combined
Below-*Z* ($20<{m_{\ell \ell }}<80$ ${\mathrm {GeV}}$)			
Observed events	1	5	6
Expected background events	$4.7\pm 1.6\pm 1.1$	$3.6\pm 1.5\pm 1.0$	$8.2\pm 3.1\pm 1.4$
Flavour-symmetric backgrounds	$4.1\pm 1.6\pm 1.1$	$3.5\pm 1.5\pm 1.0$	$7.7\pm 3.1\pm 1.3$
${Z/\gamma ^{*}+\text {jets}}$	$0.4\pm 0.2\pm 0.3$	$0.0\pm 0.0\pm 0.4$	$0.4\pm 0.2\pm 0.5$
Rare top	$<$0.1	$<$0.1	$<$0.1
*WZ* / *ZZ* diboson	$<$0.1	$<$0.1	$<$0.1
Fake leptons	$0.2\pm 0.3\pm 0.0$	$<$0.1	$0.1\pm 0.3\pm 0.0$
Above-*Z* (${m_{\ell \ell }}>110$ ${\mathrm {GeV}}$)			
Observed events	2	9	11
Expected background events	$5.7\pm 1.6\pm 1.2$	$4.5\pm 1.3\pm 1.7$	$10 \pm 3 \pm 2$
Flavour-symmetric backgrounds	$5.5\pm 1.6\pm 1.2$	$4.3\pm 1.3\pm 1.0$	$9.8\pm 2.9\pm 1.4$
${Z/\gamma ^{*}+\text {jets}}$	$0.2\pm 0.1\pm 0.1$	$0.0\pm 0.0\pm 1.3$	$0.2\pm 0.1\pm 1.3$
Rare top	$<$0.1	$<$0.1	$<$0.1
*WZ* / *ZZ* diboson	$<$0.1	$0.2\pm 0.1\pm 0.0$	$0.2\pm 0.1\pm 0.0$
Fake leptons	$<$0.2	$<$0.1	$<$0.2

**Table 10:** Results in the off-*Z* search region SR-loose, in the below-*Z* range ($20<{m_{\ell \ell }}<70$ ${\mathrm {GeV}}$, top) and above-*Z* range (${m_{\ell \ell }}>110$ ${\mathrm {GeV}}$, bottom). Details are the same as in Table 8

	SR-loose *ee*	SR-loose $\mu \mu$	SR-loose same-flavour combined
Below-*Z* ($20<{m_{\ell \ell }}<70$ ${\mathrm {GeV}}$)			
Observed events	509	624	1133
Expected background events	$510 \pm 20 \pm 40$	$680 \pm 20 \pm 50$	$1190 \pm 40 \pm 70$
Flavour-symmetric backgrounds	$490 \pm 20 \pm 40$	$650 \pm 20\pm 50$	$1140 \pm 40 \pm 70$
${Z/\gamma ^{*}+\text {jets}}$	$2.5\pm 0.8\pm 3.2$	$8\pm 2\pm 5$	$11\pm 2\pm 7$
Rare top	$0.3\pm 0.0\pm 0.0$	$0.4\pm 0.0\pm 0.0$	$0.7\pm 0.0\pm 0.0$
*WZ* / *ZZ*	$1.1\pm 0.3\pm 0.1$	$1.2\pm 0.2\pm 0.4$	$2.4\pm 0.4\pm 0.4$
Fake leptons	$16 \pm 4 \pm 2$	$23 \pm 5 \pm 1$	$38 \pm 6 \pm 4$
Above-*Z* (${m_{\ell \ell }}>110$ ${\mathrm {GeV}}$)			
Observed events	746	859	1605
Expected background events	$760 \pm 20 \pm 60$	$830 \pm 20 \pm 70$	$1600 \pm 40 \pm 100$
Flavour-symmetric backgrounds	$730 \pm 20 \pm 60$	$800 \pm 20 \pm 60$	$1500 \pm 40 \pm 100$
${Z/\gamma ^{*}+\text {jets}}$	$0.9\pm 0.2\pm 1.1$	$21\pm 3\pm 24$	$22\pm 3\pm 24$
Rare top	$0.2\pm 0.0\pm 0.0$	$0.2\pm 0.0\pm 0.0$	$0.4\pm 0.0\pm 0.0$
*WZ* / *ZZ* diboson	$0.6\pm 0.2\pm 0.2$	$1.0\pm 0.2\pm 0.1$	$1.6\pm 0.3\pm 0.2$
Fake leptons	$30 \pm 5 \pm 5$	$6.7\pm 3.7\pm 1.7$	$37 \pm 6 \pm 5$

## Interpretation of results

In this section, exclusion limits are shown for the SUSY models described in Sect. 3. The asymptotic $CL_{\mathrm{S}}$ prescription [91], implemented in the HistFitter program [92], is used to determine upper limits at 95 % confidence level (CL). All signal and background uncertainties are taken into account using a Gaussian model of nuisance parameter integration. All uncertainties except that on the signal cross section are included in the limit-setting configuration. The impact of varying the signal cross sections by their uncertainties is indicated separately. Numbers quoted in the text are evaluated from the observed exclusion limit based on the nominal signal cross section minus its $1\sigma$ theoretical uncertainty.

**Fig. 12:**
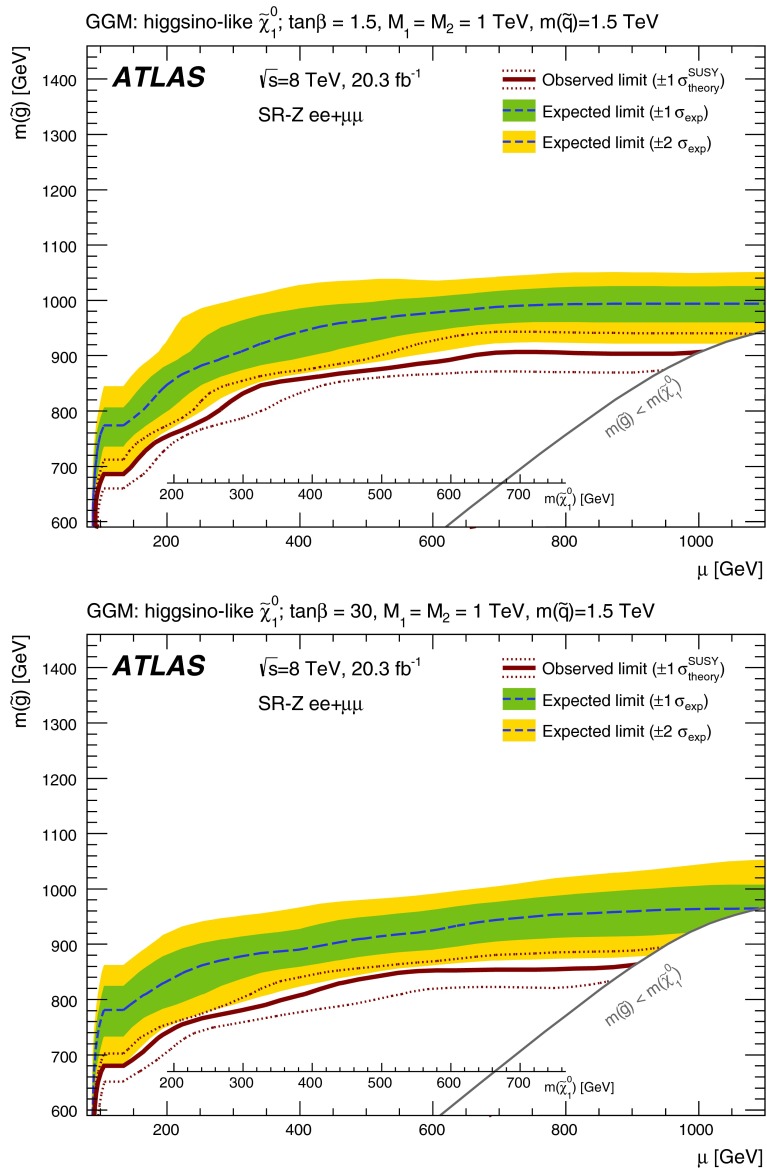
The 95 % CL exclusion limit from the on-*Z* combined same-flavour channels in the $\mu$ versus $m(\tilde{g})$ plane in the GGM model with $\tan \beta =1.5$ (*top*) and $\tan \beta =30$ (*bottom*). The *dark blue dashed line* indicates the expected limits at 95 % CL and the *green* (*yellow*) *bands* show the $\pm 1 \sigma$ ($\pm 2 \sigma$) variation on the expected limit as a consequence of the experimental and theoretical uncertainties on the background prediction. The observed limits are shown by the *solid red lines*, with the *dotted red lines* indicating the limit obtained upon varying the signal cross section by $\pm 1 \sigma$. The region below the *grey line* has the gluino mass less than the lightest neutralino mass and is hence not considered. The value of the lightest neutralino mass is indicated by the *x*-axis inset

For the on-*Z* analysis, the data exceeds the background expectations in the *ee* ($\mu \mu$) channel with a significance of 3.0 (1.7) standard deviations. Exclusion limits in specific models allow us to illustrate which regions of the model parameter space are affected by the observed excess, by comparing the expected and observed limits. The results in SR-*Zee* and SR-*Z*$\mu \mu$ (Table 7) are considered simultaneously. The signal contamination in CR$e\mu$ is found to be at the $\sim$1 % level, and is therefore neglected in this procedure. The expected and observed exclusion contours, in the plane of $\mu$ versus $m(\tilde{g})$ for the GGM model, are shown in Fig. 12. The $\pm 1\sigma _{\mathrm {exp}}$ and $\pm 2\sigma _{\mathrm {exp}}$ experimental uncertainty bands indicate the impact on the expected limit of all uncertainties considered on the background processes. The $\pm 1 \sigma _{\mathrm {theory}}^{\mathrm {SUSY}}$ uncertainty lines around the observed limit illustrate the change in the observed limit as the nominal signal cross section is scaled up and down by the theoretical cross-section uncertainty. Given the observed excess of events with respect to the SM prediction, the observed limits are weaker than expected. In the case of the $\tan \beta =1.5$ exclusion contour, the on-*Z* analysis is able to exclude gluino masses up to 850 ${\mathrm {GeV}}$ for $\mu >450$ GeV, whereas gluino masses of up to 820 ${\mathrm {GeV}}$ are excluded for the $\tan \beta =30$ model for $\mu >600$ GeV. The lower exclusion reach for the $\tan \beta =30$ models is due to the fact that the branching fraction for $\tilde{\chi }^0_{1} \rightarrow Z\tilde{G}$ is significantly smaller at $\tan \beta =30$ than at $\tan \beta =1.5$.

**Fig. 13:**
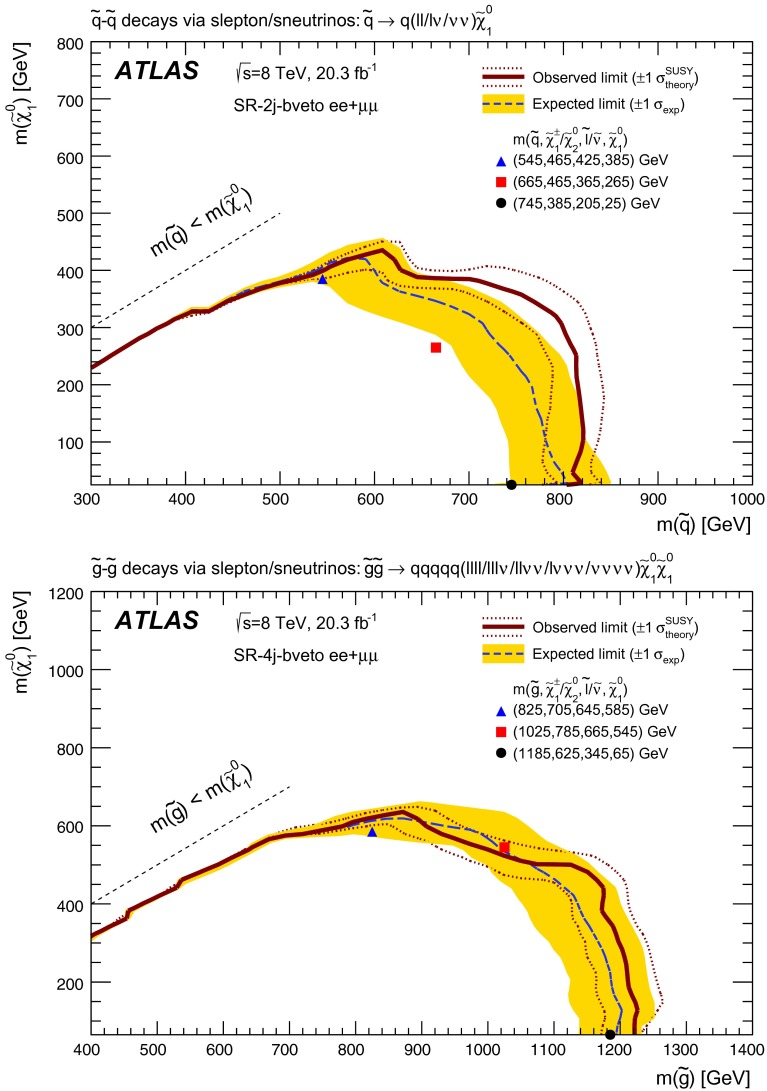
Excluded region in the (*top*) squark-LSP mass plane using the SR-2j-bveto results and (*bottom*) gluino-LSP mass plane using the SR-4j-bveto results. The observed, expected, and $\pm 1\sigma$ expected exclusion contours are indicated. The observed limits obtained upon varying the signal cross section by $\pm 1 \sigma$ are also indicated. The region to the *left* of the *diagonal dashed line* has the squark mass less than the LSP mass and is hence not considered. Three signal benchmark points are shown, with their SUSY particle masses indicated in *parentheses*

For the off-*Z* search, the limits for the squark-pair (gluino-pair) model are based on the results of SR-2j-bveto (SR-4j-bveto). The yields in the combined *ee*+$\mu \mu$ channels are used. Signal contamination in the $e\mu$ control region used for the flavour-symmetry method is taken into account by subtracting the expected increase in the background prediction from the signal yields. For each point in the signal model parameter space, limits on the signal strength are calculated using a “sliding window” approach. The binning in SR-2j-bveto (SR-4j-bveto) defines 45 (21) possible dilepton mass windows to use for the squark-pair (gluino-pair) model interpretation, of which the ten (nine) windows with the best expected sensitivity are selected. For each point in the signal model parameter space, the dilepton mass window with the best expected limit on the signal strength is selected. The excluded regions in the squark-LSP and gluino-LSP mass planes are shown in Fig. 13. The analysis probes squarks with masses up to 780 GeV, and gluinos with masses up to 1170 GeV.

The signal regions in these analyses are also used to place upper limits on the allowed number of BSM events ($N_{\mathrm{BSM}}$) in each region. The observed ($S_\mathrm{obs}^{95}$) and expected ($S_\mathrm{exp}^{95}$) 95 % CL upper limits are also derived using the $CL_{\mathrm{S}}$ procedure. These upper limits on $N_{\mathrm{BSM}}$ can be interpreted as upper limits on the visible BSM cross section ($\langle \epsilon {\sigma }\rangle _{\mathrm{obs}}^{95}$) by normalising $N_{\mathrm{BSM}}$ by the total integrated luminosity. Here $\langle \epsilon {\sigma }\rangle _{\mathrm{obs}}^{95}$ is defined as the product of the signal production cross section, acceptance and reconstruction efficiency. The results are obtained using asymptotic formulae [93] in the case of the off-*Z* numbers. For SR-Z, with a considerably smaller sample size, pseudo-experiments are used. These numbers are presented in Table 11 for the on-*Z* search. Model-independent upper limits on the visible BSM cross section in the below-*Z* and above-*Z* ranges of the five signal regions in the off-*Z* search are presented in Tables 12 and 13, respectively. Limits for the most sensitive dilepton mass windows of SR-2j-bveto and SR-4j-bveto used for the squark- and gluino-pair model interpretations, respectively, are presented in Tables 14 and 15. These tables also present the confidence level observed for the background-only hypothesis $CL_{B}$, and the one-sided discovery *p*-value, $p(s=0)$, which is the probability that the event yield obtained in a single hypothetical background-only experiment (signal, $s=0$) is greater than that observed in this dataset. The $p(s=0)$ value is truncated at 0.5.

**Table 11:** From left to right: 95 % CL upper limits on the visible cross section ($\langle \epsilon {\sigma }\rangle _{\mathrm{obs}}^{95}$) and on the number of signal events ($S_{\mathrm{obs}}^{95}$); the expected 95 % CL upper limit on the number of signal events is denoted by $S_{\mathrm{exp}}^{95}$ and is derived from the expected number of background events (and the $\pm 1\sigma$ uncertainty on the expectation); two-sided $CL_{B}$ value, which is the confidence level observed for the background-only hypothesis; the discovery *p*-value for 0 signal strength *s* ($p(s = 0)$), and the Gaussian significance for the on-*Z* search

Signal region	Channel	$\langle \epsilon {\sigma }\rangle _{\mathrm{obs}}^{95}$ (fb)	$S_{\mathrm{obs}}^{95}$	$S_{\mathrm{exp}}^{95}$	$CL_{B}$	$p\,(s=0)$	Gaussian significance
SR-Z	$ee+\mu \mu$	1.46	29.6	${12}^{+5}_{-2}$	0.998	0.0013	3.0
	*ee*	1.00	20.2	${8}^{+4}_{-2}$	0.998	0.0013	3.0
	$\mu \mu$	0.72	14.7	${9}^{+4}_{-2}$	0.951	0.0430	1.7

**Table 12:** Summary of model-independent upper limits for the five signal regions, in the below-*Z* region ($20<{m_{\ell \ell }}<70$ ${\mathrm {GeV}}$ for SR-loose, $20<{m_{\ell \ell }}<80$ ${\mathrm {GeV}}$ for all other signal regions), in the combined $ee+\mu \mu$ and individual *ee* and $\mu \mu$ channels. Left to right: the observed yield ($N_\mathrm {data}$), total expected background ($N_\mathrm {bkg}$), 95 % CL upper limits on the visible cross section ($\langle \epsilon \sigma \rangle _{\mathrm{obs}}^{95}$) and on the number of signal events ($S_{\mathrm{obs}}^{95}$ ). The fifth column ($S_{\mathrm{exp}}^{95}$) shows the 95 % CL upper limit on the number of signal events, given the expected number (and $\pm 1\sigma$ excursions on the expectation) of background events. The last two columns indicate the $CL_{B}$ value, i.e. the confidence level observed for the background-only hypothesis, and the discovery *p*-value ($p(s = 0)$). For an observed number of events lower than expected, the discovery p-value has been truncated at 0.5

Signal region	Channel	$N_\mathrm {data}$	$N_\mathrm {bkg}$	$\langle \epsilon {\sigma }\rangle _\mathrm{obs}^{95}$ (fb)	$S_\mathrm{obs}^{95}$	$S_\mathrm{exp}^{95}$	$CL_{B}$	$p\,(s=0)$
SR-2j-bveto	$ee+\mu \mu$	54	$50\pm 8\pm 5$	1.38	28.0	${24}^{+8}_{-5}$	0.66	0.35
	*ee*	30	$26\pm 4\pm 3$	0.99	20.1	${18}^{+3}_{-5}$	0.73	0.28
	$\mu \mu$	24	$24\pm 3\pm 3$	0.88	17.8	${18}^{+3}_{-6}$	0.50	0.50
SR-2j-btag	$ee+\mu \mu$	79	$104\pm 11\pm 7$	0.98	19.8	${30}^{+10}_{-9}$	0.06	0.50
	*ee*	40	$49\pm 6\pm 4$	0.85	17.2	${20}^{+8}_{-3}$	0.19	0.50
	$\mu \mu$	39	$56\pm 6\pm 5$	0.63	12.8	${20}^{+9}_{-3}$	0.06	0.50
SR-4j-bveto	$ee+\mu \mu$	6	$8.2\pm 3.1\pm 1.4$	0.38	7.7	${8.3}^{+3.2}_{-1.6}$	0.37	0.50
	*ee*	1	$4.7\pm 1.6\pm 1.1$	0.19	3.9	${5.4}^{+2.0}_{-1.4}$	0.08	0.50
	$\mu \mu$	5	$3.6\pm 1.5\pm 1.0$	0.41	8.4	${6.5}^{+2.9}_{-1.1}$	0.75	0.26
SR-4j-btag	$ee+\mu \mu$	31	$38\pm 6\pm 3$	0.85	17.3	${19}^{+7}_{-4}$	0.25	0.50
	*ee*	14	$18\pm 3\pm 2$	0.51	10.3	${13}^{+6}_{-2}$	0.30	0.50
	$\mu \mu$	17	$20\pm 4\pm 2$	0.54	10.9	${15}^{+4}_{-5}$	0.33	0.50
SR-loose	$ee+\mu \mu$	1133	$1190\pm 40\pm 70$	6.82	138.4	${170}^{+50}_{-40}$	0.28	0.50
	*ee*	509	$510\pm 20\pm 40$	4.88	99.0	${100}^{+40}_{-30}$	0.51	0.48
	$\mu \mu$	624	$680\pm 20\pm 50$	4.10	83.3	${110}^{+40}_{-30}$	0.18	0.50

**Table 13:** Summary of model-independent upper limits for the five signal regions, in the above-*Z* (${m_{\ell \ell }}>110$ ${\mathrm {GeV}}$) dilepton mass range, in the combined $ee+\mu \mu$ and individual *ee* and $\mu \mu$ channels. Details are the same as in Table 12

**Signal region**	Channel	$N_\mathrm {data}$	$N_\mathrm {bkg}$	$\langle \epsilon {\sigma }\rangle _\mathrm{obs}^{95}$ (fb)	$S_\mathrm{obs}^{95}$	$S_\mathrm{exp}^{95}$	$CL_{B}$	$p\,(s=0)$
SR-2j-bveto	$ee+\mu \mu$	55	$73\pm 9\pm 9$	0.96	19.4	${27}^{+8}_{-7}$	0.11	0.50
	*ee*	26	$35\pm 5\pm 4$	0.60	12.1	${18}^{+3}_{-6}$	0.14	0.50
	$\mu \mu$	29	$38\pm 4\pm 8$	0.89	18.1	${20}^{+8}_{-3}$	0.24	0.50
SR-2j-btag	$ee+\mu \mu$	164	$171\pm 14\pm 16$	2.19	44.4	${48}^{+15}_{-12}$	0.39	0.50
	*ee*	83	$81\pm 7\pm 7$	1.45	29.5	${28.3}^{+10}_{-8}$	0.56	0.43
	$\mu \mu$	81	$90\pm 7\pm 14$	1.49	30.2	${36}^{+10}_{-9}$	0.33	0.50
SR-4j-bveto	$ee+\mu \mu$	11	$10\pm 3\pm 2$	0.56	11.4	${10}^{+4}_{-3}$	0.61	0.42
	*ee*	2	$5.7\pm 1.6\pm 1.2$	0.20	4.1	${6.0}^{+2.3}_{-1.8}$	0.13	0.50
	$\mu \mu$	9	$4.5\pm 1.3\pm 1.7$	0.61	12.3	${7.7}^{+2.7}_{-1.6}$	0.91	0.08
SR-4j-btag	$ee+\mu \mu$	41	$36\pm 6\pm 5$	1.27	25.7	${20}^{+9}_{-3}$	0.72	0.29
	*ee*	23	$18\pm 3\pm 2$	0.96	19.5	${15}^{+5}_{-4}$	0.83	0.17
	$\mu \mu$	18	$19\pm 3\pm 4$	0.85	17.2	${17}^{+3}_{-6}$	0.50	0.50
SR-loose	$ee+\mu \mu$	1605	$1600\pm 40\pm 100$	10.58	214.8	${210}^{+30}_{-40}$	0.62	0.40
	*ee*	746	$760\pm 20\pm 60$	6.63	134.6	${140}^{+50}_{-40}$	0.42	0.50
	$\mu \mu$	859	$830\pm 20\pm 70$	8.23	167.1	${150}^{+50}_{-40}$	0.64	0.32

**Table 14:** Summary of model-independent upper limits for SR-2j-bveto, in the combined $ee+\mu \mu$ and individual *ee* and $\mu \mu$ channels, for the ten dilepton mass windows used for the squark-pair interpretation. Details are the same as in Table 12. The last three columns indicate the expected signal yield for three squark-pair model benchmark points; the first (second) number indicates the squark (LSP) mass. The signal yield in square brackets indicates the best selected dilepton mass window for the given benchmark point

$m_{\ell \ell }$ range (GeV)	$N_\mathrm {data}$	$N_\mathrm {bkg}$	$\langle \epsilon {\sigma }\rangle _\mathrm{obs}^{95}$ (fb)	$S_\mathrm{obs}^{95}$	$S_\mathrm{exp}^{95}$	$CL_{B}$	$p\,(s=0)$	$N_{\mathrm {sig}}^{545,385}$	$N_{\mathrm {sig}}^{665,265}$	$N_{\mathrm {sig}}^{74,525}$
20–50	35	$26 \pm 6 \pm 3$	1.32	26.9	${20}^{+7}_{-4}$	0.85	0.15	$17.1 \pm 1.6$	$3.7 \pm 0.4$	$0.6 \pm 0.1$
20–80	54	$50 \pm 8 \pm 4$	1.38	28.0	${24}^{+8}_{-5}$	0.66	0.35	$[38.0 \pm 2.4]$	$10.4 \pm 0.6$	$2.1 \pm 0.2$
50–80	19	$23 \pm 5 \pm 2$	0.63	12.8	${17}^{+3}_{-7}$	0.30	0.50	$20.9 \pm 1.8$	$6.7 \pm 0.5$	$1.5 \pm 0.2$
50–140	34	$46 \pm 7 \pm 6$	0.83	16.9	${20}^{+8}_{-3}$	0.14	0.50	$27.3 \pm 2.0$	$28.5 \pm 1.0$	$6.9 \pm 0.3$
50–200	51	$75 \pm 9 \pm 8$	0.89	18.1	${26}^{+8}_{-7}$	0.05	0.50	$28.2 \pm 2.1$	$50.6 \pm 1.4$	$14.2 \pm 0.5$
110–200	32	$52 \pm 7 \pm 7$	0.69	14.1	${20}^{+8}_{-3}$	0.05	0.50	$2.8 \pm 0.6$	$[34.0 \pm 1.1]$	$10.5 \pm 0.4$
170–260	12	$24 \pm 5 \pm 2$	0.40	8.2	${12}^{+5}_{-4}$	0.03	0.50	$0.4 \pm 0.2$	$14.8 \pm 0.7$	$11.9 \pm 0.4$
170–290	16	$26 \pm 5 \pm 2$	0.43	8.7	${13}^{+5}_{-4}$	0.08	0.50	$0.4 \pm 0.2$	$16.1 \pm 0.8$	$16.8 \pm 0.5$
$>$170	25	$34 \pm 6 \pm 3$	0.68	13.9	${19}^{+3}_{-5}$	0.15	0.50	$0.4 \pm 0.2$	$18.5 \pm 0.8$	$[25.7 \pm 0.6]$
$>$230	16	$13.1 \pm 3.2 \pm 2.3$	0.88	17.9	${14}^{+5}_{-4}$	0.72	0.29	$0.3 \pm 0.2$	$5.0 \pm 0.4$	$17.8 \pm 0.5$

**Table 15:** Summary of model-independent upper limits for SR-4j-bveto, in the combined $ee+\mu \mu$ and individual *ee* and $\mu \mu$ channels, for the nine dilepton mass windows used for the gluino-pair interpretation. Details are the same as in Table 12. The last three columns indicate the expected signal yield for three gluino-pair model benchmark points; the first (second) number indicates the gluino (LSP) mass. The signal yield in square brackets indicates the best selected dilepton mass window for the given benchmark point

$m_{\ell \ell }$ range (GeV)	$N_\mathrm {data}$	$N_\mathrm {bkg}$	$\langle \epsilon {\sigma }\rangle _{\mathrm{obs}}^{95}$ (fb)	$S_{\mathrm{obs}}^{95}$	$S_{\mathrm{exp}}^{95}$	$CL_{B}$	$p\,(s=0)$	$N_{\mathrm {sig}}^{825,585}$	$N_{\mathrm {sig}}^{1,025,545}$	$N_{\mathrm {sig}}^{118,565}$
20–50	4	$3.1 \pm 2.3 \pm 0.9$	0.40	8.2	${7.5}^{+2.0}_{-1.4}$	0.70	0.38	$4.4 \pm 0.7$	$0.8 \pm 0.1$	$0.1 \pm 0.0$
20–80	6	$8.2 \pm 3.1 \pm 1.4$	0.38	7.7	${8.3}^{+3.2}_{-1.6}$	0.37	0.50	$[12.8 \pm 1.1]$	$2.0 \pm 0.2$	$0.2 \pm 0.0$
50–140	6	$8.2 \pm 2.7 \pm 1.4$	0.37	7.5	${8.2}^{+2.9}_{-1.3}$	0.35	0.50	$21.4 \pm 1.4$	$4.9 \pm 0.3$	$0.7 \pm 0.1$
110–200	9	$5.6 \pm 2.3 \pm 1.4$	0.59	12.0	${8.4}^{+3.5}_{-2.0}$	0.85	0.17	$4.2 \pm 0.6$	$6.3 \pm 0.3$	$1.0 \pm 0.1$
140–260	6	$5.0 \pm 2.1 \pm 0.8$	0.43	8.6	${7.4}^{+3.0}_{-1.4}$	0.66	0.38	$1.3 \pm 0.4$	$[8.0 \pm 0.4]$	$1.6 \pm 0.1$
$>$20	17	$18 \pm 4 \pm 3$	0.63	12.8	${14}^{+4}_{-4}$	0.46	0.50	$27.4 \pm 1.6$	$14.4 \pm 0.5$	$7.4 \pm 0.2$
$>$140	7	$7.2 \pm 2.4 \pm 1.3$	0.41	8.3	${8.2}^{+3.1}_{-1.3}$	0.52	0.50	$1.6 \pm 0.4$	$8.6 \pm 0.4$	$6.7 \pm 0.2$
$>$200	2	$4.8 \pm 1.8 \pm 1.1$	0.21	4.2	${5.9}^{+2.2}_{-1.7}$	0.23	0.50	$0.4 \pm 0.2$	$4.2 \pm 0.3$	$6.0 \pm 0.2$
$>$260	1	$2.3 \pm 1.2 \pm 0.7$	0.19	3.9	${4.2}^{+1.9}_{-0.3}$	0.34	0.50	$0.3 \pm 0.2$	$0.7 \pm 0.1$	$[5.1 \pm 0.1]$

## Summary

This paper presents results of two searches for supersymmetric particles in events with two SFOS leptons, jets, and ${E_{\mathrm{T}}^{\mathrm{miss}}}$, using 20.3 fb$^{-1}$ of 8 TeV *pp* collisions recorded by the ATLAS detector at the LHC. The first search targets events with a lepton pair with invariant mass consistent with that of the *Z* boson and hence probes models in which the lepton pair is produced from the decay $Z\rightarrow \ell \ell$. In this search $6.4\pm 2.2$ ($4.2\pm 1.6$) events from SM processes are expected in the $\mu \mu$ (*ee*) SR-Z, as predicted using almost exclusively data-driven methods. The background estimates for the major and most difficult-to-model backgrounds are cross-checked using MC simulation normalised in data control regions, providing further confidence in the SR prediction. Following this assessment of the expected background contribution to the SR the number of events in data is higher than anticipated, with 13 observed in SR-Z $\mu \mu$ and 16 in SR-Z *ee*. This corresponding significances are 1.7 standard deviations in the muon channel and 3.0 standard deviations in the electron channel. These results are interpreted in a supersymmetric model of general gauge mediation, and probe gluino masses up to 900 GeV. The second search targets events with a lepton pair with invariant mass inconsistent with *Z* boson decay, and probes models with the decay chain $\tilde{\chi }_{2}^{0}\rightarrow \ell ^+\ell ^-\tilde{\chi }_{1}^{0}$. In this case the data are found to be consistent with the expected SM backgrounds. No evidence for an excess is observed in the region similar to that in which CMS reported a 2.6$\sigma$ excess [24]. The results are interpreted in simplified models with squark- and gluino-pair production, and probe squark (gluino) masses up to about 780 (1170) GeV.

## Additional results of off-*Z* search

This section provides additional results of the off-*Z* search. The expected backgrounds and observed yields in the below-*Z* and above-*Z* regions for VR, SR-2j-btag, and SR-4j-btag, are presented in Tables 16, 17, and 18, respectively.

**Table 16:** Results in the off-*Z* validation region (VR-offZ), in the below-*Z* range ($20<{m_{\ell \ell }}<70$ ${\mathrm {GeV}}$, top) and above-*Z* range (${m_{\ell \ell }}>110$ ${\mathrm {GeV}}$, bottom). The flavour symmetric, ${Z/\gamma ^{*}+\text {jets}}$ and fake lepton background components are all derived using data-driven estimates described in the text. All other backgrounds are taken from MC simulation. The first uncertainty is statistical and the second is systematic

Below-*Z* ($20<{m_{\ell \ell }}<70$ ${\mathrm {GeV}}$)	VR-offZ *ee*	VR-offZ $\mu \mu$	VR-offZ same-flavour combined
Observed events	465	742	1207
Expected background events	$445 \pm 15 \pm 36$	$682 \pm 23 \pm 53$	$1128 \pm 37 \pm 69$
Flavour-symmetric backgrounds	$425\pm 15 \pm 36$	$661 \pm 22 \pm 53$	$1086 \pm 37 \pm 68$
${Z/\gamma ^{*}+\text {jets}}$	$1.5\pm 0.5\pm 1.6$	$2.7\pm 0.6\pm 3.5$	$4.1\pm 0.8\pm 4.7$
Rare top	$0.1\pm 0.0\pm 0.0$	$0.1\pm 0.0\pm 0.0$	$0.1\pm 0.0\pm 0.0$
*WZ* / *ZZ* diboson	$0.6\pm 0.2\pm 0.1$	$0.8\pm 0.2\pm 0.1$	$1.4\pm 0.2\pm 0.2$
Fake leptons	$18 \pm 4 \pm 2$	$18 \pm 4 \pm 4$	$36 \pm 5 \pm 7$
Above-*Z* (${m_{\ell \ell }}>110$ ${\mathrm {GeV}}$)	VR-offZ *ee*	VR-offZ $\mu \mu$	VR-offZ same-flavour
			combined
Observed events	550	732	1282
Expected background events	$594 \pm 18 \pm 48$	$696 \pm 21 \pm 55$	$1290 \pm 38 \pm 79$
Flavour-symmetric backgrounds	$571 \pm 17 \pm 48$	$684 \pm 21 \pm 55$	$1254 \pm 38 \pm 79$
${Z/\gamma ^{*}+\text {jets}}$	$1.9\pm 0.7\pm 2.0$	$3.8\pm 0.4\pm 6.0$	$5.7\pm 0.8\pm 7.5$
Rare top	$<$0.1	$<$0.1	$<$0.1
*WZ* / *ZZ* diboson	$0.9\pm 0.2\pm 0.2$	$0.9\pm 0.2\pm 0.2$	$1.8\pm 0.3\pm 0.2$
Fake leptons	$21 \pm 4 \pm 2$	$7.9\pm 3.1\pm 2.9$	$29 \pm 5 \pm 4$

**Table 17:** Results in the off-*Z* search region SR-2j-btag, in the below-*Z* range ($20<{m_{\ell \ell }}<80$ ${\mathrm {GeV}}$, top) and above-*Z* range (${m_{\ell \ell }}>110$ ${\mathrm {GeV}}$, bottom). Details are the same as in Table 8

	SR-2j-btag *ee*	SR-2j-btag $\mu \mu$	SR-2j-btag same-flavour combined
Below-*Z* ($20<{m_{\ell \ell }}<80$ ${\mathrm {GeV}}$)			
Observed events	40	39	79
Expected background events	$49 \pm 6 \pm 4$	$56 \pm 6 \pm 5$	$104 \pm 11 \pm 7$
Flavour-symmetric backgrounds	$45 \pm 5 \pm 4$	$49 \pm 6 \pm 5$	$94 \pm 11 \pm 7$
${Z/\gamma ^{*}+\text {jets}}$	$1.8\pm 1.0\pm 0.8$	$3.1\pm 1.3\pm 1.9$	$4.9\pm 1.6\pm 2.2$
Rare top	$0.1\pm 0.0\pm 0.0$	$0.1\pm 0.0\pm 0.0$	$0.1\pm 0.0\pm 0.0$
*WZ* / *ZZ* diboson	$<$0.1	$0.1\pm 0.1\pm 0.1$	$0.1\pm 0.1\pm 0.1$
Fake leptons	$2.3\pm 1.2\pm 0.3$	$3.4\pm 1.9\pm 0.2$	$5.7\pm 2.3\pm 0.6$
Above-*Z* (${m_{\ell \ell }}>110$ ${\mathrm {GeV}}$)			
Observed events	83	81	164
Expected background events	$81 \pm 7 \pm 7$	$90 \pm 7 \pm 14$	$171 \pm 14 \pm 16$
Flavour-symmetric backgrounds	$78 \pm 7 \pm 7$	$77 \pm 7 \pm 7$	$155 \pm 13 \pm 10$
${Z/\gamma ^{*}+\text {jets}}$	$0.8\pm 0.5\pm 0.4$	$11 \pm 1 \pm 13$	$12 \pm 1 \pm 13$
Rare top	$<$0.1	$<$0.1	$0.1\pm 0.0\pm 0.0$
*WZ* / *ZZ* diboson	$<$0.1	$<$0.1	$<$0.1
Fake leptons	$2.4\pm 1.6\pm 0.8$	$1.6\pm 1.3\pm 0.2$	$4.0\pm 2.1\pm 0.7$

**Table 18:** Results in the off-*Z* search region SR-4j-tag, in the below-*Z* range ($20<{m_{\ell \ell }}<80$ ${\mathrm {GeV}}$, top) and above-*Z* range (${m_{\ell \ell }}>110$ ${\mathrm {GeV}}$, bottom). Details are the same as in Table 8

	SR-4j-btag *ee*	SR-4j-btag $\mu \mu$	SR-4j-btag same-flavour combined
Below-*Z* ($20<{m_{\ell \ell }}<80$ ${\mathrm {GeV}}$)			
Observed events	14	17	31
Expected background events	$18 \pm 3 \pm 2$	$20 \pm 4 \pm 2$	$38 \pm 6 \pm 3$
Flavour-symmetric backgrounds	$17 \pm 3 \pm 2$	$18 \pm 3 \pm 2$	$35 \pm 6 \pm 3$
${Z/\gamma ^{*}+\text {jets}}$	$0.5\pm 0.3\pm 0.5$	$0.7\pm 0.3\pm 0.9$	$1.2\pm 0.4\pm 1.1$
Rare top	$<$0.1	$<$0.1	$0.1\pm 0.0\pm 0.0$
*WZ* / *ZZ* diboson	$<$0.1	$<$0.1	$<$0.1
Fake leptons	$0.3\pm 0.6\pm 0.0$	$1.3\pm 1.2\pm 0.0$	$1.6\pm 1.4\pm 0.2$
Above-*Z* (${m_{\ell \ell }}>110$ ${\mathrm {GeV}}$)			
Observed events	23	18	41
Expected background events	$18 \pm 3 \pm 2$	$19 \pm 3 \pm 4$	$36 \pm 6 \pm 5$
Flavour-symmetric backgrounds	$17 \pm 3 \pm 2$	$16 \pm 3 \pm 2$	$33 \pm 6 \pm 3$
${Z/\gamma ^{*}+\text {jets}}$	$0.2\pm 0.1\pm 0.2$	$2.4\pm 0.3\pm 4.0$	$2.7\pm 0.3\pm 4.1$
Rare top	$<$0.1	$<$0.1	$<$0.1
*WZ* / *ZZ* diboson	$<$0.1	$<$0.1	$<$0.1
Fake leptons	$<$0.6	$0.3\pm 0.6\pm 0.1$	$0.0\pm 0.9\pm 0.2$

## Footnotes

1 ATLAS uses a right-handed coordinate system with its origin at the nominal interaction point (IP) in the centre of the detector and the *z*-axis along the beam pipe. The *x*-axis points from the IP to the centre of the LHC ring, and the *y*-axis points upward. Cylindrical coordinates $(r,\phi )$ are used in the transverse plane, $\phi$ being the azimuthal angle around the beam pipe. The pseudorapidity is defined in terms of the polar angle $\theta$ as $\eta =-\ln \tan (\theta /2)$. The opening angle $\Delta R$ in $\eta$–$\phi$ space is defined as $\Delta R=\sqrt{(\Delta \eta )^2+(\Delta \phi )^2}$.

2 The distance of closest approach between a particle object and the primary vertex in the longitudinal (transverse) plane is denoted by $z_{0}$ ($d_{0}$).
